# PDE4D inhibition ameliorates cardiac hypertrophy and heart failure by activating mitophagy

**DOI:** 10.1016/j.redox.2025.103563

**Published:** 2025-02-22

**Authors:** Jing Fu, Congping Su, Yin Ge, Zhou Ao, Li Xia, Yingxiang Chen, Yizheng Yang, Shiwei Chen, Rui Xu, Xiaoyan Yang, Kai Huang, Qin Fu

**Affiliations:** a Department of Pharmacology, School of Basic Medicine, Tongji Medical College, Huazhong University of Science and Technology, Wuhan, China; b Department of Pharmacy, The Central Hospital of Wuhan, Tongji Medical College, Huazhong University of Science and Technology, Wuhan, Hubei, China; c Key Laboratory for Drug Target Research and Pharmacodynamic Evaluation of Hubei Province, Wuhan, China; d Clinic Center of Human Gene Research, Union Hospital, Tongji Medical College, Huazhong University of Science and Technology, Wuhan, China

**Keywords:** Phosphodiesterase 4, Heart failure, Hypertrophy, Mitophagy, PDE4D, cAMP, PKA, CREB, SIRT1

## Abstract

Cyclic adenosine monophosphate (cAMP) plays a major role in normal and pathologic signaling in the heart. Phosphodiesterase 4 (PDE4) is a major PDE degrading cAMP in the heart. There are inconsistencies concerning the roles of the PDE4 isoforms 4B and 4D in regulation of cardiac function. Cardiac PDE4B overexpression is beneficial in remodeling and heart failure (HF), however, the effect of PDE4D and PDE4 inhibitor in HF remains unclear. We generated global and conditional cardiac-specific heterozygous PDE4D knockout mice and adeno-associated virus serotype 9-PDE4D overexpression to determine the role of PDE4D in cardiac hypertrophy and HF. PDE4D upregulation was observed in failing hearts from human and isoproterenol injection and TAC mice. *In vitro*, isoproterenol stimulation increased PDE4D expression via PKA but had no effect on PDE4B expression in cardiomyocytes. PDE4D overexpression per se induced oxidative stress, mitochondrial damage and cardiomyocyte hypertrophy by decreasing PINK1/Parkin-mediated mitophagy through inhibiting cAMP-PKA-CREB-Sirtuin1 (SIRT1) signaling pathway, while PDE4B overexpression did not affect CREB-SIRT1 pathway and mitophagy but exhibited a protective effect on isoproterenol-induced oxidative stress and hypertrophy in cardiomyocytes. PDE4D silencing or inhibition with PDE4 inhibitor roflumilast ameliorated isoproterenol-induced mitochondrial injury and cardiomyocyte hypertrophy. *In vivo*, ISO injection or TAC inhibited cardiac mitophagy and caused cardiac hypertrophy and HF, which were ameliorated by roflumilast or cardiac-specific PDE4D haploinsufficiency. Conversely, cardiac PDE4D overexpression suppressed cardiac mitophagy and abolished the protective effects of global PDE4D haploinsufficiency on TAC-induced cardiac hypertrophy and HF. In conclusion, these studies elucidate a novel mechanism by which sustained adrenergic stimulation contributes to cardiac hypertrophy and HF by increasing PDE4D via cAMP-PKA signaling, which in turn reduces cAMP-PKA activity, resulting in cardiomyocyte hypertrophy and mitochondrial injury via inhibition of CREB-SIRT1 signaling-mediated mitophagy. PDE4D inhibition may represent a novel therapeutic strategy for HF.

## Graphical abstract

**Figure:**
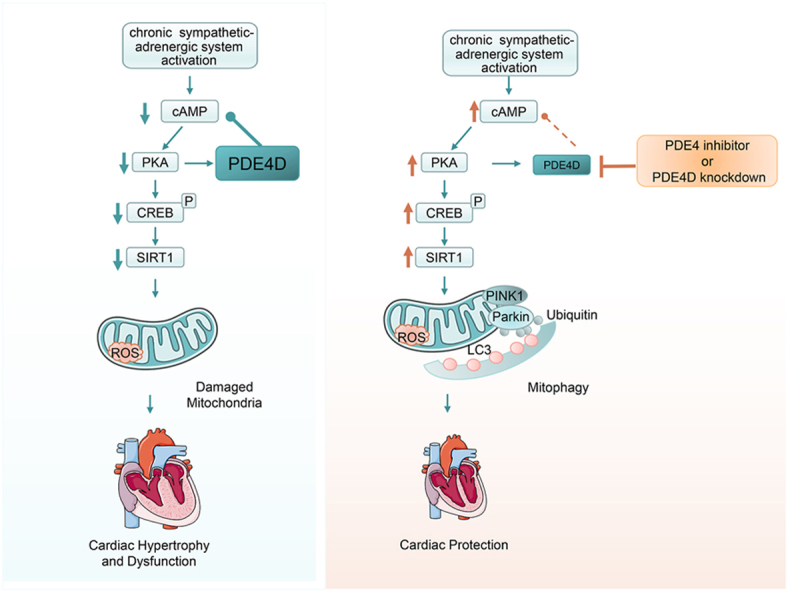

## 1 Introduction

Heart failure (HF) is a leading cause of death worldwide [1]. Although multiple evidence-based therapies have been applied, there are numerous patients with end-stage HF [2], and yet, our understanding of HF remains incomplete and effective treatments and cures are lacking.

In the heart, cyclic adenosine monophosphate (cAMP) represents the strongest mechanism for increasing cardiac function in response to stimulation of cardiac β-adrenergic receptors (β-ARs). The cAMP levels are regulated by phosphodiesterases (PDEs), and PDE4 is a major enzyme responsible for cAMP degradation in the heart [3]. Inhibition of PDE4 activity recovers the β-AR-induced cAMP signal and promotes cardiac contractile response [4]. However, persistent β-AR stimulation also plays an important role in pathological myocardial remodeling and HF, which is associated with cAMP and PKA-mediated protein synthesis and degradation [5], suggesting that selectively inhibiting cAMP-PKA signaling by increasing PDE4 may benefit the failing hearts.

The PDE4 family contains four genes (PDE4A to PDE4D), only PDE4A, PDE4B, and PDE4D are expressed in the heart, each of which regulates different cAMP-dependent signaling in distinct cellular microdomains [6]. At least four PDE4D variants have been detected in human and rodent hearts, including PDE4D3, 4D5, 4D8, and 4D9 [7]. It was reported that PDE4D homozygous null mice showed progressive cardiomyopathy and accelerated HF after myocardial infarction, which is likely due to the reduced PDE4D3 activity in ryanodine receptor signaling complex causes defective ryanodine receptor-channel function [8]. These studies limit the development of PDE4 inhibitors in the treatment of HF. However, in a mouse model of obesity, hyperinsulinemia-induced PDE4D5 promotes HF by limiting ventricular contractility on the basis of reduced myocardial cAMP/PKA activity [9]. Cardiac-specific PDE4D knockdown or pharmacological PDE4 inhibition with roflumilast effectively reversed cardiac dysfunction in high-fat diet mice by promoting cAMP-PKA signaling-induced sarcoplasmic reticulum Ca^2+^-ATPase expression [10]. Recent studies have shown moderate PDE4B overexpression protected against maladaptive remodeling induced by isoproterenol (ISO) and pressure overload, whereas a higher-level PDE4B overexpression led to maladaptive remodeling [11]. The latest study showed that gene therapy with PDE4B3 reduces arrhythmias and HF progression in a mouse model of pressure overload by modulating subcellular cAMP compartmentalization in cardiomyocytes [12]. These discrepant findings reflect the complex role of PDE4 in the development of HF. Given that several PDE4 inhibitors approved for various medical conditions, including chronic obstructive pulmonary disease and asthma [13], potentially inhibiting all splice products of the four PDE4 genes, which may confer cardiovascular risks, it is necessary to further delineate the effect of PDE4 isoforms in HF to develop isoform-specific PDE4 inhibitors.

Given that homozygous PDE4D knock out contributes to heart failure indicating completely inhibition of PDE4 may be detrimental [8], coupled with the ambivalent role of PDE4B overexpression in cardiac pathophysiology depending on PDE4B expression level [11], this study aimed to investigate the effects of moderate PDE4D inhibition in HF. Mice with systemic PDE4D heterozygous knockout or tamoxifen-inducible cardiac-specific PDE4D heterozygous knockout and PDE4D overexpression by adeno-associated virus serotype 9 (AAV9) were generated and subjected to TAC. Our study showed that PDE4D is a novel driver of oxidative stress and cardiac hypertrophy by reducing mitophagy through inhibition of CREB-SIRT1 signaling, which is opposite to the protective effects of PDE4B in HF. These findings suggest that PDE4D inhibition could represent a novel therapeutic strategy for HF treatment.

## 2 Materials and methods

### 2.1 Animals and treatments

All animal procedures were performed in accordance with the National Institutes of Health guidelines and were approved by the animal care and use committee of Tongji Medical College, Huazhong University of Science and Technology (NO.3929). The mice were housed in temperature-controlled cages, fed water and food ad libitum, and maintained under a 12-h light/dark cycle.

All C57BL/6J wild type mice were obtained from Vital River (Beijing, China). For the chronic isoproterenol (ISO) treatment experiment, a total of 36 male mice (8-week-old) were randomly assigned into 4 groups with 9 animals per group. Control and roflumilast group mice were given an equal volume of vehicle or roflumilast (1 mg/kg/day, Selleck, Houston, TX, USA) via oral gavage for 4 weeks. ISO and ISO + roflumilast group mice were treated with ISO (7.5 mg/kg/day, Sigma-Aldrich, MO, USA) intraperitoneal injection and oral gavage with vehicle or roflumilast for 4 weeks.

For the TAC surgery experiment, a total of 45 male 8-week-old mice were randomly assigned to three groups with 15 mice in each group: sham, TAC, and TAC-roflumilast. Each group was administered an equal volume of vehicle or roflumilast (1 mg/kg, Selleck, Houston, TX, USA) by daily oral gavage for 6 weeks after TAC or sham operation. Mice were examined using echocardiography and euthanized by exsanguination under anesthesia.

Mice with a global deletion of PDE4D (PDE4D^−/−^) and PDE4D flox/flox mice (on a C57BL/6J background) were generated by Biocytogen (Beijing, China) using the CRISPR/Cas9 system. PDE4D flox/flox mice were crossed with transgenic mice (MerCreMer) expressing tamoxifen-inducible cardiomyocyte-specific Cre recombinase (Jackson Laboratories) to generate heterozygous cardiac-restricted inducible PDE4D knockout mice (αMHC-Cre+/PDE4Dflox/+, PDE4D^hCKO^) and control Cre-negative littermate mice (αMHC-Cre-/PDE4Dflox/+, PDE4D-WT). One month after transverse aortic constriction (TAC) or sham surgery, mice were subjected to a single intraperitoneal injection of tamoxifen (100 mg/kg/day, MCE, NJ, USA) in corn oil (Aladdin, Shanghai, China) with 10 % ethanol (Sinopharm, Shanghai, China) for 5 consecutive days to induce Cre-mediated PDE4D ablation in the heart: PDE4D-WT Sham, PDE4D-WT TAC, PDE4D^hCKO^ Sham, and PDE4D^hCKO^ TAC, n = 8 mice per group. Mice were examined using echocardiography and were euthanized by exsanguination under anesthesia 12 days after the tamoxifen injection.

In rescue experiments, the recombinant AAV9 vector carrying PDE4D5 with a cardiac troponin T (cTNT) promoter (AAV9-PDE4D5, Vigene Biosciences, Shandong, China) or carrying only cTNT as a negative control (AAV9-NC) was delivered to heterozygous PDE4D global knockout mice (PDE4D^+/−^) and their control littermates (WT) via a bolus tail vein injection at 5 × 10^11^ vector genomes per mouse at 12 days after TAC: WT-AAV9-NC, WT-AAV9-PDE4D5, PDE4D^+/−^AAV9-NC and PDE4D^+/−^AAV9-PDE4D5, n = 6 mice per group. 6 weeks after TAC surgery, the mice were examined using echocardiography and were euthanized by exsanguination under anesthesia anesthetized.

### 2.2 Pressure-overload model

Pressure overload was induced by TAC in 8-week-old male C57BL/6J mice. Mice were anesthetized with 1.5–2% isoflurane, orally intubated, and ventilated with a rodent ventilator. After thoracotomy, a 6–0# silk suture was placed around the 27-G hypodermic needle to constrict the transverse aorta; the needle was quickly removed after ligation. The chest was then closed, and the mice were extubated and allowed to recover from the anesthesia. Sham-operated mice underwent identical procedures, except that the aortic constriction was not placed. The pressure load caused by TAC was verified by the pressure gradient across the aortic constriction measured by echocardiography. At the end of experiments, mice were euthanized by exsanguination under anesthesia.

### 2.3 Neonatal rat ventricular myocytes culture and treatment

Isolation and culture of neonatal rat left ventricular cardiomyocytes (NRVMs) were described previously [9]. After serum starving overnight, the NRVMs were pretreated for 30 min with 1 μM roflumilast or 10 μM PKA inhibitor H89 (LC laboratories, Woburn, MA), and supplemented with 10 μM isoproterenol to induce myocyte injury. Following overnight serum-starving, NRVMs were pretreated for 30 min with 10 μM H89, and supplemented with 10 μM adenylyl cyclase activator forskolin (LC laboratories, Woburn, MA). For PDE4D5 overexpression, cardiac myocytes were transfected with PDE4D5 plasmid or infected with adenovirus GFP-N-terminal-PDE4D5 or mCherry-tagged PDE4D5 which were gifted by Dr. Yang K. Xiang (UC Davis, USA). The blank plasmid or adenovirus containing an empty plasmid was used as a control, respectively. For PDE4A, PDE4B and PDE4D overexpression, cardiac myocytes were transfected with PDE4A (pCMV3-PDE4A-Flag, Human), PDE4B (pCMV3-PDE4B-Flag, Human) and PDE4D (pCMV3-PDE4D-Flag, Human) plasmid or a scramble empty vector as control (Scramble) for 72 h. PINK1 small interfering RNA (siPINK1) or PDE4D small interfering RNA (siPDE4D) was used to knockdown PINK1 or PDE4D, and scramble siRNAs (siNC) were used as controls (Riobio Co., Ltd, Guangzhou, China). For short hairpin RNA (shRNA)-mediated SIRT1 knockdown, SIRT1-shRNA (shSirt1) and scramble shRNA (scramble) were purchased from GeneChem Corporation (Shanghai, China).

### 2.4 Adult cardiomyocyte isolation and treatment

Adult mouse cardiomyocytes were isolated from experimental model mice as described previously [9]. Briefly, mice were anesthetized, their hearts excised and washed briefly, and then mounted on a Langendorff perfusion apparatus. Hearts were digested with 0.5 mg/ml collagenase type II (Worthington, OH, USA) and 0.2 mg/ml proteinase XIV (Sigma-Aldrich, St Louis, MO, USA) perfusion solution. The isolated myocytes were placed in laminin (Sigma-Aldrich, St Louis, MO, USA) pre-coated culture dishes.

### 2.5 Human samples

Human myocardial left ventricular samples were obtained from end-stage failing hearts (non-ischemic dilated cardiomyopathy patients) removed during heart transplantation or from healthy donor hearts that were unsuitable for transplantation for technical reasons (Table S1). All human heart studies were approved by the Human Ethics Committee of the Union Hospital of Huazhong University of Science and Technology (2017-S10005), and informed consent was obtained from all subjects. Biopsies were excised from the left ventricle.

### 2.6 Statistical analysis

All data are presented as mean ± SEM. Sample distribution was analyzed using Shapiro-Wilk normality test. Data with normal distribution were analyzed by unpaired 2-tailed Student *t*-test, a nested *t*-test and ANOVA, one-way ANOVA, or two-way ANOVA with Tukey's post hoc test. Nonparametric tests were used for data sets that failed normality. Statistical tests implemented were calculated using GraphPad Prism 8.0 (GraphPad Software, San Diego, CA). *P* < 0.05 was defined as statistically significant. Representative images were chosen based on their proximity to the mean of each quantification.

An expanded methods section is available in the Supplementary Data.

## 3 Results

### 3.1 PDE4 inhibitor protects against isoproterenol-induced cardiac hypertrophy and heart failure

**Fig. 1:**
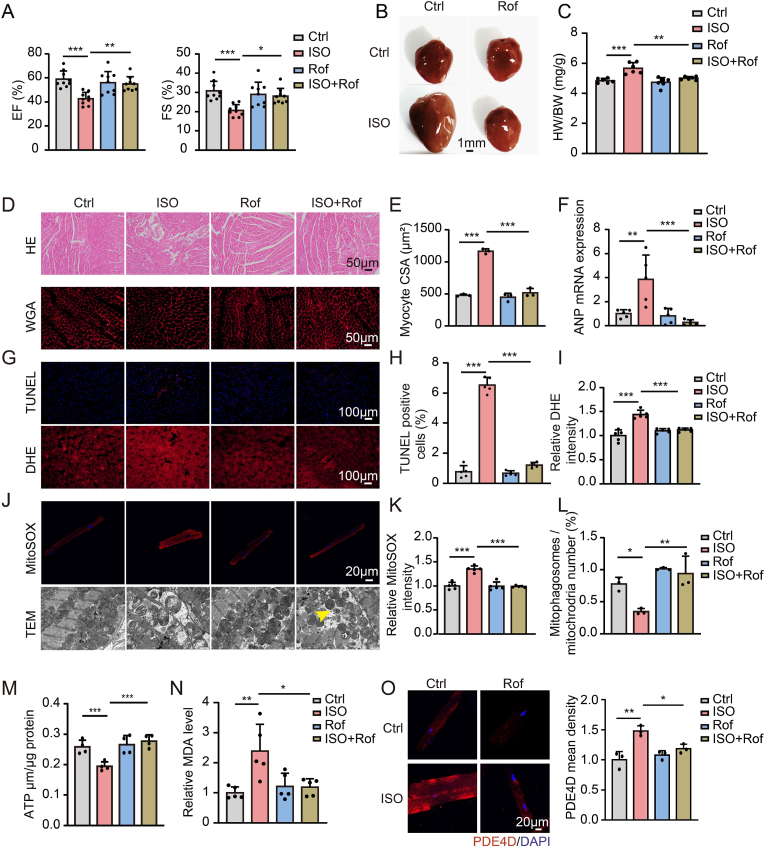
**PDE4 inhibitor roflumilast protects from isoproterenol-induced cardiac hypertrophy and heart failure**. Male, wild-type mice at 8 weeks old were treated with isoproterenol (ISO, 7.5 mg/kg/day) or vehicle via intraperitoneal injection and were administered vehicle or roflumilast (1 mg/kg/day) via oral gavage for 4 weeks. **A,** Ejection fraction (EF, %) and fractional shortening (FS, %) were measured using echocardiography at 4 weeks after ISO treatment; n = 9 mice per group. **B,** Representative images showing gross cardiac morphology of the heart at 4 weeks after ISO treatment. **C,** Ratio of heart weight (HW) to body weight (BW); n = 6 per group. **D,** Representative images of hematoxylin-eosin (HE) and WGA staining of mouse heart sections. **E,** Myocyte cross-sectional area (CSA) was assessed using WGA staining; n = 100 cells per heart and 3 hearts per group. **F,** qRT-PCR shows the hypertrophy marker gene, atrial natriuretic peptide (ANP) mRNA expression in mouse hearts; n = 5 per group. **G-I,** Representative images and quantification of TUNEL and dihydroethidium (DHE) staining of cardiac cross-sections, 5 random fields per heart; n = 5 mice per group. TUNEL, Scale bar, 100 μm; DHE, Scale bar, 100 μm. **J,** Representative images showing MitoSOX staining and mitochondrial morphology determined by transmission electron microscopy in isolated adult cardiomyocytes from indicated mouse groups. MitoSOX, Scale bar, 20 μm; TEM, Scale bar, 1 μm. **K,** Quantification fluorescence intensity of MitoSOX. 1–2 random fields per heart, n = 3 mice per group. **L,** The number of mitophagosomes was quantitated. The yellow arrow indicates representative mitophagosomes. 3 random fields per heart, n = 3 mice per group. **M,** Myocardial ATP levels in the heart tissues; n = 4 mice per group. **N,** Quantitative analysis of relative MDA levels, n = 5 mice per group. **O,** Representative images and quantification of PDE4D expression in isolated adult cardiomyocytes from indicated mouse groups, n = 3 mice per group, Scale bar, 20 μm. All data are presented as mean ± SEM. One-way ANOVA with Tukey's multiple comparison test was used for **A**, **C**, **E**, **F**, **H**, **I**, **L** through **N**, **O**; nested ANOVA analyses were done for **K**.

To examine the role of PDE4 in cardiac hypertrophy and HF, we administered PDE4 inhibitor roflumilast (1 mg/kg/day) or vehicle by oral gavage once daily to mice subjected to chronic ISO (7.5 mg/kg/day) intraperitoneal injection for 1 month. The cardiac function (ejection fraction and fractional shortening) was significantly impaired in ISO-treated mice (Fig. 1A, Table S2). Chronic ISO injection also caused cardiac hypertrophy, indicated by an increase in global heart size, heart weight to body weight ratio (Fig. 1B and C), cardiomyocyte cross-sectional area, as determined by wheat germ agglutinin (WGA) staining (Fig. 1D and E), and increased hypertrophic marker atrial natriuretic peptide (ANP) mRNA expression (Fig. 1F). Myocardial fibers were disrupted and arranged irregularly in ISO-treated mice (Fig. 1D).These ISO-induced changes were markedly attenuated by roflumilast (Fig. 1A through 1F).

Apoptosis (TUNEL staining) and ROS level (DHE staining) also increased in the hearts of ISO-treated mice compared with those in control mice (Fig. 1G through 1I). Mitochondrial ROS levels were assessed by MitoSOX staining of myocytes isolated from ISO-treated mice. The results showed that roflumilast significantly ameliorated apoptosis and ROS levels in ISO-treated mice hearts (Fig. 1G through 1I) and attenuated ISO-induced MitoSOX Red fluorescence (Fig. 1J and K). Moreover, transmission electron microscopy revealed swollen and morphologically altered mitochondria and a decreased ratio of mitophagosome count to mitochondrial count in ISO-treated mouse hearts (Figs. S1A and S1B, Fig. 1J and L). Myocardial ATP production was significantly reduced after ISO treatment (Fig. 1M). Roflumilast increased mitophagosome formation and myocardial ATP content in ISO-treated mouse hearts (Fig. 1J, L and 1M). Roflumilast also prevented the increase of MDA content induced by ISO treatment (Fig. 1N). We found an upregulation of PDE4D mRNA and protein levels in ISO-treated mouse hearts (Figs. S2A and S2B), accompanied by increased PDE4 activity and decreased cAMP content (Figs. S2C and S2D). We did not observe a significant difference in PDE4A or PDE4B expression between mice treated with ISO and the vehicle group (Figs. S2A and S2B). Besides, PDE4D expression was also significantly increased in the adult cardiomyocytes isolated from ISO-treated mice (Fig. 1O). Furthermore, the phosphorylation level of PKA substrates cAMP-responsive element binding protein (CREB) at the PKA-site Ser-133 and phospholamban at the PKA-site Ser-16 was reduced in ISO-treated mouse hearts, suggesting that PKA activity was decreased (Fig. S2B). Roflumilast treatment attenuated PDE4D induction (Fig. 1O, Figs. S2A and S2B) and returned PDE4 activity, cAMP content and phosphorylation of CREB and phospholamban to normal levels in ISO-treated mouse hearts (Figs. S2C and S2D). In contrast, the expression of PDE4A and PDE4B in ISO-treated mouse hearts was not significantly modified by roflumilast (Figs. S2A and S2B). These findings suggested that PDE4 inhibitor may protect against ISO-induced cardiac dysfunction via inhibition of PDE4D.

### 3.2 PDE4D, but not PDE4B, induces hypertrophy and oxidative stress in cardiomyocytes

**Fig. 2:**
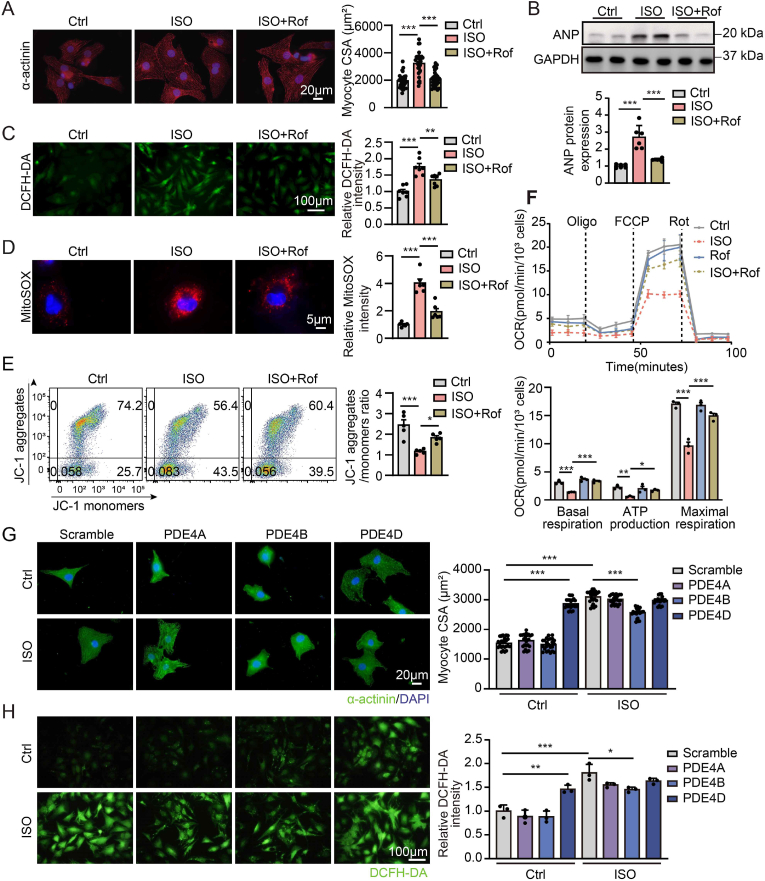
**PDE4D, but not PDE4B, induces hypertrophy and oxidative stress in cardiomyocytes**. **A** through **F,** NRVMs were treated with ISO (10 μM) for 24 h in the presence or absence of roflumilast (1 μM). **A,** Immunofluorescence staining of α-actinin (red) and DAPI (blue) with quantification of myocytes area; n = 24 cells from 3 independent experiments. Scale bar, 20 μm. **B,** Representative immunoblots and quantification of ANP protein expressions in NRVMs; n = 6 independent experiments. **C,** Quantification and representative image of intracellular ROS levels with DCFH-DA staining; n = 7 independent experiments. **D,** Quantification and representative image of mitochondrial reactive oxygen species (ROS) levels with MitoSOX staining. n = 6 independent experiments. **E,** Representative histograms of mitochondrial membrane potential (ΔΨm) measured by flow cytometry with JC-1 probe and the ratio of JC-1 aggregates to monomers were quantified; n = 5 independent experiments. **F,** Seahorse mitochondrial stress assay of NRVMs treated with ISO for 24h in the presence or absence of roflumilast. Basal respiration, ATP production and maximal respiration were analyzed normalized on the amount of total cell number/well; n = 3 independent experiments. **G** and **H,** NRVMs were transfected with PDE4A, PDE4B, PDE4D plasmid or scramble plasmid for 48 h followed by ISO (10 μM) or vehicle stimulation for another 24 h. **G,** Immunofluorescence staining of α-actinin (green) and DAPI (blue) with quantification of myocytes area; n = 24 cells from 3 independent experiments. Scale bar, 20 μm. **H,** DCFH-DA staining with quantification of intracellular ROS levels; n = 3 independent experiments. Scale bar, 100 μm. All data are presented as mean ± SEM. One-way ANOVA with Tukey's multiple comparison test was used for **B** through **F**, and **H**; nested ANOVA analyses were done for **A** and **G**.

The cardiac protective effects of PDE4 inhibitor were further verified in vitro. The NRVMs were treated with ISO for 24 h in the presence or absence of roflumilast. ISO treatment increased NRVMs surface area and ANP expression, which were significantly attenuated by roflumilast (Fig. 2A and B). Roflumilast also protected cardiomyocytes from ISO-induced generation of intracellular and mitochondrial ROS as measured using DCFH-DA and MitoSOX probes (Fig. 2C and D), respectively, and decreased mitochondrial membrane potential (MMP) as detected by JC-1 staining (Fig. 2E). Seahorse analysis showed that ISO stimulation significantly suppressed the basal respiration value, ATP production and maximal respiration, which were reversed by roflumilast, indicating that roflumilast protected against ISO-induced mitochondrial injury in cardiomyocytes (Fig. 2F). Roflumilast alone did not impair cardiomyocytes (Figs. S3A and S3B) or affect their size (Fig. S3C), ROS level, or MMP (Figure S3D through S3F). In parallel with the in vivo data, ISO stimulation significantly increased PDE4D mRNA and protein levels in NRVMs, but no changes were observed in PDE4A and PDE4B expression levels (Figs. S4A and S4B). Roflumilast inhibited ISO-induced increase in PDE4D mRNA and protein expression, but had no effects on PDE4A and PDE4B expression levels (Figs. S4A and S4B). Further detection showed that both ISO and adenylyl cyclase activator forskolin increased PDE4D expression, which were attenuated by PKA inhibitor H89 (Figs. S4C and S4D). These results were consistent with previous study that PDE4 is activated in response to cAMP-dependent PKA activation, thus establishing a negative feedback loop that balances intracellular cAMP levels [14].

Our results showed that overexpression of PDE4D per se increased myocyte cell area and intracellular ROS level (Fig. 2G and H). However, PDE4B overexpression had no effect on cell surface area and intracellular ROS level but partially inhibited the increase in cell surface area and intracellular ROS level induced by ISO stimulation (Fig. 2G and H), which is consistent with a previous report that cardiac PDE4B is beneficial to counteract the detrimental effect of excessive sympathetic system activation in HF [11]. These findings indicated that ISO induces PDE4D expression via PKA and promotes cardiomyocyte hypertrophy and oxidative stress.

### 3.3 PDE4D5 induces hypertrophy and oxidative stress and decreases PINK1/Parkin mitophagy pathway in cardiomyocytes

**Fig. 3:**
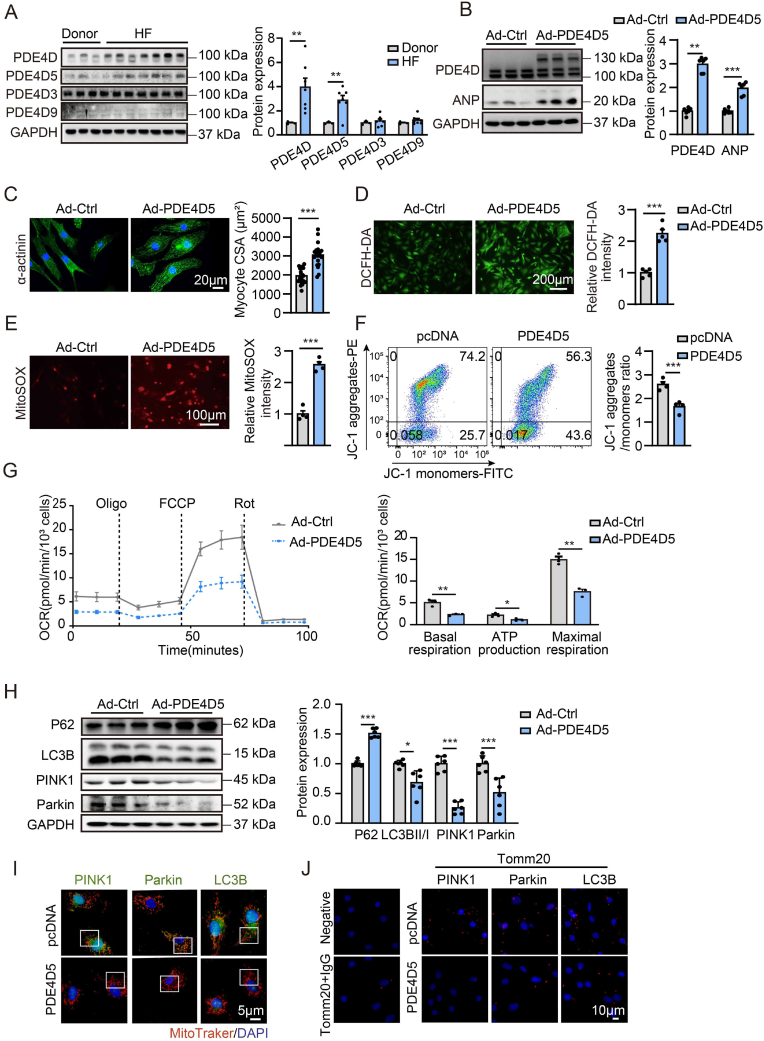
**PDE4D5 expression is upregulated in human failing hearts and PDE4D5 induces hypertrophy and oxidative stress and inhibits mitophagy in NRVMs**. **A,** Representative immunoblots and quantification of PDE4D and PDE4D variants (PDE4D5, PDE4D3, and PDE4D9) expression in left ventricle tissues from healthy donors (n = 3) and heart failure (HF) patients (n = 7). NRVMs were infected with mCherry-PDE4D5 adenovirus (Ad-PDE4D5) or control adenovirus (Ad-Ctrl) for 48 h **(B** through **D**). **B,** The protein expressions of PDE4D and hypertrophy marker gene, ANP in NRVMs; n = 6 independent experiments. **C,** Immunofluorescence staining of α-actinin (green) and DAPI (blue) in NRVMs and quantification of myocytes area; n = 24 cells from 3 independent experiments. Scale bar, 20 μm. **D,** DCFH-DA staining and quantification of ROS levels in NRVMs; n = 5 independent experiments. Scale bar, 200 μm. **E,** MitoSOX staining and quantification of mitochondrial ROS levels in NRVMs infected with GFP-N-terminal-PDE4D5 adenoviruses (Ad-PDE4D5) or control adenovirus (Ad-Ctrl) for 48 h; n = 4 independent experiments. Scale bar, 100 μm. **F,** Fluorescence activated cell sorting (FACS) analyses and quantification of ΔΨm by the ratio of JC-1 aggregates (red) to monomers (green) in NRVMs transfected with plasmid expressing PDE4D5 or pcDNA vector control; n = 4 independent experiments. **G,** Seahorse mitochondrial stress assay of NRVMs infected with Ad-PDE4D5 or Ad-Ctrl for 48 h. Basal respiration, ATP production and maximal respiration were analyzed normalized on the amount of total cell number/well; n = 3 independent experiments. **H,** Relative protein expressions and quantification of P62, LC3BII/I, PINK1, and Parkin in NRVMs infected with mCherry-PDE4D5 adenovirus (Ad-PDE4D5) or control adenovirus (Ad-Ctrl) for 48 h; n = 6 independent experiments. **I,** Representative immunofluorescence co-staining of MitoTracker (red) with PINK1 (green), Parkin (green), or LC3B (green) in NRVMs transfected with plasmid expressing PDE4D5 or pcDNA vector control with quantification (enlarged image, single channels and quantification are shown in Figure S6A through S6C in the Data Supplement); n = 24 cells from 6 independent experiments. Scale bar, 5 μm. **J,** Representative fluorescence images of PLA signals after labeling with antibodies against Tomm20 and IgG, Tomm20 and PINK1, Tomm20 and Parkin, and Tomm20 and LC3B in NRVMs transfected with plasmid expressing PDE4D5 or pcDNA vector, respectively. Positive PLA signal (red), DAPI (blue) (quantification are shown in Fig. S6D in the Data Supplement); n = 5 independent experiments. Scale bar = 10 μm. All data are presented as mean ± SEM. Unpaired *t*-test with welch's correction was used for **A,** Mann-Whitney *U* test was used for **B,** unpaired 2-tailed Student *t*-test was used for **D** through **H**, nested *t*-test was done for **C**.

We further found that PDE4D protein expression was elevated in failing human hearts compared with that in non-failing controls (Fig. 3A). PDE4D5 variant expression was significantly increased, whereas PDE4D3 and PDE4D9 expression were comparable between the donor and HF groups (Fig. 3A). Analysis of the Gene Expression Omnibus (GEO) database (GSE120895) revealed that PDE4D mRNA levels were significantly increased in DCM patients with LV systolic dysfunction (LVEF <45 %) and symptoms of HF compared with control with normal (Fig. S5A). Consistent with these research results, single-cell RNA-seq analysis of human cardiomyocytes from normal subjects and patients with DCM also demonstrated upregulation of PDE4D mRNA in the HF heart samples (GSE95140) (Fig. S5B). PDE4D5 overexpression increased the protein expression of the hypertrophic marker ANP and cell surface area in NRVMs (Fig. 3B and C). Furthermore, PDE4D5 overexpression increased production of intracellular ROS and mitochondrial ROS and decreased MMP (Fig. 3D through 3F). Seahorse analysis showed that PDE4D5 overexpression significantly suppressed the basal respiration value, ATP production, and maximal respiration in cardiomyocytes (Fig. 3G). These data suggest that PDE4D5 is upregulated under failing conditions and may be involved in cardiac hypertrophy and mitochondrial damage pathogeneses.

Impaired mitochondrial function has been implicated in cardiac hypertrophy and HF. The PINK1/Parkin mitophagy pathway plays an important role in maintaining mitochondrial homeostasis and function in cardiomyocytes [15,16]. We found that PDE4D5 overexpression suppressed the protein expression of PINK1 and Parkin in cardiomyocytes (Fig. 3H). The autophagy hallmarks LC3B and P62 were detected, with decreased LC3BII/I ratio and increased P62 expression in PDE4D5 overexpression cardiomyocytes (Fig. 3H). Immunofluorescence analysis revealed that PDE4D5 overexpression reduced the colocalization of PINK1, Parkin and LC3B with MitoTracker in cardiomyocytes (Fig. 3I, Figure S6A through S6C). As another method to sustain the evidence of mitochondrial localization of PINK1, Parkin and LC3B, we analyzed a potential interaction between PINK1/Parkin/LC3B and a well-established mitochondrial protein Tomm20 using the proximity ligation assay (PLA) that detects proximity of proteins 40 nm or less distant from each other. The cardiomyocytes co-labeled with Tomm20-PINK1, Tomm20-Parkin, and Tomm20-LC3B showed PLA signals, while control cells had no signal, representing that PINK1, Parkin and LC3B in close proximity to mitochondria (Tomm20), respectively (Fig. 3J). The PLA assay showed PDE4D5 overexpression reduced interactions between PINK1/Parkin/LC3B and Tomm20 in cardiomyocytes, respectively (Fig. 3J, Fig. S6D), confirmed a decrease in mitophagy in PDE4D5 overexpression cardiomyocytes. In contrast, PDE4B overexpression had no effects on the expression of LC3BII/I, P62, PINK1 and Parkin, and colocalization of PINK1, Parkin and LC3B with MitoTracker (Figure S7A through S7D). Taken together, these data strongly suggest that mitophagy impairment is involved in PDE4D-induced cardiomyocyte hypertrophy and mitochondrial injury.

### 3.4 PDE4 inhibitor protects cardiomyocytes from hypertrophic and oxidative stress by inducing mitophagy through SIRT1/PINK1/Parkin pathway

**Fig. 4:**
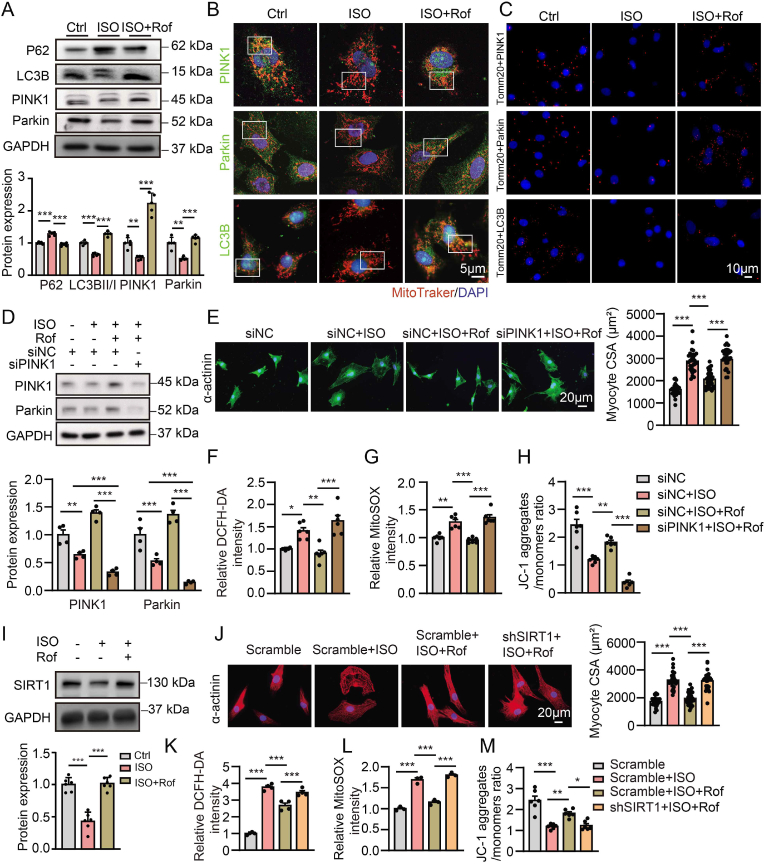
**PDE4 inhibitor protects against cardiomyocyte hypertrophy and mitochondrial dysfunction by inducing mitophagy through SIRT1 activation**. **A** through **M,** NRVMs were treated with vehicle or ISO (10 μM) for 24 h in the presence or absence of roflumilast (1 μM). **A,** Representative immunoblots and quantification of P62, LC3BII/I, PINK1 and Parkin protein expressions in NRVMs; n = 5 independent experiments. **B**, Representative immunofluorescence co-staining of MitoTracker (red) with PINK1 (green), Parkin (green) or LC3B (green) in NRVMs (single channels and quantification are shown in Figure S8A through S8C in the Data Supplement); n = 6 independent experiments. Scale bar, 5 μm. **C**, Representative fluorescence images of PLA signals after labeling with antibodies against Tomm20 and PINK1, Tomm20 and Parkin, and Tomm20 and LC3B in NRVMs treated with ISO for 24 h in the presence or absence of roflumilast, respectively. Positive PLA signal (red), DAPI (blue) (quantification are shown in Fig. S8D in the Data Supplement); n = 5 independent experiments. Scale bar = 10 μm. NRVMs were transfected with PINK1 siRNA (siPINK1) or negative control siRNA (siNC) for 48 h and treated with vehicle or ISO (10 μM) for another 24 h in the presence or absence of roflumilast (1 μM) (**D** through **H**). **D,** Immunoblotting analysis of PINK1 and Parkin protein expression in NRVMs, n = 4 independent experiments. **E**, Immunofluorescence staining of α-actinin (green) and DAPI (blue) with quantification of myocytes area in NRVMs of indicated groups; n = 24 cells from 3 independent experiments. Scale bar, 20 μm. FACS analyses and quantification of cellular ROS levels with DCFH-DA staining (**F**), mitochondrial ROS levels with MitoSOX staining (**G**), and the level of mitochondrial membrane potential (ΔΨm) by the ratio of JC-1 aggregates to JC-1 monomers (**H**); n = 6 independent experiments. Representative images are shown in Figure S9B through S9D in the Data Supplement (**F** through **H**). **I,** Representative immunoblots and quantification of SIRT1 protein expressions in NRVMs; n = 6 independent experiments. **J-M,** NRVMs were transfected with SIRT1 shRNA (shSIRT1) or scramble shRNA (scramble) for 48 h and treated with vehicle or ISO (10 μM) for another 24 h in the presence or absence of roflumilast (1 μM). **J,** Immunofluorescence staining of α-actinin (red) and DAPI (blue) in NRVMs and quantification of myocytes area in NRVMs; n = 24 cells from 3 independent experiments. Scale bar, 20 μm. **K** through **M,** FACS analyses and quantification of cellular ROS levels with DCFH-DA staining (**K**, n = 4 independent experiments), mitochondrial ROS levels with MitoSOX staining (**L**, n = 3 independent experiments), and the level of ΔΨm by the ratio of JC-1 aggregates to monomers (**M**, n = 5 independent experiments). Representative images are shown in Figure S10C through S10E in the Data Supplement (**K** through **M**). All data are presented as mean ± SEM. One-way ANOVA with Tukey's multiple comparison test was used for **A** and **D**, **F** through **I**, and **K** through **M**; nested ANOVA analyses were done for **E** and **J**.

In NRVMs, ISO treatment also led to an increase in P62 expression and a marked decrease in the protein expression of LC3BII/I, PINK1 and Parkin (Fig. 4A), colocalization of mitochondria with PINK1, Parkin and LC3B (Fig. 4B, Figure S8A through S8C), which were largely inhibited by roflumilast (Fig. 4A and B and Figure S8A through S8C). Quantification of PLA signal showed that ISO stimulation decreased the association of Tomm20-PINK1, Tomm20-Parkin, and Tomm20-LC3B, which were reversed by roflumilast treatment (Fig. 4C and Fig. S8D), suggesting that roflumilast activated mitophagy in ISO-treated cardiomyocytes.

To investigate whether PINK1/Parkin-mediated mitophagy is involved in the protective effects of roflumilast, we knocked down PINK1 expression using siRNA (Fig. S9A). The roflumilast-induced increase in PINK1 and Parkin expression was abolished by PINK1 siRNA (Fig. 4D). Roflumilast effects on cell size, oxidative stress, and mitochondrial damage in ISO-treated NRVMs were blocked by PINK1 siRNA transfection (Fig. 4E through 4H, Figure S9B through S9D). Additionally, silencing of PINK1 inhibited roflumilast-induced colocalization of Parkin and LC3B with MitoTracker in cardiomyocytes exposed to ISO stimulation (Figs. S9E and S9F). These results suggest that PDE4 inhibitor protects cardiomyocytes against hypertrophy and mitochondrial dysfunction at least partially via PINK1/Parkin-mediated mitophagy.

SIRT1, a NAD-dependent histone deacetylase, confers mitochondrial protection by improving PINK1/Parkin-mediated mitophagy [17]. Consistent with this, SIRT1 silencing did indeed inhibit PINK1 and Parkin expression in cardiomyocytes (Fig. S10A). Given that increased expression of SIRT1, PINK1 and Parkin was observed in the hearts from ISO-challenged mice treated with roflumilast (Fig. S10B), we then investigated whether SIRT1 is involved in PDE4 inhibitor-mediated PINK1/Parkin mitophagy pathway. ISO treatment significantly decreased the expression of SIRT1 in cardiomyocytes, which was attenuated by roflumilast (Fig. 4I). After treatment with SIRT1 shRNA, the protective effects of roflumilast on ISO-induced cardiac hypertrophy, oxidative stress, and mitochondrial damage were abolished (Fig. 4J through 4M, Figure S10C through S10E), and roflumilast-induced colocalization of PINK1, Parkin and LC3B with MitoTracker was also attenuated (Figure S11A through S11C). These results suggest that PDE4 inhibitor exerts cardioprotective effects by activating mitophagy via SIRT1-mediated PINK1/Parkin signaling pathway.

The cAMP responsive element binding protein (CREB) serves as a critical transcription factor in cAMP/PKA signaling axis and is recruited to SIRT1 chromatin to directly promote SIRT1 expression [18]. Our previous study demonstrated that PDE4D inhibition promotes SIRT1 expression via cAMP-PKA-CREB signaling pathway [10]. Phosphorylation at serine 133 residue and nuclear translocation determine CREB activity. In line with this, chronic ISO stimulation reduced nuclear CREB expression, which was attenuated by roflumilast (Fig. S12A). Moreover, PDE4D overexpression significantly reduced CREB phosphorylation and expression of SIRT1 (Figs. S12B and S12C), while PDE4B overexpression did not affect the phosphorylation level of CREB and SIRT1 expression (Figs. S12B and S12D). Findings similar to these have been reported previously that ablation of PDE4D but not PDE4B increases the magnitude of PKA phosphorylation of CREB [19]. We further found that PDE4D staining, but not PDE4B, had overlap with CREB (Fig. S12E). This may underlie the inability of PDE4B to dephosphorylate CREB could in part be responsible for the different roles of PDE4B and PDE4D in cardiomyocytes.

### 3.5 PDE4D silencing protects cardiomyocytes from ISO-induced hypertrophic and mitochondrial dysfunction by inducing mitophagy via SIRT1 activation

**Fig. 5:**
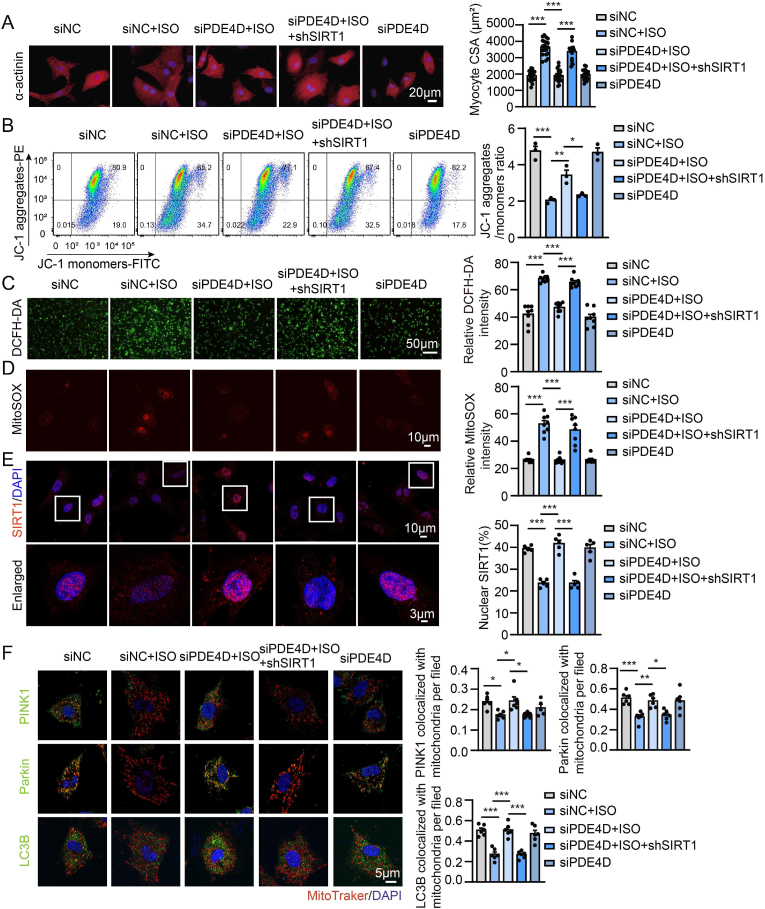
SIRT1 knockdown abolishes the protection of PDE4D silencing on ISO-induced hypertrophy and mitochondrial dysfunction in cardiomyocytes. **A** through **F,** NRVMs were transfected with PDE4D siRNA (siPDE4D) and SIRT1 shRNA or negative control siRNA (siNC) for 48 h followed by vehicle or ISO (10 μM) treatment for another 24 h. **A,** Immunofluorescence staining of α-actinin (red) and DAPI (blue) and quantification of myocytes area in NRVMs; n = 24 cells from 3 independent experiments. Scale bar, 20 μm. **B,** FACS analyses of ΔΨm in NRVMs. A loss of ΔΨm was demonstrated by the ratio of JC-1 aggregates to JC-1 monomers; n = 3 independent experiments. **C,** DCFH-DA staining and quantification of cellular ROS levels in NRVMs; n = 8 independent experiments. Scale bar, 50 μm. **D,** MitoSOX staining and quantification of mitochondrial ROS levels in NRVMs; n = 8 independent experiments. Scale bar, 10 μm. **E**, Representative immunofluorescence images of SIRT1 staining (red) and DAPI staining (blue) in NRVMs with quantification of nuclear SIRT1; n = 5 independent experiments. Scale bar, 10 μm. **F,** Representative immunofluorescence co-staining and Pearson's correlation coefficient for colocalization of MitoTracker (red) with PINK1 (green), Parkin (green) or LC3B (green) in NRVMs (single channels are shown in Figure S13A through S13C); n = 24 cells from 6 independent experiments. Scale bar, 5 μm. All data are presented as mean ± SEM. One-way ANOVA with Tukey's multiple comparison test was used for **B** through **F**; nested ANOVA analyses were done for **A**.

We further validated the effects of PDE4D silencing on cardiomyocyte hypertrophy and mitophagy. ISO-induced cardiomyocyte hypertrophy and mitochondrial dysfunction were attenuated by PDE4D silencing (Fig. 5A through 5D). ISO-reduced nuclear SIRT1 expression was also restored by PDE4D silencing (Fig. 5E). The beneficial effects of PDE4D silencing included improvement in cardiomyocyte hypertrophy, oxidative stress, and mitochondrial damage, which were attenuated by SIRT1 knockdown with shRNA (Fig. 5A through 5D). PDE4D silencing also had an inhibitory effect on ISO-reduced colocalization of PINK1/Parkin/LC3B and mitochondria, which was abolished by SIRT1 shRNA (Fig. 5F and Fig. S13). Together, these results are consistent with the cardioprotective effects of roflumilast, suggesting that reducing the abundance of PDE4D may provide an effective strategy to protect against cardiac hypertrophy and mitochondrial dysfunction by modulating SIRT1-mediated mitophagy.

### 3.6 PDE4 inhibitor attenuates transverse aortic constriction (TAC)-induced cardiac hypertrophy and heart failure

**Fig. 6:**
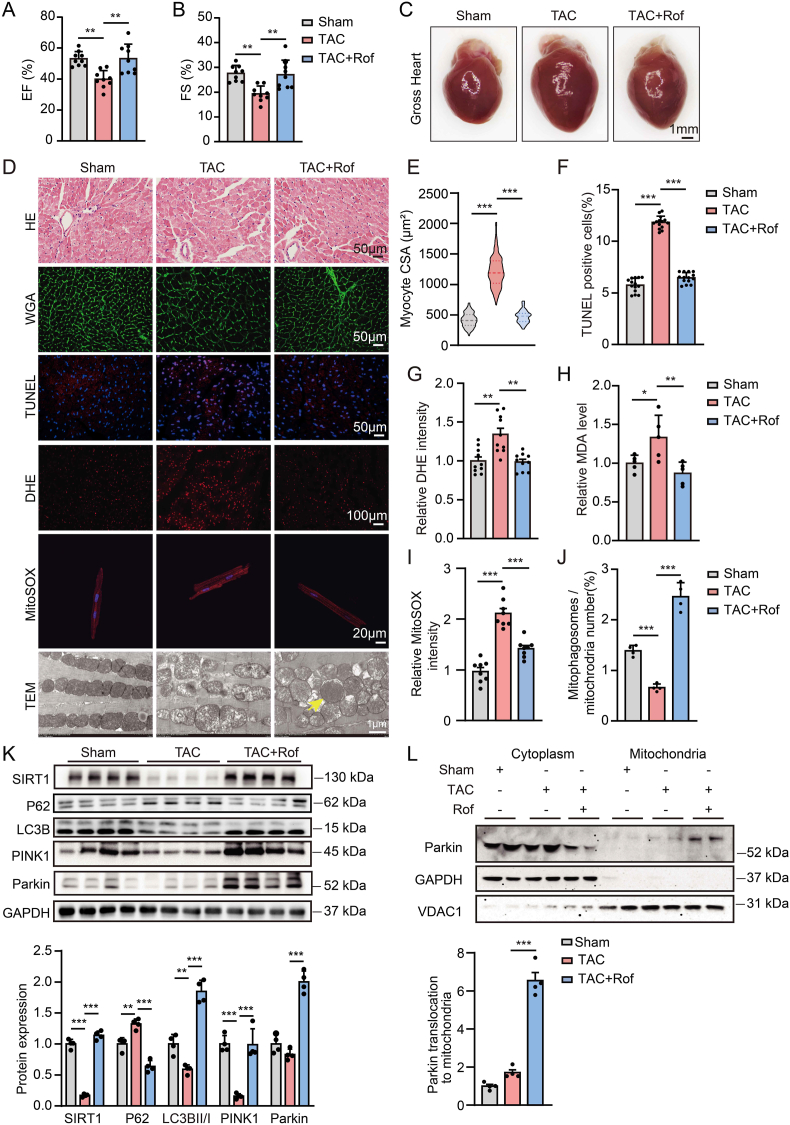
**PDE4 inhibitor attenuates TAC-induced cardiac hypertrophy and heart failure**. Mice were subjected to TAC or sham operation and administered vehicle or roflumilast (1 mg/kg/day) via oral gavage for 6 weeks. Ejection fraction (**A**) and fractional shortening (**B**) were measured using echocardiography at 6 weeks after TAC; n = 9 mice per group. **C,** Representative images showing gross cardiac morphology of the heart. **D,** Representative images of HE, staining of WGA, TUNEL, DHE and MitoSOX and transmission electron microscopy in mouse hearts in the indicated groups. **E,** CSA was assessed using WGA staining; n = 100 cells per heart and 3 hearts per group. Quantification of TUNEL (**F**) and DHE (**G**) staining, 3–5 random fields per heart; n = 3 mice per group. TUNEL, Scale bar, 50 μm; DHE, Scale bar, 100 μm. **H.** Quantitative analysis of relative MDA levels, n = 5 mice per group. **I,** Mitochondrial ROS level was measured using MitoSOX staining in isolated adult cardiomyocytes from indicated groups, 2–3 random fields per heart, n = 3 mice per group; Scale bar, 20 μm. **J,** Mitochondrial morphology was determined by transmission electron microscopy and the number of mitophagosomes was quantitated. The yellow arrow indicates representative mitophagosome observed by transmission electron microscopy; 1–2 random fields per heart, n = 3 mice per group. Scale bar, 1 μm. **K,** Representative immunoblots and quantification of SIRT1, P62, LC3BII/I, PINK1 and Parkin in heart tissues; n = 4 mice per group. **L,** Parkin expression in mitochondria and cytoplasm of mouse hearts; n = 4 mice per group. All data are presented as mean ± SEM. One-way ANOVA with Tukey's multiple comparison test was used for **A** and **B**, **H**, **K** and **L**; nested ANOVA analyses were done for **E** through **G**, **I** and **J**.

We further examine the effect of PDE4 inhibition in response to pressure overload-induced cardiac hypertrophy and HF. The animals were treated with roflumilast or vehicle (1 mg/kg/day) by oral gavage once daily for 6 weeks after TAC (Fig. S14A). TAC mice displayed contractile dysfunction (Fig. 6A and B, Table S3) with increased global heart size, cardiomyocyte cross-sectional area (Fig. 6C through 6E), heart weight/body weight ratio and elevated mRNA levels of hallmark hypertrophic markers ANP and BNP (Figs. S14B and S14C). As with the model of ISO-induced HF, TAC mice also exhibited cardiac apoptosis and increased intracellular ROS and MDA level (Fig. 6D and F through 6H). These TAC-induced changes were markedly attenuated by roflumilast (Fig. 6A through 6H). Moreover, roflumilast reduced mitochondrial ROS level and improved mitochondrial morphology, increased mitophagosome formation and myocardial ATP content in TAC mice (Fig. 6D, 6I, 6J and Figure S14D and S14E). The cAMP level was reduced and the PDE4 activity was increased in TAC mouse hearts (Figs. S14F and S14G). Both mRNA and protein expression of PDE4D were increased, while the expression of PDE4A and PDE4B was not affected by TAC (Figs. S14H and S14I). Roflumilast returned cAMP content, PDE4 activity and PDE4D protein expression to normal levels in TAC mouse hearts (Figure S14F through S14I).

TAC also significantly reduced SIRT1 expression, which was attenuated by roflumilast (Fig. 6K). Moreover, roflumilast could increase the expression of mitophagy-related proteins PINK1, Parkin and LC3BII/I and decrease P62 expression in TAC mouse hearts (Fig. 6K). Roflumilast also significantly increased mitochondrial Parkin expression and colocalization of Parkin and the mitochondrial outer membrane protein Tomm20 (Fig. 6L and Fig. S15A), and promoted Parkin auto-ubiquitination in TAC mouse hearts (Fig. S15B). These data strongly suggest that PDE4 inhibitor roflumilast attenuates the development of TAC-induced HF by promoting cardiac mitophagy via SIRT1-mediated PINK1/Parkin pathway.

### 3.7 Downregulation of PDE4D protects against TAC-induced cardiac hypertrophy and heart failure

**Fig. 7:**
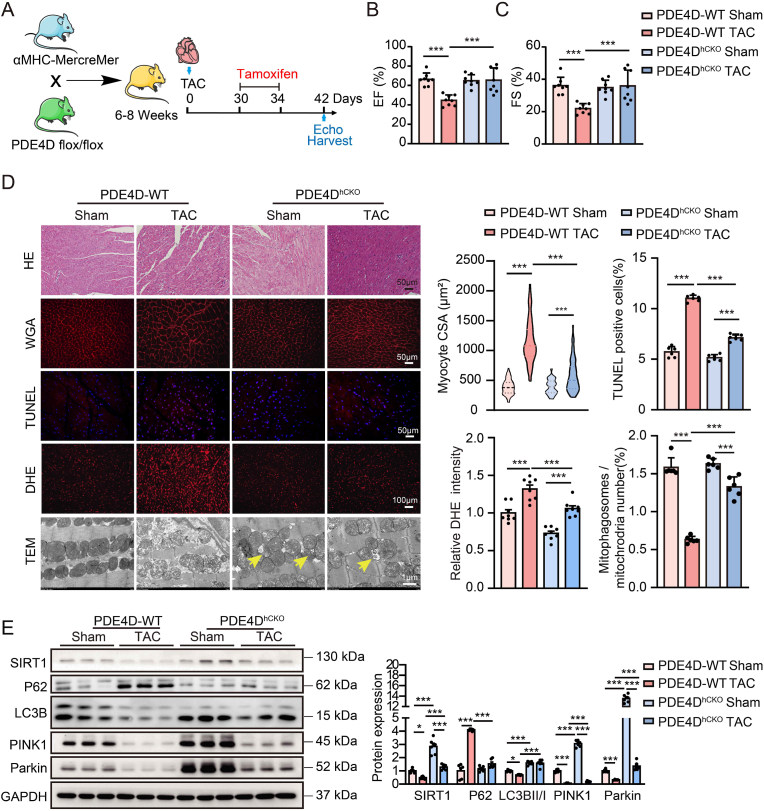
**Cardiac-specific PDE4D-knockdown activates mitophagy and protects against TAC-induced cardiac hypertrophy and heart failure**. **A,** 30 days after TAC or sham surgery, αMHC-Cre+/PDE4D-flox/+ mice were subjected to a single intraperitoneal injection of tamoxifen (100 mg/kg/day) for 5 consecutive days to induce PDE4D ablation in the heart (PDE4D^hCKO^). Littermate PDE4D-WT with the same dose of tamoxifen as controls. The mice were sacrificed 12 days after tamoxifen injection. **B** and **C,** Ejection fraction (**B**) and fractional shortening (**C**) were measured by echocardiography at 6 weeks after TAC; n = 8 mice per group. **D,** Cardiac cross-sections were stained with hematoxylin-eosin or WGA to examine heart morphology and measure myocyte CSA; n = 100 cells per heart and n = 3 mice per group. Scale bar, 50 μm. The myocardial apoptosis was detected by TUNEL staining, 2 random fields per heart; n = 3 mice per group. Scale bar, 50 μm. ROS levels were determined by DHE fluorescence intensity in two random fields per heart; n = 4 mice per group. Scale bar, 100 μm. Mitochondrial morphology was determined using transmission electron microscopy, and the number of mitophagosomes was quantitated. The arrows indicate mitophagosomes; 2 random fields per heart; n = 3 mice per group. Scale bar, 1 μm. **E,** Representative immunoblots showing SIRT1, P62, LC3B, PINK1, and Parkin expression, n = 6 mice per group. All data are presented as mean ± SEM. Two-way ANOVA with Tukey's multiple comparison test was used for **B**, **C** and **E**; nested ANOVA analyses were done for **D**.

Next, tamoxifen-inducible cardiac-specific heterozygous PDE4D knockout (PDE4D^hCKO^) mice were selected to assess the effects of cardiac PDE4D inhibition in HF. The knockout efficiency and specificity of PDE4D in cardiomyocytes were confirmed by immunofluorescence staining (Figs. S16A and S16B). 30 days post-TAC, mice were treated with tamoxifen for 5 days followed by a 7-day recovery period (Fig. 7A). Increased cardiac PDE4D expression and decreased cAMP levels induced by TAC were reversed in PDE4D^hCKO^ mice (Figs. S17A and S17B). Similar to roflumilast, cardiac-specific haploinsufficiency of PDE4D markedly improved TAC-induced cardiac contractile dysfunction (Fig. 7B and C, Table S4), hypertrophy (Fig. 7D, Figs. S17C and S17D), and reduced apoptosis, ROS and MDA level (Fig. 7D, Fig. S17E). Furthermore, downregulation of cardiac PDE4D significantly ameliorated TAC-reduced myocardial mitochondrial cristae score and mitophagosome formation (Figure S17F, Fig. 7D) and ATP content (Fig. S17G). The decreased expression of SIRT1 induced by TAC was also significantly ameliorated in the PDE4D^hCKO^ mouse hearts (Fig. 7E). The expression of P62 was decreased, and the expression of LC3BII/I, PINK1 and Parkin, and Parkin-mitochondrial colocalization were significantly increased in PDE4D^hCKO^ TAC mouse hearts compared with PDE4D-WT TAC mouse hearts (Fig. 7E and Figure S17H). Collectively, these results suggest that cardiac-specific PDE4D haploinsufficiency plays a protective role against TAC-induced cardiac hypertrophy and HF.

### 3.8 Cardiac overexpression of PDE4D5 counteracts the cardiac protective effects exerted by PDE4D knockout in TAC mice

To further investigate the role of PDE4D5 in pathological cardiac hypertrophy and HF in vivo, we performed an AAV9-mediated rescue experiment using heterozygous PDE4D global knockout mice (PDE4D^+/−^). Immunofluorescence staining was performed to examine the specific overexpression of PDE4D in cardiomyocytes and the knockout efficiency of PDE4D in cardiomyocytes of PDE4D^+/−^ mouse hearts, respectively (Fig. S18). PDE4D^+/−^ mice showed increased cAMP content after TAC compared with control littermates WT TAC mice (S19A and S19B). Similar to cardiac-specific heterozygous PDE4D knockout mice, PDE4D^+/−^ mice prevented TAC-induced cardiac contractile dysfunction (Figs. S19C and S19D, Table S5), hypertrophy (Figure S20A, S20B and S20F), apoptosis, excessive ROS formation and MDA content (Figs. S20C and S20F). Reduced myocardial ATP production and mitophagosome formation following TAC were also improved in PDE4D^+/−^ mice (Figs. S20D and S20F). Cardiac-specific PDE4D5 overexpression via the intravenous injection of an AAV9 vector encoding PDE4D5 under the cardiac troponin T (cTNT) promoter aggravated cardiac hypertrophy and contractile dysfunction in TAC mice (Figure S19, Figure S20A, S20B and S20F). Furthermore, cardiac PDE4D5 overexpression exacerbated TAC-induced apoptosis, increased ROS level and MDA content and reduced mitochondrial cristae score, mitophagosome formation and ATP content (Figure S20C through S20F). Rescue experiments showed that cardiac PDE4D5 overexpression counteracted the protective effects of heterozygous PDE4D mutations (Figure S19, Figure S20A through S20F). We observed that cardiac PDE4D5 overexpression downregulated expression of SIRT1 and mitophagy related protein LC3B, PINK1 and Parkin as well as colocalization of Parkin and Tomm20 in TAC mice (Figs. S21A and S21B). Moreover, global PDE4D deficiency-induced increase in the expression of SIRT1, PINK1, Parkin and LC3BII/I, Parkin-mitochondrial colocalization and decrease in P62 expression were abolished by cardiac PDE4D5 overexpression (Figs. S21A and S21B). Collectively, these results strongly suggest that PDE4D5 plays a critical causative role in promoting cardiac hypertrophy and HF by suppressing mitophagy.

## 4 Discussion

**Fig. 8:**
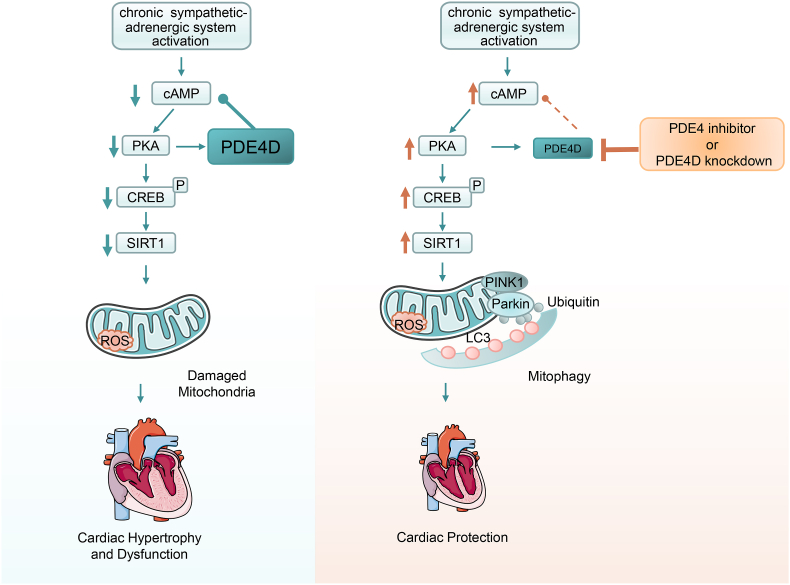
**Schematic of the proposed mechanism for chronic adrenergic stimulation induced heart failure via cAMP-PKA-PDE4D signaling pathway and protective mechanism of PDE4D inhibition**. Sustained adrenergic activation upregulates PDE4D expression via cAMP-PKA signaling, which in turn reduces cAMP-PKA activity, resulting in cardiomyocyte hypertrophy and mitochondrial injury via inhibition of CREB-SIRT1 signaling-mediated mitophagy. Pharmacological inhibition or knockdown of PDE4D reduced cAMP degradation and activates PKA-CREB-SIRT1 signaling, leading to a restoration of PINK1/Parkin-mediated mitophagy in cardiomyocytes, which may contribute to improvement of cardiac function and hypertrophy.

In the present study, we report that PDE4D expression is increased in failing human and mouse hearts. Using various pharmacological and genetic gain- or loss-of-function approaches, we demonstrated that PDE4D haploinsufficiency or pharmacological inhibition can improve cardiomyocyte hypertrophy and HF by promoting PINK1/Parkin-mediated cardiac mitophagy via CREB-SIRT1 signaling pathway (Fig. 8).

Oxidative stress is a principal causative factor of mitochondrial damage, which contributes to the progression of hypertrophy and HF [20]. Mitophagy is important in eliminating impaired mitochondria and is required for cardiac homeostasis maintenance and stress adaptation [20]. The majority of literature suggests that restoration of mitochondrial autophagy attenuates cardiac dysfunction in the heart during pressure overload [21]. PINK1 and Parkin signal pathways have been recognized as a major process associated with mitochondrial quality maintenance via mitophagy activation [22]. PINK1/Parkin-dependent mitophagy activation accelerated impaired mitochondria clearance and prevented HF progression [23]. Our data showed that TAC mice developed cardiac hypertrophy and HF, and these changes coincided with decreased PINK1/Parkin-mediated mitophagy levels. We found that PDE4D inhibition protects the heart by promoting PINK1/Parkin-induced mitophagy, which is consistent with other studies that PDE4 inhibition protects neurons against oxidative stress via autophagy [24].

SIRT1 activation can improve aging-induced cardiomyocyte contractile dysfunction and mitophagy loss [25] and repair the infarcted heart via Parkin-dependent mitophagy [26]. Our results suggest that PDE4D inhibition promotes mitophagy via SIRT1 signaling to protect cardiomyocytes from pathological hypertrophic stimuli, which is in line with previous reports that SIRT1 is likely to be an adaptive mechanism of cardiomyocytes against HF [27], despite some investigations demonstrating that SIRT1 is involved in HF progression by promoting mitochondrial dysfunction [28]. These contradicting findings could possibly be related to the increased extent of SIRT1 activation [28,29]. It has been shown that moderate SIRT1 overexpression protected the heart from oxidative stress through the increased expression of antioxidants, whereas higher SIRT1 levels induce cardiomyopathy, possibly through the induction of mitochondrial dysfunction [29]. This is unsurprising, given that mitophagy has been considered a double-edged sword in the context of cardiovascular disease since excessive mitophagy can negatively impact the mitochondrial energy metabolism in cardiomyocytes. Given that PDE4D can regulate SIRT1 expression in cardiomyocytes [10]. This may explain why the findings of our study of heterozygous PDE4D knockout mice were inconsistent with those of the literature that complete knockout of PDE4D fosters HF development following myocardial infarction [8].

In addition, the control of cAMP-regulated hypertrophy by PDEs is an intricate process in HF. Different cAMP levels may have opposing effects on myocyte contractility, growth, and death [13]. The rise in cAMP generated via inhibition of PDE3 or PDE4 has pro-hypertrophic effects [30], while enhancement of particular aspects of cAMP/PKA signaling appears beneficial for failing hearts [31]. It has been reported the antihypertrophic effect of PDE2 inhibition relies on a local increase in cAMP that enhances PKA-mediated phosphorylation of nuclear factor of activated T cells [30]. Based on these observations, different cAMP pools and down-stream PKA signaling have opposing effects on cardiac myocyte cell size [30].

PKA is upstream of PDE4 and activation of the long PDE4 isoforms (PDE4D3 and PDE4D5) occurs by PKA-mediated phosphorylation [32]. In line with this, our results showed that chronic ISO stimulation promoted PDE4D expression via PKA activation, but has little effect on PDE4B expression. These results were consistent with previous study that PDE4B was not activated by PKA in response to ISO treatment, whereas PDE4D were activated in mouse embryonic fibroblasts [19]. In addition, ablation of PDE4D, but not PDE4B, had a major effect on the ISO-induced PDE4 activation [19]. Similar to a previous study showing that PDE4D is phosphorylated and activated by PKA upon prolonged β-AR stimulation and serves as a negative mechanism to keep cAMP levels [4], we found that PDE4 inhibitor roflumilast returned cAMP content and the expression of PDE4D to normal levels in ISO-treated mouse hearts, suggesting that PDE4D upregulation is responsible for the reduced cAMP-PKA activity under chronic ISO stimulation. Notably, overexpression of PDE4D per se is sufficient to induce cardiac myocyte hypertrophic growth and oxidative stress. In contrast, PDE4B overexpression protected against from ISO-induced hypertrophy and ROS level in cardiomyocytes, which is in agreement with other animal studies [11,12].

The reason for these apparently opposite effects of PDE4B and PDE4D may partially be related to their distinct subcellular localization since CREB revealed some colocalization with PDE4D rather than PDE4B. SIRT1 is a direct transcriptional target of CREB, which is activated by cAMP-PKA [10]. PDE4 inhibitor roflumilast significantly restored CREB activity in ISO-treated cardiomyocytes. Moreover, PDE4D overexpression reduced the phosphorylation of CREB, whereas PDE4B overexpression did not affect CREB phosphorylation. Some other research groups also observed similar phenomenon that inactivation of PDE4D but not PDE4B significantly altered the level of CREB phosphorylation, which was considered the altered cAMP accumulation caused by PDE4D ablation is reflected in changes of downstream effectors [19]. We speculate that the different effects of PDE4B and PDE4D on CREB-SIRT1 signaling pathway and mitophagy might be partially responsible for their distinctive roles in cardiomyocytes.

Previous studies reported HF leads downregulation of PDE4B and PDE4D in C57BL/6 N mice [12] and PDE4D is decreased in human failing hearts [8]. Here, we found that the expression of PDE4D is increased in failing human and mouse hearts. Database from GEO datasets (GSE120895 and GSE95140) also showed upregulation of PDE4D mRNA in HF human samples. These inconsistent results may be related to several reasons. It is known that splice variants of PDE4D are differentially expressed and regulated [33] and PDE4D expression level is affected by multiple factors including gender, age, and etiology, etc. [13]. In the study reporting decreased PDE4D in human failing hearts [8], PDE4D refers to the PDE4D3 in the ryanodine-receptor complex but not PDE4D in whole cell. The latest research showed that region-dependent differences in cAMP activity coincide with PDE4 activity in mouse hearts [34]. It was reported that mitophagy was transiently activated in the early phase but downregulated in the chronic phase of TAC. Similarly, the PDE4D expression was relatively constant at 2 weeks and started to increase at 8 weeks in the mice subjected to TAC (GSE182895). Moreover, the PKA activity reached a peak 2 days post-TAC and then decrease which could be due to desensitization of the β-adrenergic system [5]. These data indicate that the expression levels and activities of PDE4 undergoes dynamic changes in different stages of HF. In addition, it is common that gene expression changes could be etiology specific. In patients with different HF types, cardiac PDE4D expression was increased in hypertrophic cardiomyopathy and peripartum cardiomyopathy but decreased in familial dilated cardiomyopathy compared to non-diseased donors (GEO Accession GSE2656).

Roflumilast is a selective PDE4 inhibitor, which inhibits all the PDE4 subtypes to a similar extent. In this study, roflumilast reversed the effects of ISO and TAC on PDE4D expression but not on PDE4B expression. The specific responses of PDE4B and PDE4D to treatment of roflumilast are consistent with our recent studies that roflumilast treatment attenuated PDE4D induction and had no effect on the expression level of other PDE4 subtypes in HFD mouse hearts [10]. The elevated PDE4 activity in ISO-injection and TAC mice might be due to increased PDE4D and the reason why roflumilast showed no obvious inhibit effects on PDE4B expression might be related with its relative low expression compared to PDE4D expression.

Roflumilast blocks enzyme activity of PDE4 via binding to active sites of PDE4. However, roflumilast also inhibits the mRNA and protein expression of PDE4D in different disease models, which may be attributed to the complexity of the regulation of PDE4 activity and expression in cells. For example, expression of PDE4B and PDE4D were both increased in the lungs by lipopolysaccharide (LPS), PDE4-inhibitors rolipram and roflumilast decreased mainly PDE4B [35], while roflumilast reversed the increased expression of PDE4B and PDE4D in the cerebral cortex and hippocampus of Alzheimer's Disease mice [36]. It is known cAMP levels are regulated by PDE4 but cAMP also control the expression of PDE4 in cells and PDE4D can be activated by PKA [[37], [38], [39]], reflecting a system of feedback between cAMP-PKA signaling and PDE4. A prolonged elevation of cAMP in vascular smooth muscle cells resulted in a PKA-dependent induction of PDE4D expression, simultaneous activation of both the cAMP-PKA and PKC-Raf-MEK-ERK signaling cascades blunted this cAMP-mediated increase in PDE4D expression, which is mediated by a mechanism involving PDE4D mRNA stability [38]. It seems contradictory that ISO-activated cAMP signaling and roflumilast-activated cAMP signaling exhibit conversely regulatory effects on PDE4D expression. Previous study showed that PDE4 inhibitor rolipram alone did not affect cAMP levels in cardiomyocytes under basal state but promoted a higher and sustained cAMP signal after acute stimulation with ISO. The addition of rolipram after ISO-induced first transient peak recovered a second strong and sustained cAMP signal in cardiac myocytes [4]. Similarly, our study showed that roflumilast alone did not affect the expression level of PDE4D in basal state. Prolonged activation of cAMP by ISO upregulated PDE4D expression, which subsequently degraded cAMP. Under this condition, PDE4D levels exceeded basal levels and the cAMP level decreased below basal state, treatment with roflumilast suppressed PDE4D activity, thereby restoring cAMP level followed by PDE4D downregulation via an unknown signaling pathway. We speculated that PDE4D expression were enhanced or lowered during activation of cAMP by ISO or PDE4D inhibition, depending on the basal level of PDE4D and cAMP.

As β-blocker treatment has emerged as an effective treatment modality for heart failure, there is a general view that PDE4 protects the heart against sustained adrenoreceptor stimulation since βARs activation induces cardiac hypertrophy and apoptosis via the cAMP-PKA signaling activation [13]. Given that there is a feedback loop (cAMP → PKA → PDE4 → cAMP↓) which is critical for the maintenance of normal cellular homeostasis [39], we speculated that during chronic β-AR overstimulation the dynamic changes of cAMP level increased first and then decreased due to upregulation of PDE4D, and chronic β-AR overstimulation induced cardiomyocyte cell injury via cAMP-PKA-PDE4D signaling pathway. Therefore, reduction in β-AR function can be cardio-protective might due in part to inhibition of PDE4D induction, which does not contradict our current study that inhibition of PDE4D has protective effects. Previous study supported this view. Hyperinsulinemia-induced PDE4D expression via β_2_AR impaired adrenergic regulation in a diabetic model. β-blocker carvedilol attenuated insulin-induced PDE4D induction and restored cAMP level to prevent diabetes-associated cardiac dysfunction [9]. Therefore, PDE4D is a potential therapeutic target for heart failure treatment, but its intervention strategies require optimization based on precise regulation of cAMP signaling. Detailed mechanisms of this exquisite regulation await further investigation.

Our study has following limitations. A latest study reported the sex-dependent differences in PDE activity in the mouse heart [34]. Female hearts had higher expression of PDE4D compared to male hearts; and the apex of female hearts had higher total PDE activity, which may contribute to faster cAMP breakdown in this region. PDE inhibition prevented repolarization changes in female hearts [34]. There is the possibility that female mice are more sensitive than males to the cardio protective effects of PDE4 inhibitor treatment. It will be essential to investigate the potential role of sex differences in cAMP signaling and PDE activity in pressure overload model to assess the anti-hypertrophy effects of PDE4 inhibitor in different gender group. Further exploration is needed to pinpoint the exact cause of chronic adrenergic stimulation induced upregulation of PDE4D but not PDE4B. The different downstream targets of cAMP/PKA signaling that are modulated following PDE4B and PDE4D overexpression should be analyzed by a proteomic approach. The molecular mechanisms underlying the different localization of PDE4B and PDE4D with CREB should be elucidated. PDE4 is known to correlate with proinflammatory activity in several autoimmune diseases and PDE4 inhibitors may affect both cardiomyocytes and other cell types in HF in vivo. PDE4B inhibition prevented HF in myocardial ischemia-reperfusion mice by attenuating neutrophil inflammation [40]. Notably, PDE4D5 is the lowest expressing isoform in human hearts in contrast to rodents [7], which may cause the distinct pharmacological effects of PDE4 inhibition in rodent and human hearts. Thus, further studies are needed to clarify whether PDE4 inhibition provides therapeutic effects in HF of various etiologies, and clinical pilot human studies are needed to evaluate the therapeutic potential of PDE4D inhibition.

In conclusion, our results highlight a novel mechanism by which sustained adrenergic activation upregulated PDE4D expression via cAMP-PKA signaling, which in turn reduces cAMP-PKA activity, resulting in cardiomyocyte hypertrophy and mitochondrial injury via inhibition of CREB-SIRT1 signaling-mediated mitophagy. PDE4B overexpression does not affect CREB-SIRT1 signaling and mitophagy but protects against ISO-induced cardiomyocyte injury. The therapeutic significance of our findings is that PDE4D is a druggable target for the treatment of HF. The design of PDE4D isoform selective inhibitors may help improve therapeutic efficacy for HF and might be an effective way to reduce cardiovascular risk in patients who are being treated with PDE4 inhibitors.

## CRediT authorship contribution statement

**Jing Fu:** Writing – review & editing, Writing – original draft, Investigation. **Congping Su:** Writing – review & editing, Writing – original draft, Investigation. **Yin Ge:** Investigation. **Zhou Ao:** Writing – review & editing. **Li Xia:** Investigation. **Yingxiang Chen:** Methodology. **Yizheng Yang:** Methodology. **Shiwei Chen:** Methodology. **Rui Xu:** Supervision. **Xiaoyan Yang:** Supervision. **Kai Huang:** Supervision. **Qin Fu:** Writing – review & editing, Writing – original draft, Supervision, Project administration, Funding acquisition, Conceptualization.

## Funding

This work was supported by 10.13039/501100001809National Natural Science Foundation of China grants 81773730 and 82273926 to Q.F and 82270369 to X.Y, Wuhan Natural Science Foundation Exploration Plan (2024020801020393) and Hubei Provincial Natural Science Foundation of China 2025AFB640 to J.F.

## Declaration of competing interest

The authors declare that they have no known competing financial interests or personal relationships that could have appeared to influence the work reported in this paper.

## Data Availability

Data will be made available on request.
