# Supplementary material for: PDE4D inhibition ameliorates cardiac hypertrophy and heart failure by activating mitophagy
Source: Redox Biol. 2025 Feb 22;81:103563. doi: 10.1016/j.redox.2025.103563 (PMC11909752; doi:10.1016/j.redox.2025.103563)
Supplement: Multimedia component 1 [file mmc1.docx]

Supplementary Materials

**PDE4D inhibition ameliorates cardiac hypertrophy and heart failure by activating mitophagy**

Jing Fu^1,2^*; Congping Su ^1^*; Yin Ge^1^; Zhou Ao^1^; Li Xia^1^; Yingxiang Chen^1^; Yizheng Yang^1^; Shiwei Chen^1^; Rui Xu^1^; Xiaoyan Yang^1, 3^; Kai Huang^4^; Qin Fu^1, 3^

* J. Fu and C. Su are contributed equally to this work.

**Address for Correspondence:**

Qin Fu, PhD,

Department of Pharmacology, School of Basic Medicine

Tongji Medical College, Huazhong University of Science and Technology

Hangkong Road 13^th^, Wuhan, China, 430030

Email: [fuqin@mails.tjmu.edu.cn](mailto:fuqin@mails.tjmu.edu.cn)

**This file includes:**

Supplementary Methods

Supplementary Figure. 1 to Supplementary Figure. 21

Supplementary Table 1 to Supplementary Table 8

1. **Supplemental Methods**
   1. **Echocardiography**

Echocardiography was performed with an ultrasound transducer, as described previously.^10^ Briefly, mice were anesthetized with 1.5% isoflurane supplemented with oxygen and subjected to transthoracic echocardiography (Visual Sonics Vevo 2100 Imaging System, Toronto, Canada) to assess cardiac structure and function. To minimize variation in the data, the body temperature was controlled and the heart rate was maintained at 350-450 beats per minute during cardiac function measurements. Systolic function parameters, including the ejection fraction and fractional shortening, were measured using the two-dimensional parasternal short-axis imaging plane of the M-mode traces. The measurements were conducted by an investigator blinded to the treatment and genotype.

**1.2 Cardiac mitochondria isolation**

Mitochondria were extracted and purified from fresh mouse heart tissue using a mitochondria Isolation Kit (Beyotime, Nanjing, China). Briefly, heart tissues were homogenized in mitochondrial lysis buffer and then centrifuged 1000 *g* at 4 °C for 5 min. The pellets were discarded, and the supernatant was collected and centrifuged at 3500 *g* at 4 °C for 10 min. The pellets were then retained as mitochondria extract and the supernatant was centrifuged at 12000 *g* at 4 °C for 10 min and collected as a cytoplasmic extract.

**1.3 Histology**

Mice were euthanized and their hearts were arrested with a 10% potassium chloride solution at end-diastole and then fixed in 4% paraformaldehyde. Fixed hearts were embedded in paraffin and serially sectioned at 5 μm on a microtome. Serial heart sections were stained with hematoxylin-eosin or wheat germ agglutinin (WGA) (Invitrogen, Carlsbad, CA, USA) to examine heart morphology and measure myocyte cross-sectional areas. Myocardial apoptosis was detected using a TUNEL Apoptosis Assay Kit (Beyotime, Nanjing, China). For immunofluorescence staining, tissue sections were incubated in a blocking buffer containing 5% goat serum, 0.1% Triton X-100, and polybutylene succinate (PBS) for 1 h at room temperature, followed by incubation with primary antibody overnight at 4 °C. The sections were washed with PBS and incubated with secondary antibodies for 1 h at room temperature. Fluoroshield mounting medium with 4′,6-diamidino-2-phenylin-dole (DAPI) (Servicebio, Wuhan, China) was used to cover the slides, and images were captured using a microscope. Histological quantification was performed using the ImageJ software (National Institutes of Health, Bethesda, MD, USA).

**1.4 Transmission electron microscopy (TEM)**

The separated fresh left ventricular tissue was immediately cut into small pieces (1 mm^3^) and fixed with EM fixation buffer (Servicebio, Wuhan, China). Samples were rinsed repeatedly in the same buffer and post-fixed in 1% osmium tetroxide in a 0.1 M sodium cacodylate buffer (pH 7.4) for 2 h to produce osmium black. The samples were then dehydrated in a graded ethanol series and embedded in Epon812 (SPI, USA). Ultrathin sections of the mouse heart (70 nm thick) were cut, stained with osmium tetroxide/uranyl acetate and lead citrate, and photographed using a transmission electron microscope (Hitachi HT-7800, Tokyo, Japan) at 8000× and 15000× direct magnification. The mitochondrial morphology was categorized according to a modified 5-grade scoring system^41,42^. score 1: classical healthy mitochondria with well-defined cristae; score 2: initial stage of swollen mitochondria (occasional swollen cristae, slightly irregular); score 3: megamitochondria (major distortions, high degree of cristae disorganization and swelling, and discontinuous membrane and cristae); score 4: massive, swollen matrix mitochondria (membranes and cristae dissociated into particulates to produce diffuse mitochondrial ghosts); score 5: vacuolization (delamination of inner and outer mitochondrial membrane, absent cristae, vacuolization).

**1.5 Measurement of reactive oxygen species (ROS) in cardiac tissues**

Fresh heart tissues were embedded in an optimum cutting temperature compound (OCT, Sakura, CA, USA) and immediately frozen in liquid nitrogen. Tissues were sectioned at 10 µm thickness on a cryostat, mounted on glass slides, and then incubated with 5 µmol/L dihydroethidium (DHE) (Beyotime, Nanjing, China) at 37 °C for 30 min while shielded from light. After washing with PBS, images were captured using a fluorescence microscope (BX53; Olympus, Japan). ImageJ was used to quantify the DHE fluorescence intensity in the cardiac sections.

**1.6 Detection of PDE4 activity, cyclic AMP, and ATP and MDA content**

According to manufacturer instructions: PDE4-specific enzymatic activity in heart tissues was assayed using a mouse PDE4 Elisa Kit (Jianglai Biotech, Shanghai, China), cAMP levels in heart tissues were measured using the cAMP-Glo™ Assay kit (Promega, Madison, WI, USA), ATP levels and MDA content in the heart tissues were measured using ATP and MDA Assay Kit, respectively (Beyotime, Nanjing, China).

**1.7 Immunocytochemistry**

Neonatal rat left ventricular myocytes (NRVMs) were seeded on coverslips and after corresponding treatment, washed three times with phosphate-buffered saline (PBS, pH 7.4), fixed in 4% paraformaldehyde for 20 min at room temperature, and then permeabilized with 0.3% Triton X-100 for 5 min. After blocking with 5% bovine serum albumin for 30 min, the cells were incubated with primary antibodies overnight at 4 °C. The cells were washed with PBS and incubated with fluorochrome-conjugated secondary antibodies for 1 h at room temperature. Nuclei were counterstained with DAPI (Beyotime, Nanjing, China) and observed using a laser-scanning confocal microscope (Olympus, FV3000, Japan).

1.8 Proximity Ligation Assay (PLA)

*In situ* PLAs were performed according to the manufacturer's procedure to detect endogenous

protein-protein interactions using the Duolink®kit (Sigma-Aldrich). This protocol allows for the detection of two target proteins that are in close proximity. Briefly, NRVMs were fixed, permeabilized, and blocked. Cells were then incubated with primary antibodies against Tomm20-PINK1, Tomm20-Parkin, Tomm20-LC3B, and Tomm20-IgG at 1:200 dilution overnight at 4°C, respectively. PLA probes without primary antibodies served as a negative control. After three washes, cells were incubated with PLA probes, secondary antibodies conjugated to oligonucleotides, for 60 min at 37 °C. Circularization and ligation of appropriate oligonucleotides were performed in ligase-containing solution for 30 min at 37 °C, followed by incubation with amplification solution for 100 min at 37°C. After mounting, the slides were analyzed using confocal microscopy to detect protein-to-protein interaction. Fluorescent puncta representing protein complexes in PLA which indicates two proteins within cells are separated by <40 nm. Quantification of detected PLA puncta per cell was then analyzed using Image J software.

**1.9 Colocalization analysis**

In MitoTracker colocalization assay, the cells were incubated with MitoTracker Red (Beyotime, Nanjing, China) for 30 min and rinsed twice with PBS. Cells were fixed, permeabilized, and blocked for subsequent immunofluorescence experiments. For other experiments the sections were incubated with primary antibodies overnight at 4 °C and Alexa Fluor-conjugated secondary antibodies for 2 h at room temperature. The sections were mounted with DAPI (Beyotime, Nanjing, China), detected using a confocal microscope (Olympus, FV3000, Japan). Colocalization was determined using the Pearson coefficient (https://imagej.net/imaging/colocalization-analysis, ImageJ software).

**1.10 Seahorse XF flux assay**

The Mito Stress Test Kit (Agilent Technology, CA, USA) was used to evaluate the mitochondrial activity and to measure the oxygen consumption rate (OCR). Cells were seeded into a 24-well Seahorse XFp Cell Culture Miniplate and subjected to different treatments. The culture medium was replaced with Seahorse XF RPMI medium supplemented with 10 mM glucose, 2 mM glutamine, 1 mM pyruvate. Cells were incubated for 1 h at 37 °C without CO_2_. The following compounds were injected in a sequential order: 2 μM oligomycin, 2 μM FCCP, and 1 μM rotenone. The data were analyzed by Seahorse Analytics software and normalized on the amount of total cell number/well.

**1.11 Measurement of mitochondrial membrane potential (MMP)**

MMP changes were measured using a JC-1 assay kit (Beyotime, Nanjing, China). Briefly, NRVMs were harvested after treatment and washed twice with cold PBS. Thereafter, the cells were resuspended in a mixture of 500 μl culture medium and 500 µl JC-1 staining fluid for 30 min protected from light at 37 °C. Subsequently, the cells were washed thrice with cold staining buffer prior to flow cytometry (BD Bioscience, USA). MMP was indicated by the ratio of red to green fluorescence intensity. Compensation and analysis were performed using FlowJo version V10.2.

**1.12 Measurement of ROS in NRVMs and mitochondria**

NRVMs were harvested after treatment and incubated with 20 μM DCFH-DA (Beyotime, Shanghai, China) for 30 min or 5 µM MitoSOX™ Red (Invitrogen, CA, USA) for 10 min in the dark at 37 °C. NRVMs were resuspended and washed thrice with PBS. A flow cytometer (BD Biosciences, USA) was used to measure the fluorescence emission and excitation at 490 and 520 nm (DCFH-DA) and 510 and 580 nm (MitoSOX™), respectively. Intracellular and mitochondrial ROS fluorescence signals were visualized using a fluorescence microscope (Olympus, Japan). Fluorescence intensities normalized with the area was measured using FlowJo version V10.2. and Image J, the intensity obtained after subtraction of the background intensity was used for the comparison.

**1.13 Real-time polymerase chain reaction**

Total RNA was isolated from heart tissues and cultured NRVMs using RNAiso Plus (Takara Bio Inc., Kusatsu, Shiga, Japan) and cDNA was reverse transcribed according to standard procedures using the PrimeScript RT Master Kit (Takara Bio Inc.). Real-time RT-PCR was performed using a Step One Plus Real-Time PCR system thermocycler (Thermo Fisher Scientific, Waltham, MA, USA) with TB Green Premix Ex Taq (Tli RNase H Plus; Takara Bio Inc.). The relative gene expressions were calculated using the 2^−ΔΔCt^ method. All primer and siRNA target sequences used in this study are listed in Table S6.

**1.14 Western blot and immunoprecipitation**

RIPA lysis buffer was used to lyse heart tissues, NRVMs, mitochondria and cytoplasm. Protein concentrations were measured using a protein assay kit (Beyotime, Nanjing, China) and quantified using western blotting. Lysates (20-30 μg total protein) were resolved by SDS-PAGE and then transferred onto a PVDF membrane (Merck Millipore, Billerica, MA); membranes were then probed with specific primary antibodies. Chemiluminescent detection was performed using a horseradish peroxidase–coupled secondary antibody and Super Signal West Femto Reagent (Servicebio, Wuhan, China).

Immunoprecipitation (IP) assays were performed as described previously. Briefly, heart lysates were incubated with antibodies covalently linked to protein A/G agarose beads (Pierce) for 4 h at 4 ˚C with shaking. Then, the beads with immunoprecipitants were washed four times with lysis buffer, boiled twice in 1× SDS buffer for 5 min and subjected to western blot analysis.

The primary and secondary antibodies used for WB, IP and IF are listed in Table S7. Commercial assay kits used are listed in Table S8.

**References**

[41] L. Jin, L. Geng, L. Ying, et al., FGF21-Sirtuin 3 Axis Confers the Protective Effects of Exercise Against Diabetic Cardiomyopathy by Governing Mitochondrial Integrity, Circulation 146(20) (2022) 1537-1557.https://doi.org/10.1161/CIRCULATIONAHA.122.059631

[42] C.C. Hsieh, C.Y. Li, C.H. Hsu, et al., Mitochondrial protection by simvastatin against angiotensin II-mediated heart failure, Br J Pharmacol 176(19) (2019) 3791-3804.https://doi.org/10.1111/bph.14781

1. **Supplementary figures**


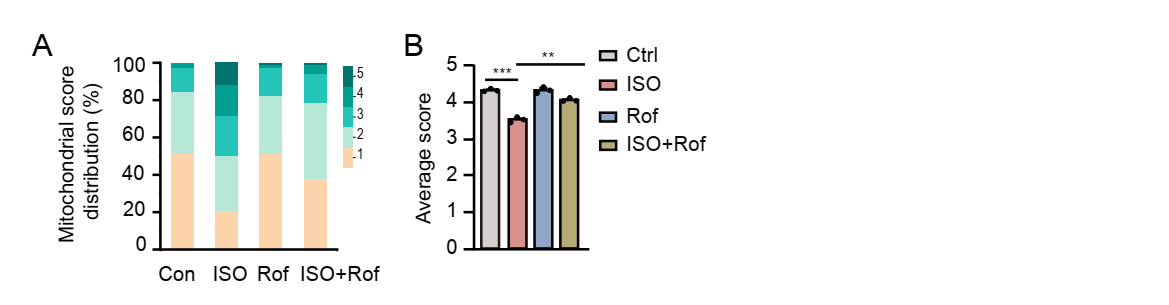


**Supplemental Figure 1. PDE4 inhibitor roflumilast improves mitochondrial morphology in chronic ISO injection treated mouse hearts.**

Relative distribution (A) and average score (B) of mitochondrial cristae score in ISO-treated mouse hearts determined by transmission electron microscopy. Assessment of mitochondrial morphology using a modified 5-grade scoring system. score 1: classical healthy mitochondria with well-defined cristae; score 2: initial stage of swollen mitochondria (occasional swollen cristae, slightly irregular); score 3: megamitochondria (major distortions, high degree of cristae disorganization and swelling, and discontinuous membrane and cristae); score 4: massive, swollen matrix mitochondria (membranes and cristae dissociated into particulates to produce diffuse mitochondrial ghosts); score 5: vacuolization (delamination of inner and outer mitochondrial membrane, absent cristae, vacuolization). n=3 mice per group. All data are presented as mean±SEM. Data were analyzed by one-way ANOVA with Tukey's multiple comparison test.

**Supplemental Figure 2. PDE4 inhibitor roflumilast restores cAMP-PKA signaling pathway in chronic ISO injection treated mouse hearts.**

Male, wild-type mice at 8 weeks old were treated with ISO (7.5 mg/kg/day) or vehicle intraperitoneal injection and administered vehicle or roflumilast (1 mg/kg/day) via oral gavage for 4 weeks. **A,** Transcriptional expression of PDE4 isoforms (PDE4A, PDE4B and PDE4D), n=5 mice per group. **B,** Representative immunoblots and quantification of PDE4D**,** PDE4B, total and phosphorylation of phospholamban (PLB) and CREB in heart tissues; n=5 mice per group. **C,** PDE4 activity in heart tissues; n=4 mice per group. **D,** cAMP levels in heart tissues; n=6 mice per group. All data are presented as mean±SEM. Data were analyzed by one-way ANOVA with Tukey's multiple comparison test.


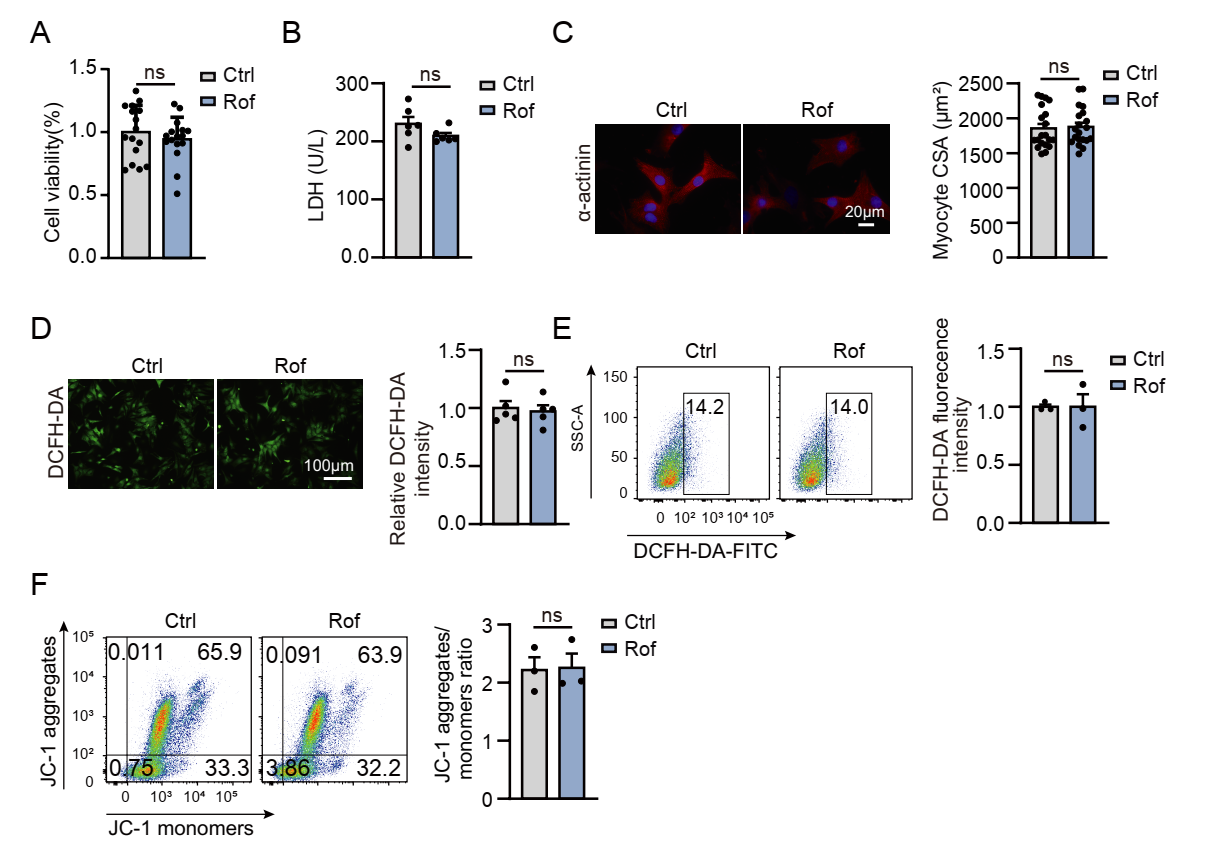
**Supplemental Figure 3. Roflumilast alone has no effect on cardiomyocytes.**

NRVMs were incubated with roflumilast (1 µM) for 24 hours. **A,** Cell viability was assessed by CCK8, n=3 independent experiments. **B,** LDH concentration in the supernatant of NRVMs (n=6 independent experiments). **C,** Immunofluorescence staining of α-actinin (red) and DAPI (blue) with quantification of myocytes area; n=24 cells from 3 independent experiments. Scale bar, 20 µm. **D,** DCFH-DA staining and quantification of ROS levels in NRVMs; n=5 independent experiments. Scale bar, 100 µm. **E,** Quantification of ROS levels in NRVMs with DCFH-DA using FACS; n=3 independent experiments. **F,** FACS analyses of mitochondrial membrane potential (ΔΨm) in NRVMs; n=3 independent experiments. All data are presented as mean±SEM. Unpaired 2-tailed Student t test was used for **B**, **D** through **F**; nested t test analyses were done for **A** and **C**.


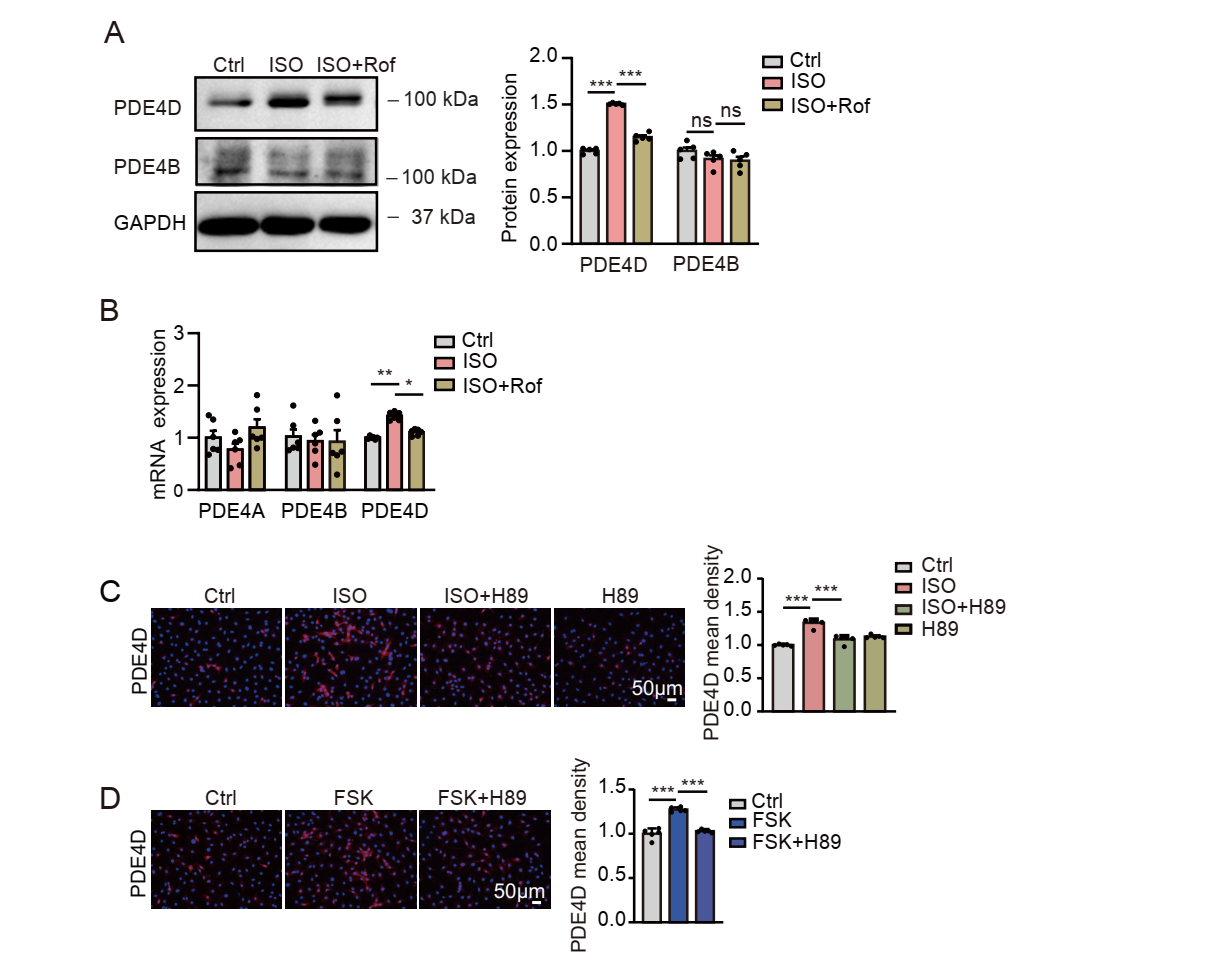


**Supplemental Figure 4. ISO increases PDE4D expression through PKA pathway in cardiomyocytes.**

**A-B,** NRVMs were treated with ISO (10 µM) for 24 hours in the presence or absence of roflumilast (1 µM). **A,** Representative immunoblots and quantification of PDE4D and PDE4B protein expressions in NRVMs; n=5 independent experiments. **B,** qRT-PCR shows PDE4 isoforms mRNA expression in NRVMs of indicated groups. **C,** NRVMs were treated with PKA inhibitor H89 (10 µM) for 30 min followed by vehicle or ISO (10 µM) for 24 hours. **D,** NRVMs were treated with H89 (10 µM) for 30 min and then treated with vehicle or adenylyl cyclase activator forskolin (FSK, 10 µM) for 24 hours. Representative immunofluorescence and quantification of PDE4D (red) with DAPI (blue) in NRVMs; n=4 independent experiments. All data are presented as mean±SEM. Data were analyzed by one-way ANOVA with Tukey's multiple comparison test.


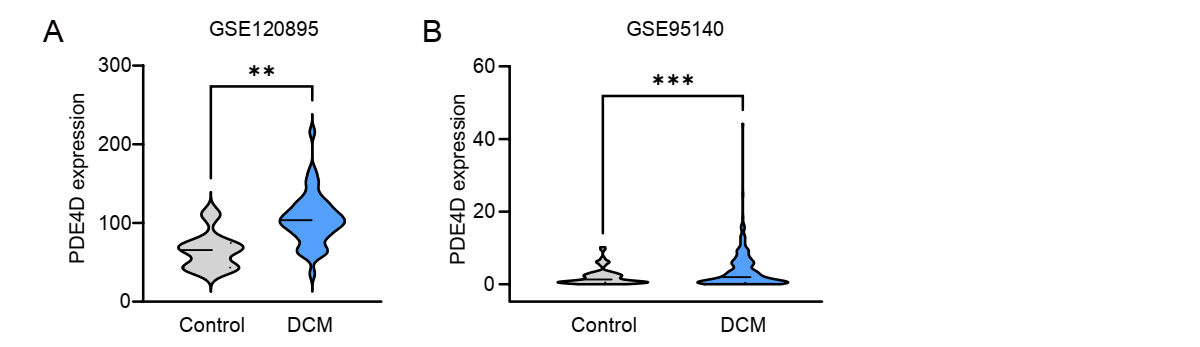


**Supplemental Figure 5. PDE4D expression is up-regulated in dilated cardiomyopathy (DCM) patient hearts and cardiomyocytes.**

Analysis of publicly available microarray data (accession number GSE120895, 95140) in heart samples from DCM patients and control subjects. A. Transcriptional change of the PDE4D gene was quantified in RNAseq (GSE120895, 47 DCM patients and 8 individuals with normal LVEF). B. PDE4D expression analysis in normal control and DCM patients based on single-human cardiomyocyte RNA-sequence (GSE95140). Unpaired t-test with welch’s correction was used for **A** and **B**.


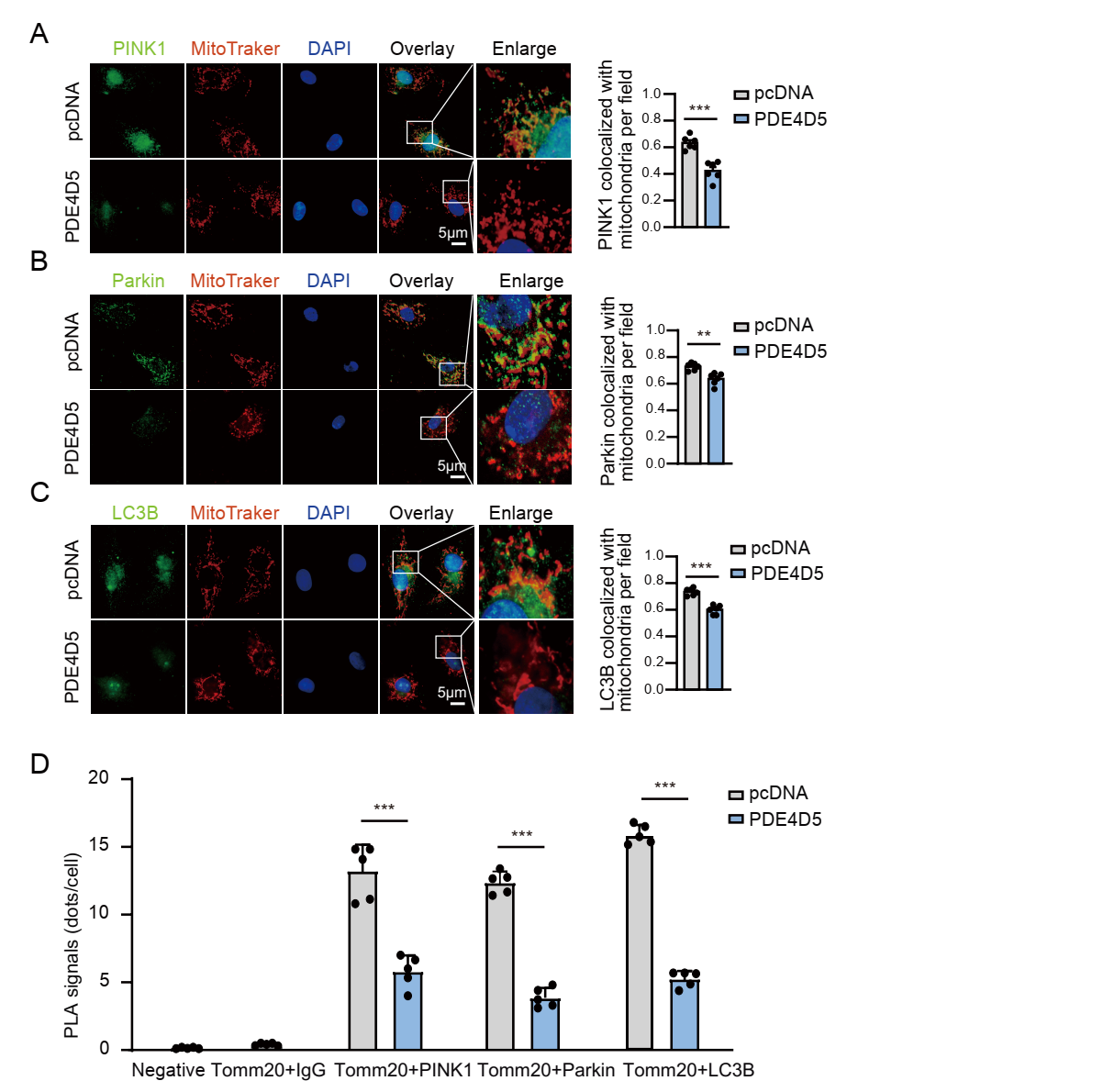
**Supplemental Figure 6. PDE4D5 overexpression reduces the** **colocalization of PINK1, Parkin and LC3B with mitochondria in cardiomyocytes.**

**A** through **C,** NRVMs were transfected with PDE4D5 plasmid or pcDNA control plasmid for 72 hours. Representative immunofluorescence co-staining and Pearson's correlation coefficient for colocalization of MitoTracker (red) with PINK1 (green) (**A**), Parkin (green) (**B**) or LC3B (green) (**C**) in NRVMs, respectively (the merged channels are shown in **Figure 3I**); n=6 independent experiments. Scale bar, 5 µm. **D,** Quantification of PLA signals for the interaction of Tomm20-PINK1, Tomm20-Parkin, or Tomm20-LC3B in NRVMs transfected with PDE4D5 plasmid or pcDNA control plasmid, respectively. Representative images are shown in **Figure 3J**. n=5 independent experiments.


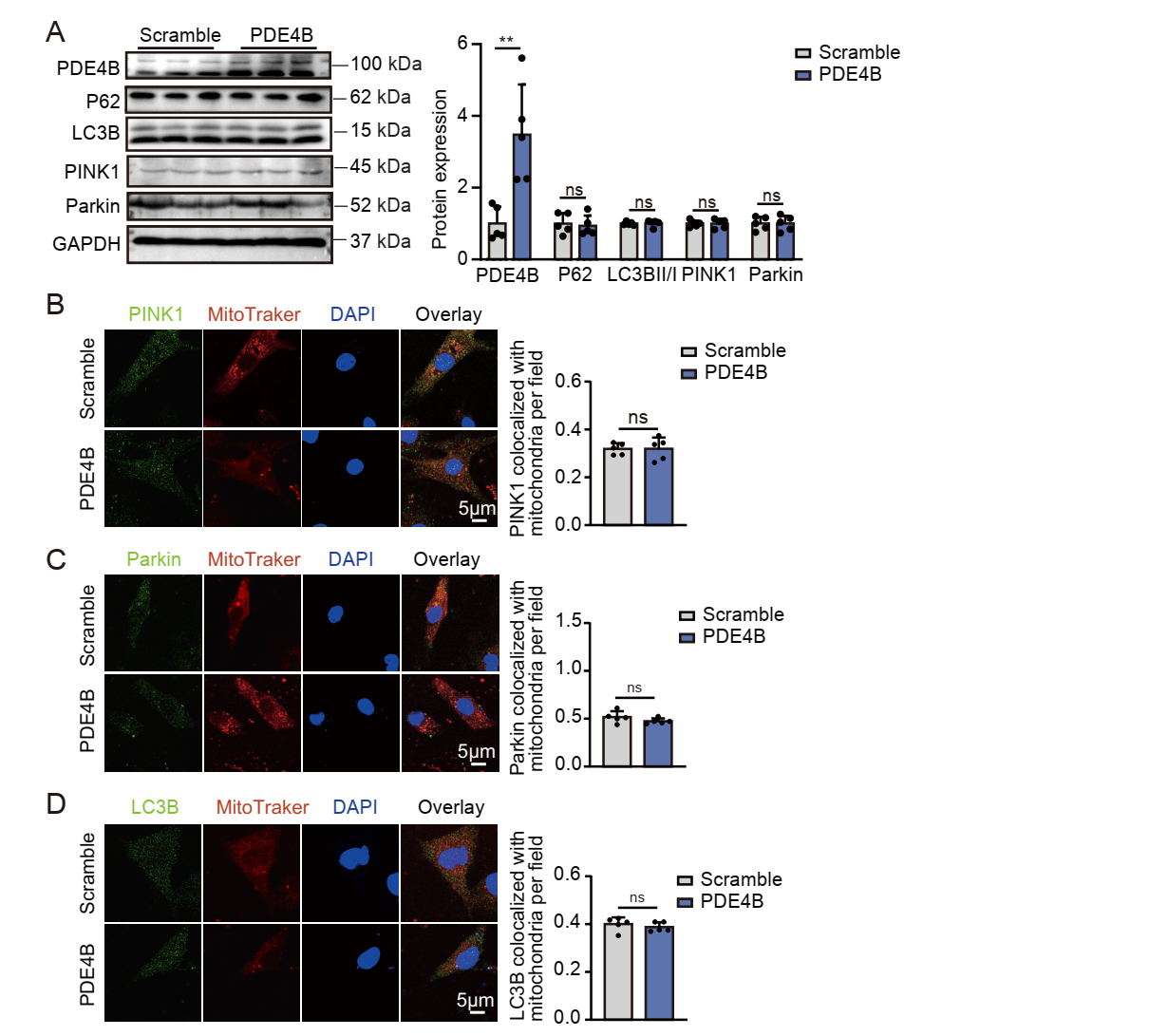


**Supplemental Figure 7. PDE4B overexpression has no effect on mitophagy in NRVMs.**

**A** through **D,** NRVMs were transfected with PDE4B plasmid or control plasmid (Scramble) for 72 hours. **A,** Representative immunoblots and quantification of PDE4B, P62, LC3BII/I, PINK1, and Parkin in NRVMs; n=5 independent experiments. Representative immunofluorescence co-staining and Pearson's correlation coefficient for colocalization of MitoTracker (red) with PINK1 (green) (**B**), Parkin (green) (**C**) or LC3B (green) (D) in NRVMs, respectively; n=5 independent experiments. Scale bar, 5 µm. All data are presented as mean±SEM. Data were analyzed by unpaired 2-tailed Student t test.


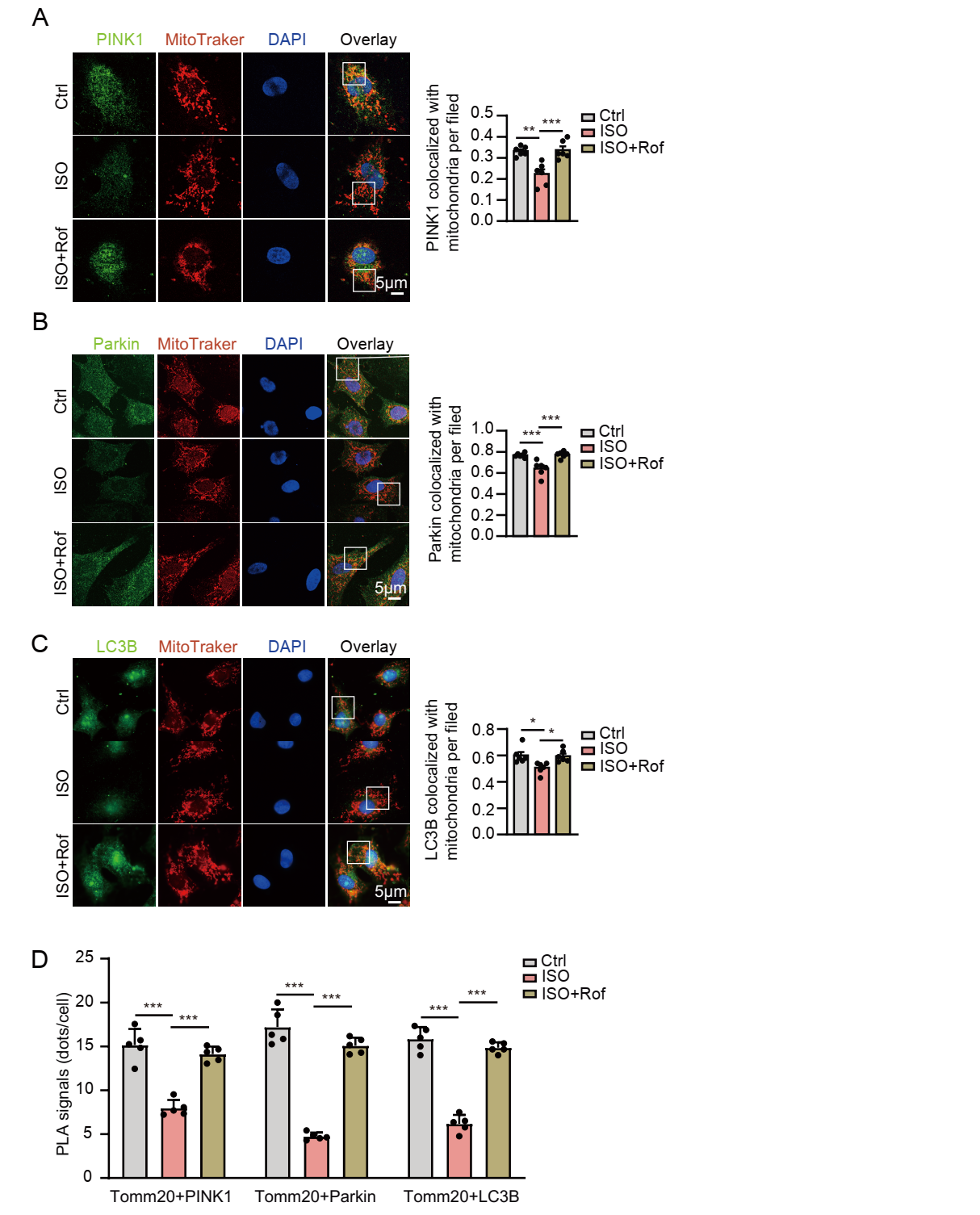


**Supplemental Figure 8. PDE4 inhibitor roflumilast restores** **colocalization of PINK1, Parkin and LC3B with mitochondria in ISO-treated cardiomyocytes.**

**A** through **C,** NRVMs were treated with ISO (10 µM) for 24 hours in the presence or absence of roflumilast (1 µM). Images are single channels from **Figure. 4B** merged images. Representative immunofluorescence co-staining and Pearson's correlation coefficient for colocalization of MitoTracker (red) with PINK1 (green) (**A**), Parkin (green) (**B**) or LC3B (green) (**C**) in NRVMs, respectively; n=6 independent experiments. Scale bar, 5 µm. **D,** Quantification of PLA signals for the interaction of Tomm20-PINK1, Tomm20-Parkin, or Tomm20-LC3B in NRVMs treated with ISO for 24 hours in the presence or absence of roflumilast, respectively. Representative images are shown in **Figure 4C**. n=5 independent experiments.


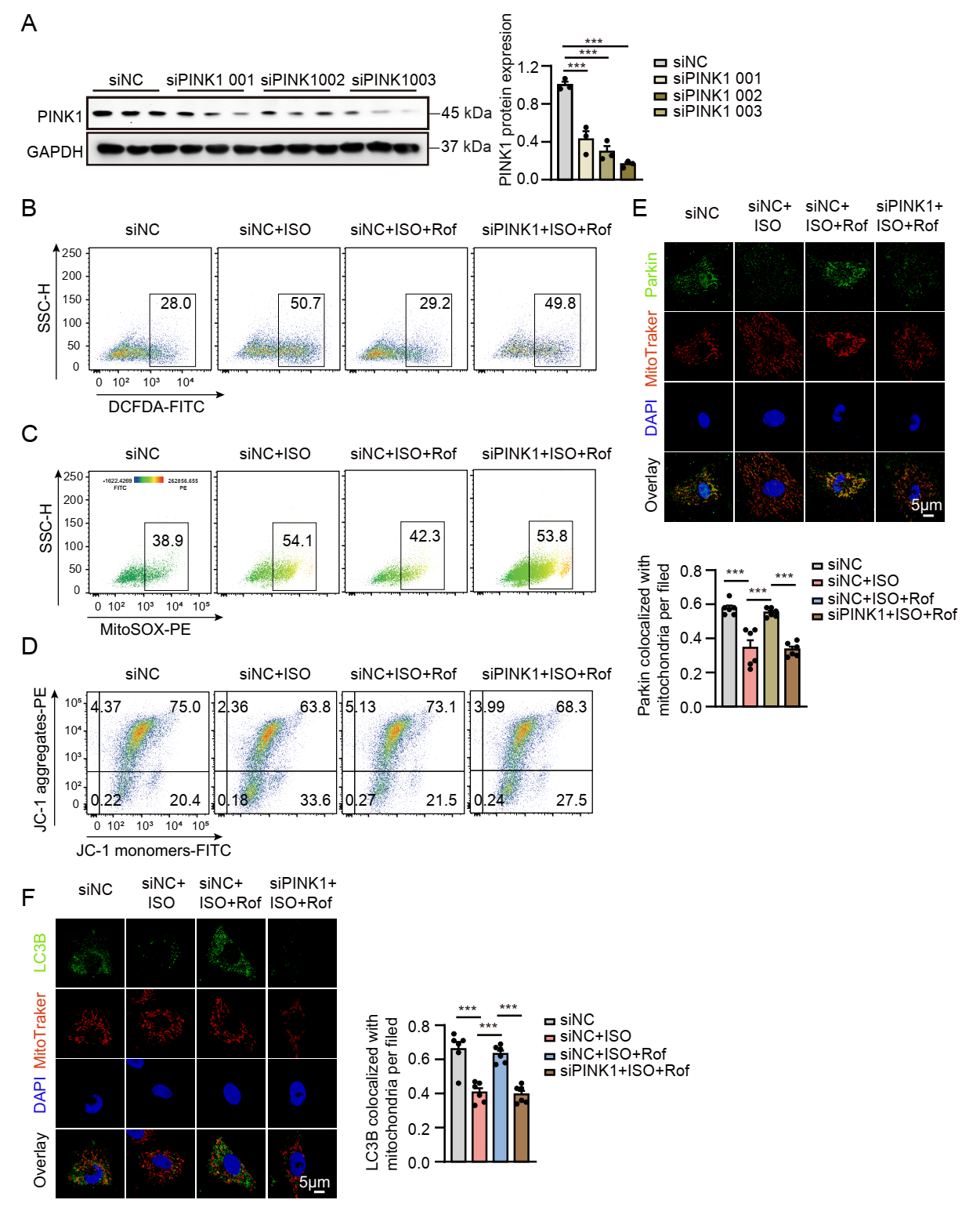


**Supplemental Figure 9. PINK1 knockdown abrogates the protection of PDE4 inhibitor on ISO-induced mitochondrial dysfunction in cardiomyocytes.**

NRVMs were transfected with the indicated siRNAs (siNC or siPINK1) and treated with ISO (10 μM) for 24 hours in the presence or absence of roflumilast (1 μM). **A,** Efficiency of PINK1 siRNA transfection in NRVMs was estimated by detecting the protein expression of PINK1; n=3 independent experiments. **B** through **D,** FACS analyses of cellular ROS levels with DCFH-DA staining (**B**), mitochondrial ROS levels with MitoSOX staining (**C**), and the level of ΔΨm by the ratio of JC-1 aggregates to monomers (**D**), quantifications are shown in **Figure. 4F** through **4H**. **E** and **F,** Representative immunofluorescence co-staining and Pearson's correlation coefficient for colocalization of MitoTracker (red) with Parkin (green) (**E**) or LC3B (green) (**F**) in NRVMs and quantification; 2 random fields from 3 independent experiments. Scale bar, 5 µm. All data are presented as mean±SEM. One-way ANOVA with Tukey's multiple comparison test was used for **A**; nested ANOVA analyses were done for **E** and **F**.


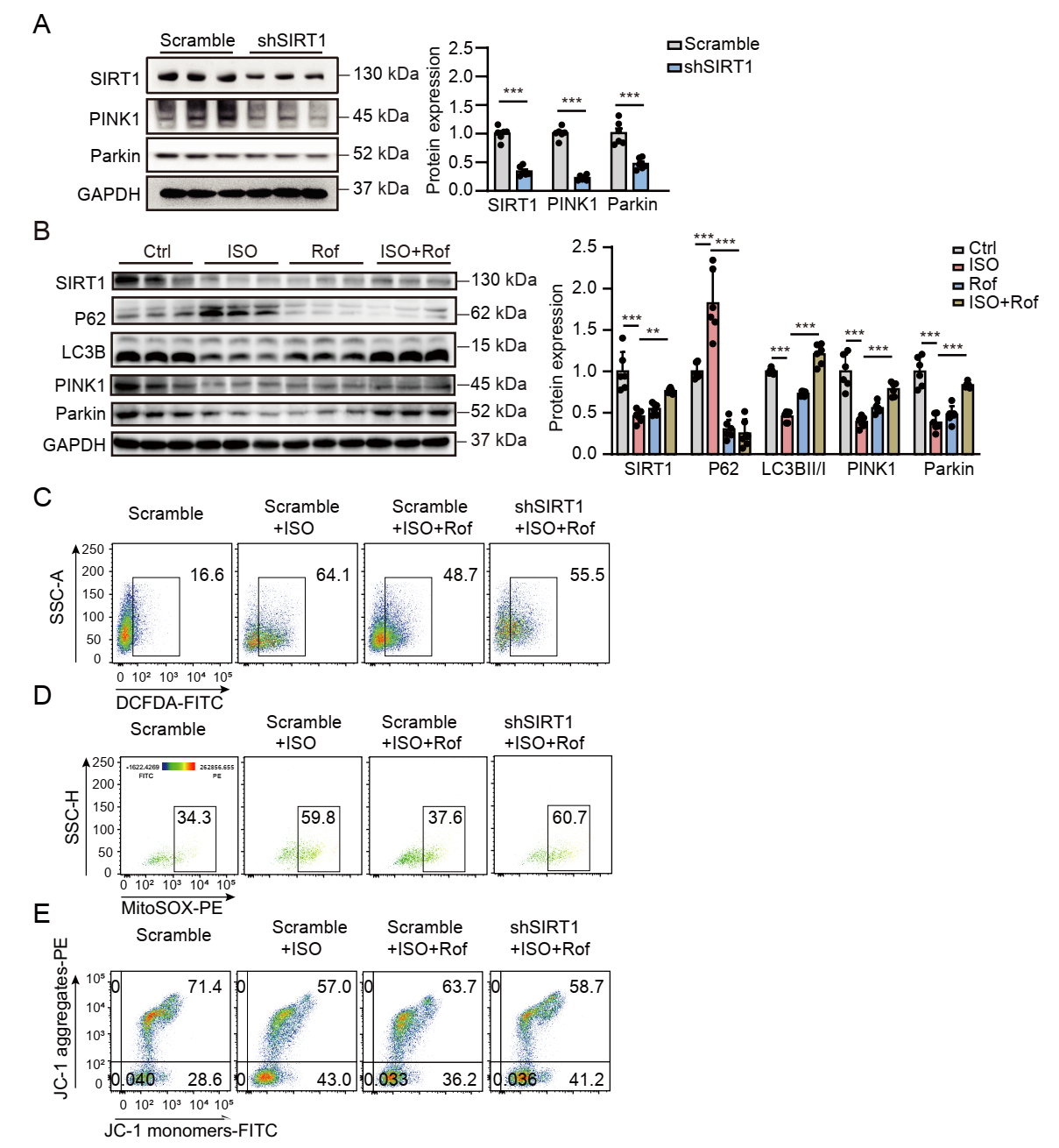
**Supplemental Figure 10.** **SIRT1 knockdown abolishes the protection of PDE4 inhibitor on ISO-reduced mitophagy in cardiomyocytes.**

**A,** Representative immunoblotting images and quantification of SIRT1, PINK1, and Parkin, n=6 independent experiments. **B,** Representative immunoblots and quantification of SIRT1, P62, LC3BII/I, PINK1, and Parkin protein expressions in heart tissues from indicated groups in ISO injection experiment; n=6 mice per group. **C** through **E,** NRVMs were transfected with indicated shRNAs (scramble or shSIRT1) for 48 hours (**A**) and treated with ISO (10 μM) for another 24 hours in the presence or absence of roflumilast (1 μM). FACS analyses of cellular ROS levels with DCFH-DA staining (**C**), mitochondria ROS levels with MitoSOX staining (**D**), and the level of ΔΨm by the ratio of JC-1 aggregates to monomers (**E**), quantifications are shown in **Figure. 4K** through **4M**. All data are presented as mean±SEM. Unpaired 2-tailed Student t test was used for **A**; One-way ANOVA with Tukey's multiple comparison test was used for **B**.


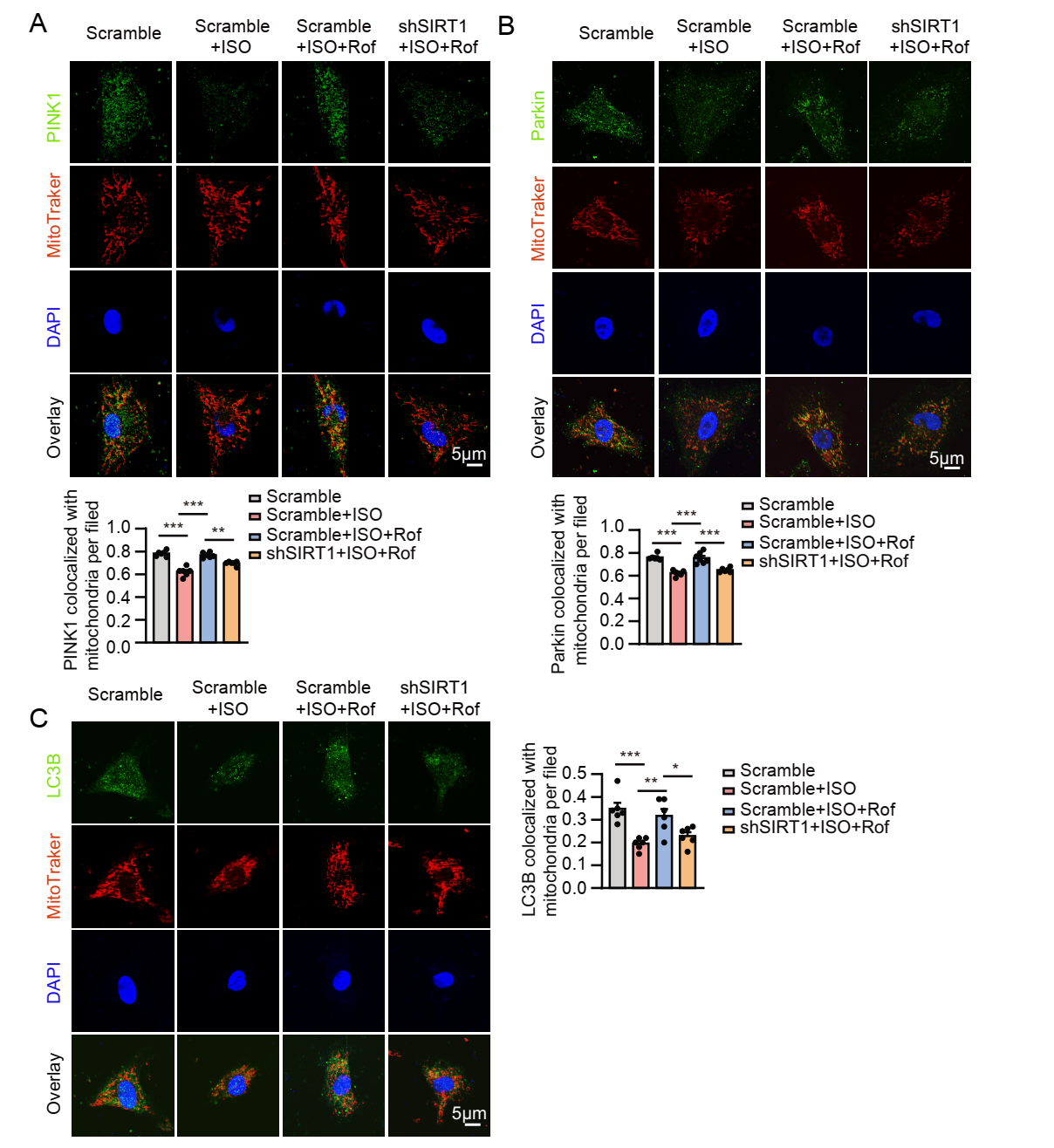
**Supplemental Figure 11. SIRT1 knockdown abolishes the restoration of PDE4 inhibitor on ISO-reduced colocalization of PINK1, Parkin and LC3B with mitochondria in cardiomyocytes.**

NRVMs were transfected with indicated shRNA for 48 hours and treated with ISO (10 μM) for another 24 hours in the presence or absence of roflumilast (1 μM). Representative immunofluorescence co-staining and Pearson's correlation coefficient for colocalization of MitoTracker (red) with PINK1 (green) (**A**), Parkin (green) (**B**) or LC3B (green) (**C**) in NRVMs; n=24 cells from 6 independent experiments. Scale bar, 5 µm. All data are presented as mean±SEM. Data were analyzed by one-way ANOVA with Tukey's multiple comparison test.


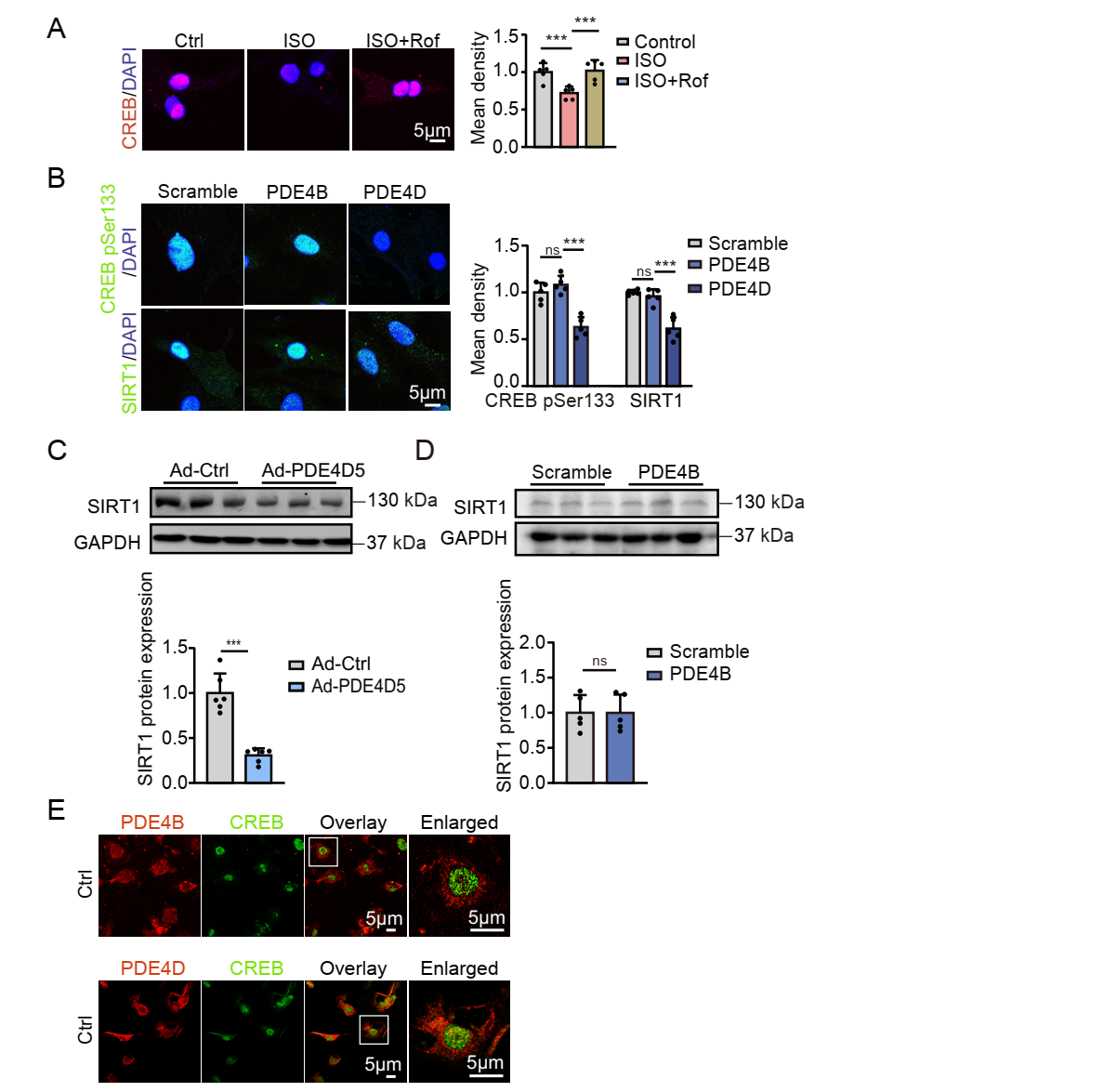


**Supplemental Figure 12. PDE4D but not PDE4B inhibits CREB-SIRT1 signaling.**

**A,** Representative immunofluorescence and quantification of CREB (red) in NRVMs; n=4 independent experiments. Scale bar, 5 µm. **B,** NRVMs were transfected with PDE4B, PDE4D plasmid or control plasmid (Scramble) for 72 hours. Representative immunofluorescence and quantification of CREB pSer133 (green) and SIRT1 (green) in NRVMs; n=5 independent experiments. Scale bar, 5 µm. **C,** PDE4D5 overexpression reduces SIRT1 protein expression in NRVMs; n=6 independent experiments. **D,** PDE4B overexpression has no effect on SIRT protein expression in NRVMs; n=5 independent experiments. **E,** Representative immunofluorescence co-staining of CREB (green) with PDE4B or PDE4D (red) in NRVMs. Scale bar, 5 µm. All data are presented as mean±SEM. One-way ANOVA with Tukey's multiple comparison test was used for **A** and **B**; Unpaired 2-tailed Student t test was used for **C** and **D**.


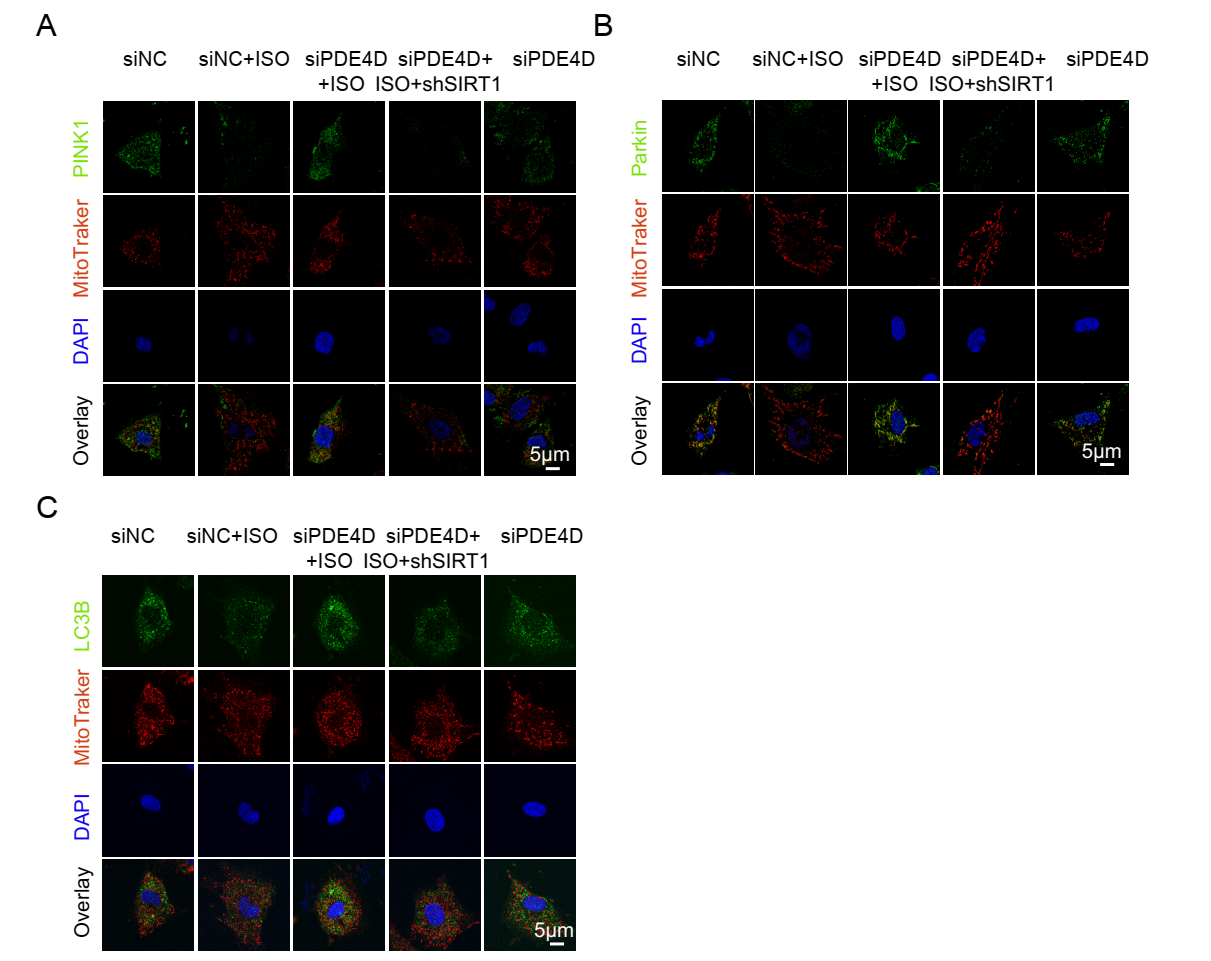


**Supplemental Figure 13. SIRT1 knockdown abolishes the restoration of PDE4D silencing on ISO-reduced colocalization of PINK1, Parkin and LC3B with mitochondria in cardiomyocytes.**

**A** through **C,** NRVMs were transfected with PDE4D siRNA (siPDE4D) and SIRT1 shRNA or negative control siRNA (siNC) for 48 hours and then treated with vehicle or ISO (10 µM) for another 24 hours. Images are single channels from **Figure. 5F** presented with merged color channels. Representative immunofluorescence co-staining of MitoTracker (red) with PINK1 (green) (**A**), Parkin (green) (**B**) or LC3B (green) (**C**) in NRVMs, respectively (the quantification are shown in **Figure 5F**); Scale bar, 5 µm.


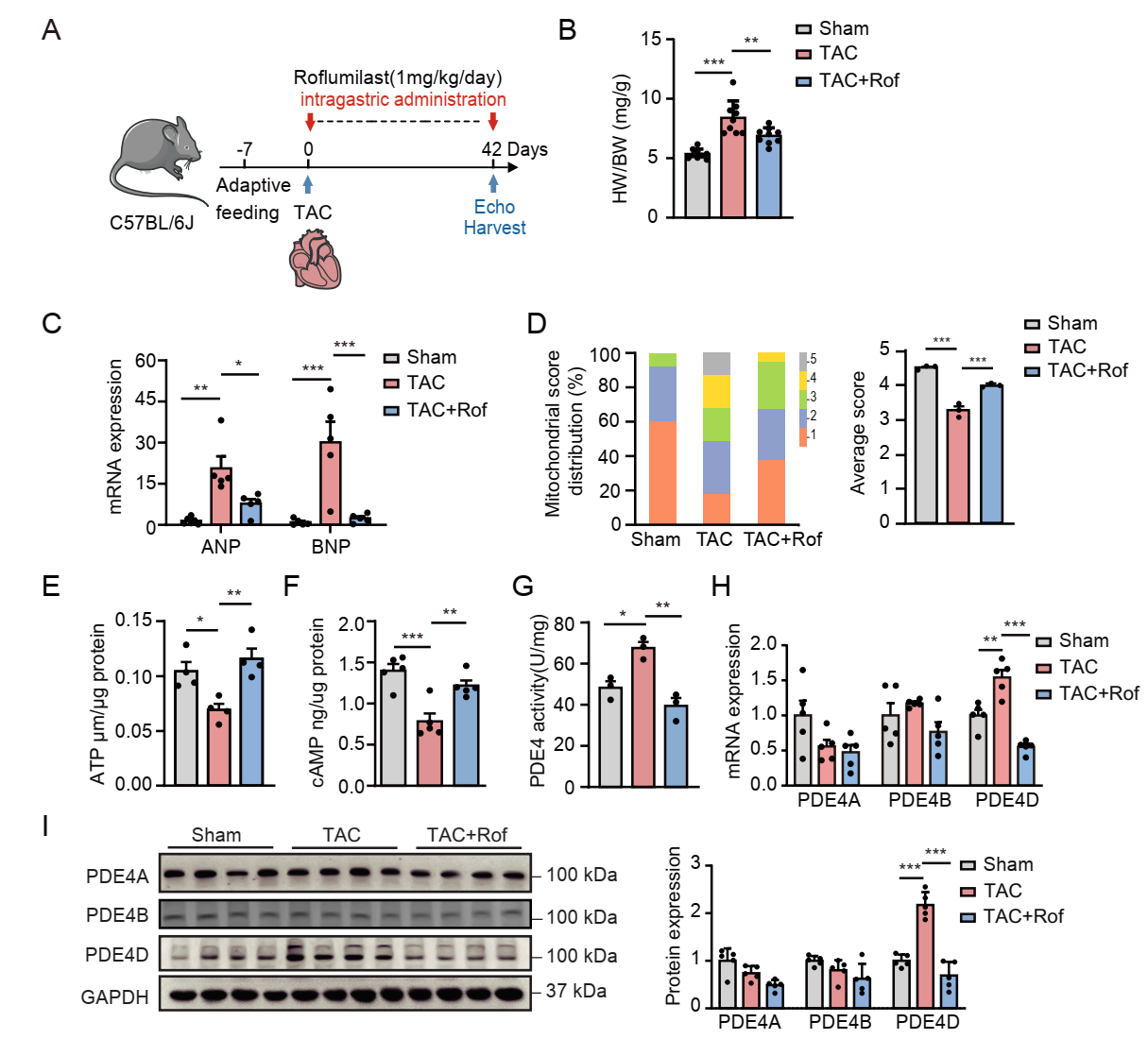


**Supplemental Figure 14. PDE4 inhibitor attenuates TAC-induced cardiac PDE4D expression and hypertrophy.**

**A,** Experimental scheme. 8-week-old male C57BL/6J mice were randomly assigned to 3 groups: Sham, TAC, and TAC+roflumilast. Each group was administered an equal volume of vehicle or roflumilast (1 mg/kg) by daily oral gavage for 6 weeks after the TAC or sham operation. **B,** Ratio of heart weight (HW) to body weight (BW); n=9 mice per group. **C,** Transcriptional expression of ANP and BNP in heart tissues; n=5 mice per group. **D.** Relative distribution and average score of mitochondrial cristae score determined by transmission electron microscopy, n=3 mice per group. **E,** Myocardial ATP levels in heart tissues; n=4 mice per group. **F,** cAMP levels in heart tissue; n=5 mice per group. **G,** PDE4 activity in the heart tissues; n=3 mice per group. **H,** Transcriptional expression of PDE4 isoforms (PDE4A, PDE4B and PDE4D); n=5 mice per group. **I,** Representative immunoblots and quantification of PDE4 isoforms (PDE4A, PDE4B and PDE4D) expression; n=5 mice per group. All data are presented as mean±SEM. Data were analyzed by one-way ANOVA with Tukey's multiple comparison test.


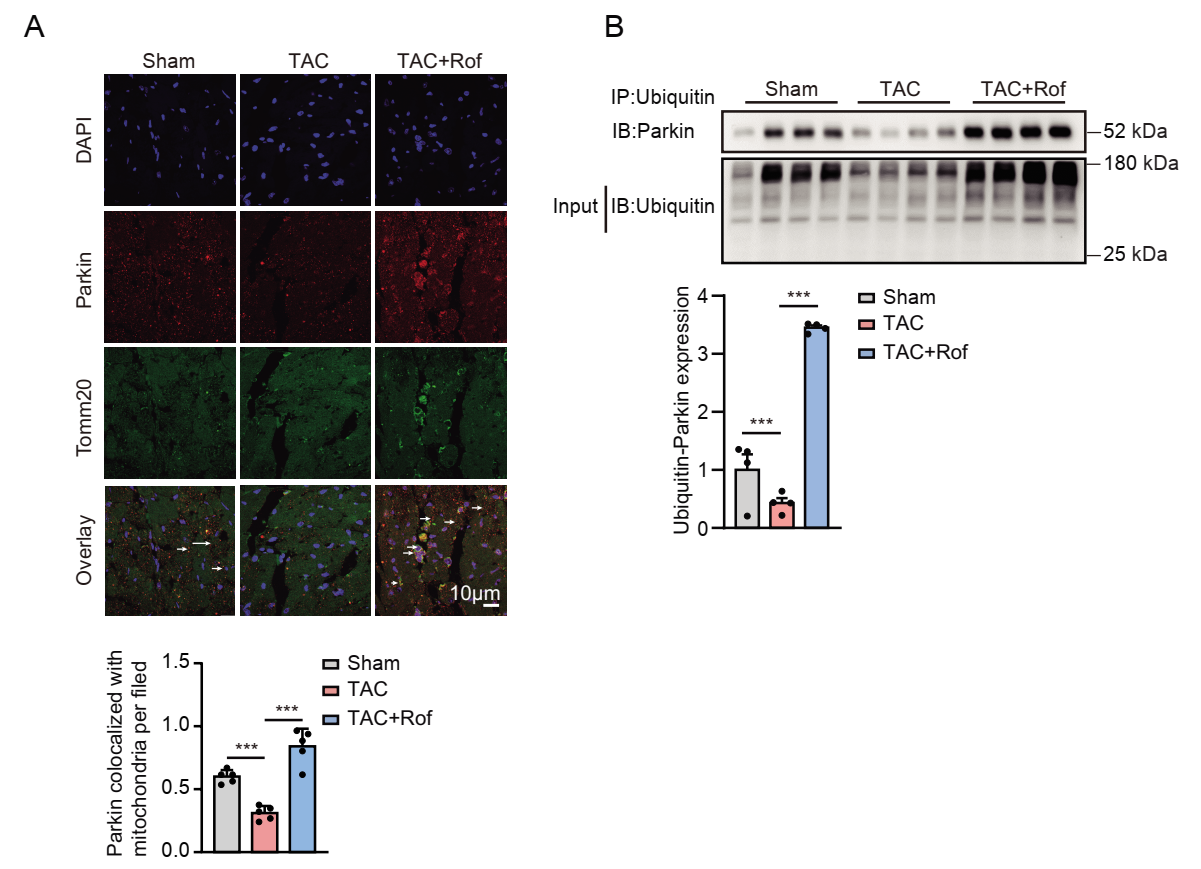
**Supplemental Figure 15. PDE4 inhibitor promotes Parkin recruitment to mitochondria in TAC mouse hearts.**

**A,** Representative immunofluorescence co-staining and Pearson's correlation coefficient for colocalization of Parkin (red) and Tomm20 (green) in cardiac sections; n=5 mice per group. Scale bar, 10 μm. **B,** Heart tissue lysate was subjected to immunoprecipitation using an anti-ubiquitin antibody and then immunoblotted with anti-Parkin antibodies. n=4 mice per group. All data are presented as mean±SEM. Data were analyzed by one-way ANOVA with Tukey's multiple comparison test.


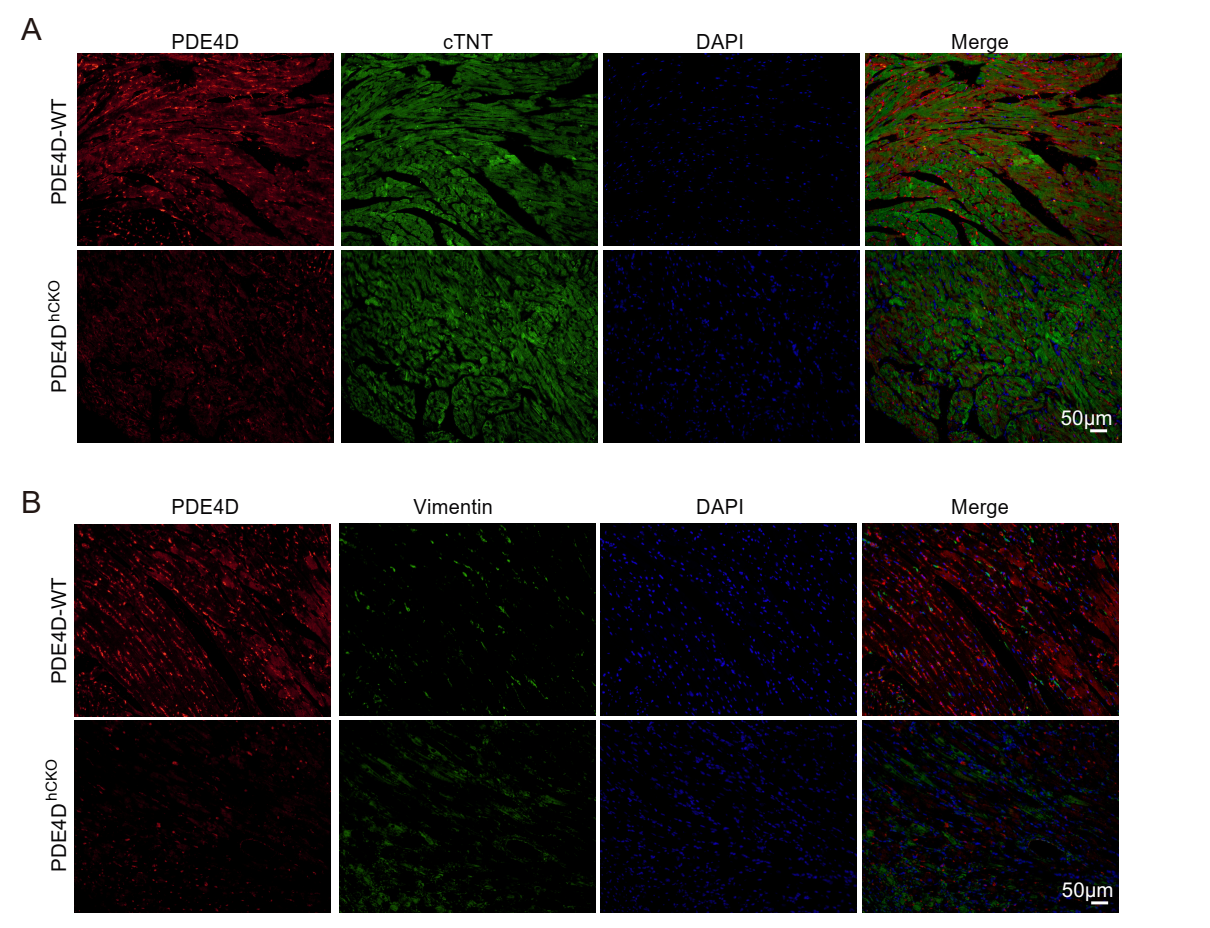


**Supplemental Figure 16. PDE4D expression is decreased in cardiomyocyte in PDE4D^hCKO^ mice.**

**A,** Immunofluorescence staining of PDE4D (red) and cTNT (green) in cardiac sections of PDE4D-WT and PDE4D^hCKO^ mice, Scale bar, 50 μm. **B,** Immunofluorescence staining of PDE4D (red) and Vimentin (green) in cardiac sections of PDE4D-WT and PDE4D^hCKO^ mice, Scale bar, 50 μm.


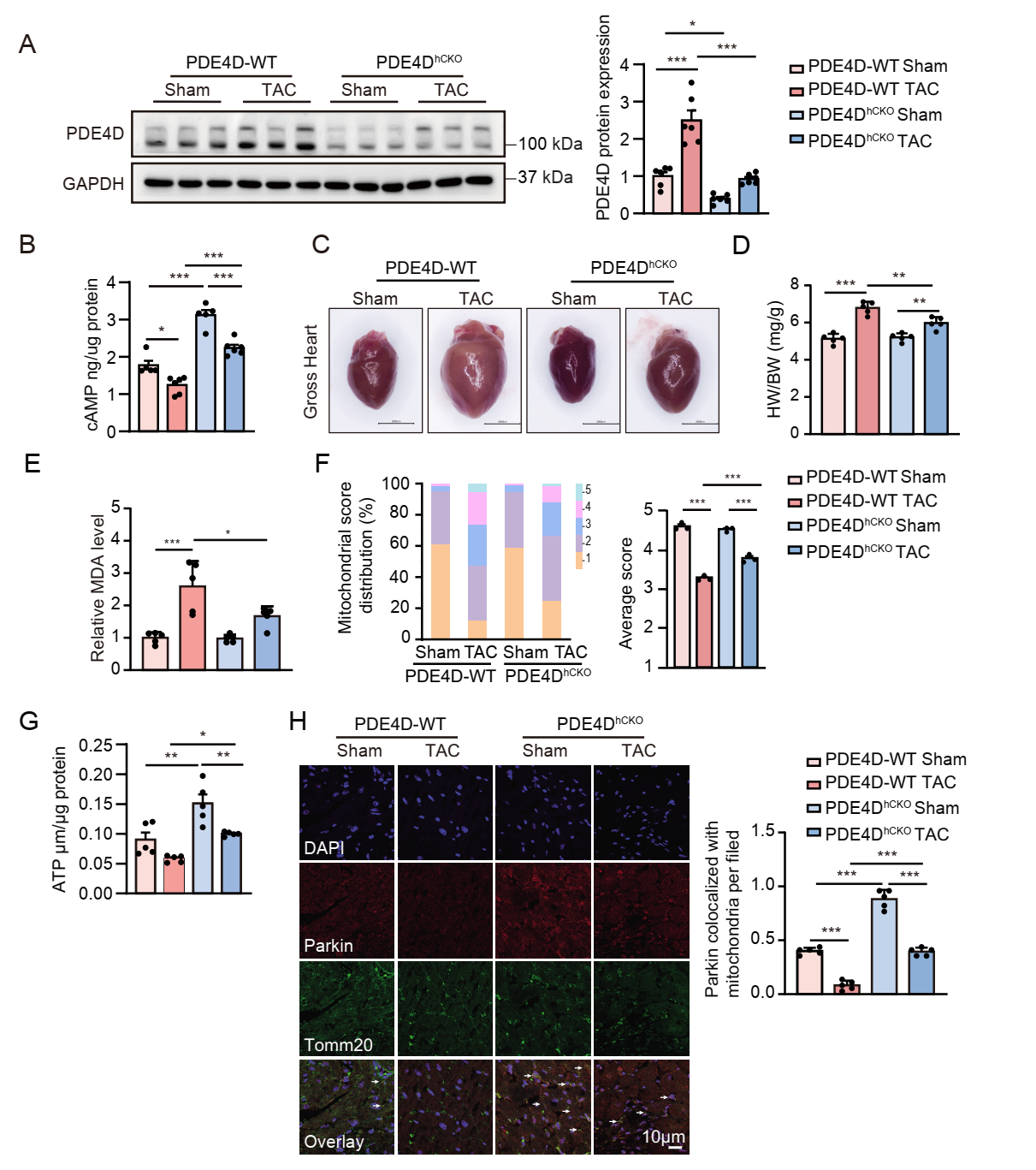


**Supplemental Figure 17. Cardiac-specific PDE4D-knockdown attenuates TAC-induced cardiac PDE4D expression and hypertrophy.**

αMHC-Cre+/PDE4Dflox/+ mice were subjected to 5 consecutive days of a single intraperitoneal injection of tamoxifen (100 mg/kg/day) at 30 days after TAC to induce PDE4D ablation in the heart (PDE4D^hCKO^), control littermate αMHC-Cre-/PDE4Dflox/+ mice with the same dose of tamoxifen as controls (PDE4D-WT). The mice were sacrificed 12 days after tamoxifen injection. **A,** Representative immunoblots and quantification of PDE4D expression in heart tissues; n=6 mice per group. **B,** cAMP levels in heart tissues; n=5-6 mice per group. **C.** Representative images of gross cardiac morphology. Scale bar, 2000 µm. **D.** Ratio of heart weight to bodyweight (HW/BW); n=5 mice per group. **E**, Quantitative analysis of relative MDA levels, n=5 mice per group. **F**, Relative distribution and average score of mitochondrial cristae score determined by transmission electron microscopy, n=3 mice per group. **G.** Myocardial ATP levels from the indicated groups; n=5 mice per group. **H,** Representative immunofluorescence co-staining and Pearson's correlation coefficient for colocalization of Parkin (red) and Tomm20 (green) in cardiac sections; n=5 mice per group. Scale bar, 10 μm. All data are presented as mean±SEM. Data were analyzed by two-way ANOVA with Tukey’s multiple comparison test.

**Supplemental Figure 18. PDE4D expression in cardiomyocyte in AAV9-PDE4D5 mouse and PDE4D**^+/-^ **mouse.**

**A,** Immunofluorescence staining of PDE4D (red) and cTNT (green) in cardiac sections of AAV9-NC and AAV9-PDE4D5 mice, Scale bar, 50 μm. **B,** Immunofluorescence staining of PDE4D (red) and Vimentin (green) in cardiac sections of AAV9-NC and AAV9-PDE4D5 mice, Scale bar, 50 μm. **C**. Immunofluorescence staining of PDE4D (red) and cTNT (green) in cardiac sections of WT-TAC and PDE4D^+/-^-TAC mice, Scale bar, 50 μm.


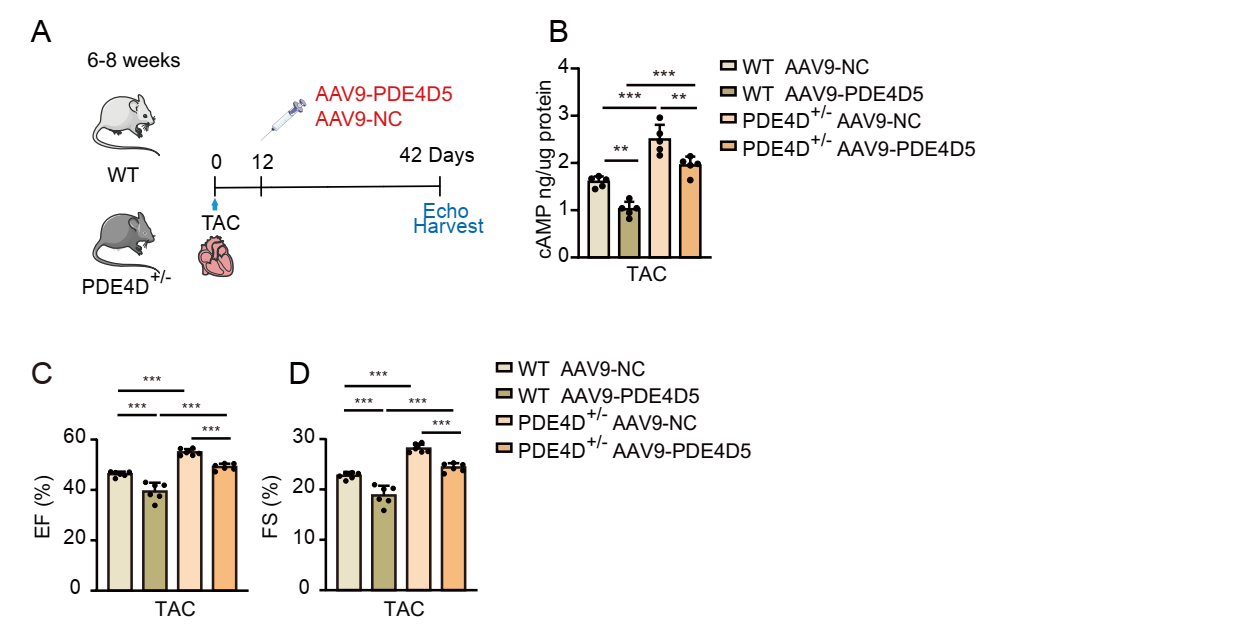


**Supplemental Figure 19. Cardiac overexpression of PDE4D5 counteracts the cardiac protective effects on heart contractile function exerted by PDE4D knockout in TAC mice.**

**A,** WT and PDE4D^+/-^ mice were subjected to TAC surgery and treated with adeno-associated virus serotype 9 (AAV9)-PDE4D5 or AAV9-NC 12 days after TAC. 6 weeks after surgery, echocardiography was performed and the hearts were harvested. **B,** cAMP levels in heart tissues; n=5 mice per group. **C** and **D,** Ejection fraction (**C**) and fractional shortening (**D**) measured by echocardiography 6 weeks after TAC; n=6 mice per group. All data are presented as mean±SEM. Two-way ANOVA with Tukey’s multiple comparison test was used for **B** through **D**.


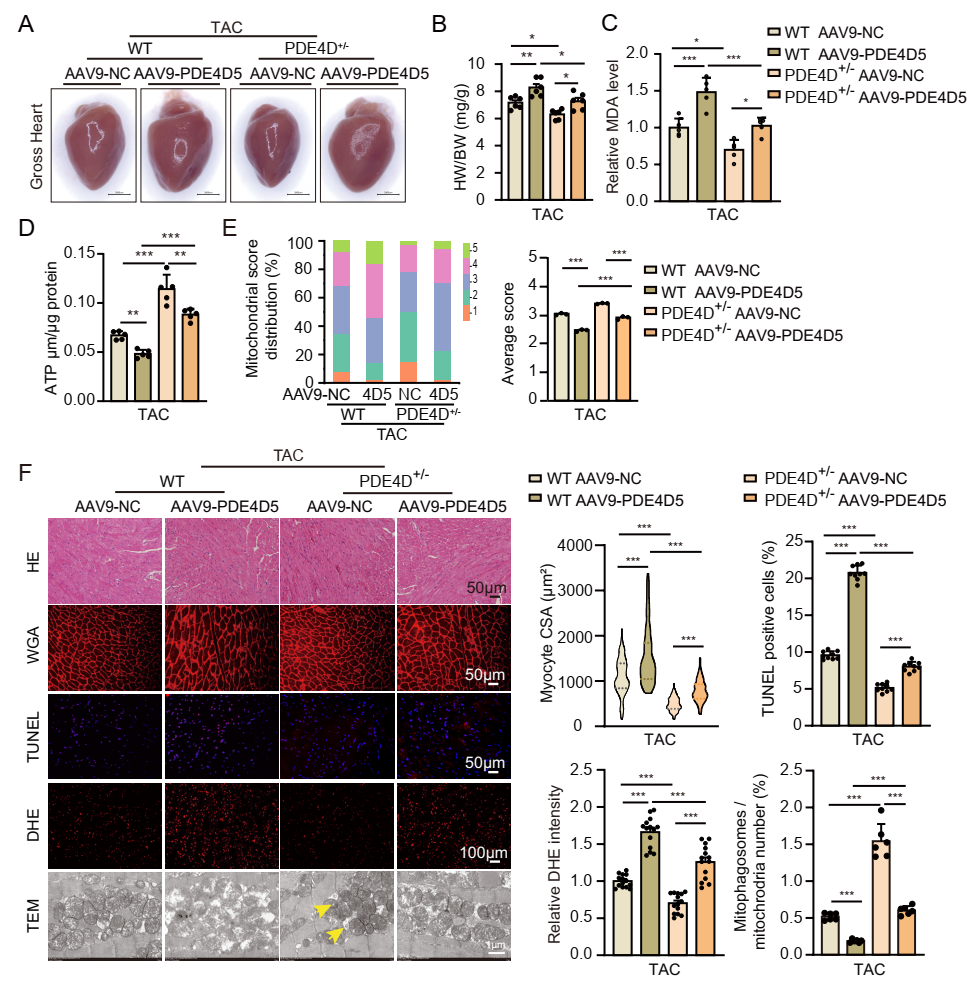


**Supplemental Figure 20. Cardiac overexpression of PDE4D5 counteracts the cardiac protective effects exerted by PDE4D knockout in TAC mice.**

WT and PDE4D^+/-^ mice were subjected to TAC surgery and treated with adeno-associated virus serotype 9 (AAV9)-PDE4D5 or AAV9-NC 12 days after TAC. 6 weeks after surgery, echocardiography was performed and the hearts were harvested. **A** through **D,** Gross morphology of the heart (**A**), the ratio of heart weight to body weight (HW/BW) (**B**), relative MDA levels(**C**), and myocardial ATP levels (**D**); n=5-6 mice per group. Scale bar, 2000 µm. **E**, Relative distribution and average score of mitochondrial cristae score determined by transmission electron microscopy, n=3 mice per group. **F,** Cardiac cross-sections were stained with hematoxylin-eosin or WGA to examine heart morphology and measure myocyte CSA; n=100 cells per heart and n=3 mice per group. Scale bar, 50 μm. The myocardial cell apoptosis rate was detected by TUNEL staining, 3 random fields per heart; n=3 mice per group. Scale bar, 50 µm. ROS levels were determined by measuring DHE fluorescence intensity in 5 random fields per heart; n=3 mice per group. Scale bar, 100 µm. Mitochondrial morphology was determined using TEM, and the number of mitophagosomes was quantitated. The arrows indicate mitophagosomes; 2 random fields per heart, n=3 mice per group. Scale bar, 1 μm. All data are presented as mean±SEM. Two-way ANOVA with Tukey’s multiple comparison test was used for **B** through **E**; nested ANOVA analyses were done for **F**.


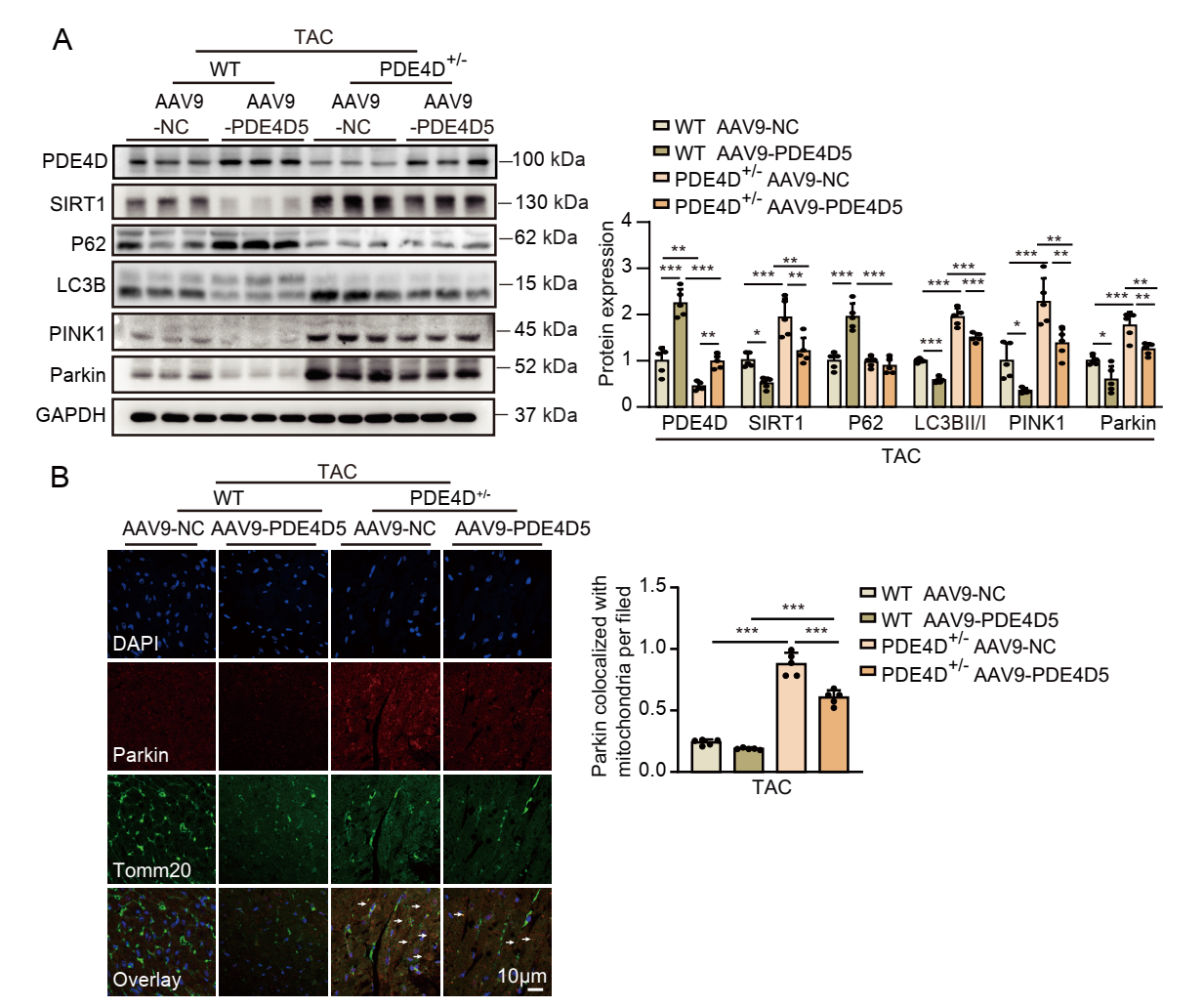


**Supplemental Figure 21. Cardiac overexpression of PDE4D5 inhibits PDE4D knockout-induced mitophagy in TAC mice.**

WT and PDE4D^+/-^ mice were subjected to TAC surgery and treated with adeno-associated virus serotype 9 (AAV9)-PDE4D5 or AAV9-NC at 12 days after TAC. 6 weeks after surgery, echocardiography was performed and the hearts were harvested. **A,** Representative immunoblots showing PDE4D, SIRT1, P62, LC3B, PINK1 and Parkin expression; n=5 mice per group. **B,** Representative immunofluorescence co-staining and Pearson's correlation coefficient for colocalization of Parkin (red) and Tomm20 (green) in cardiac sections; n=5 mice per group. Scale bar, 10 μm. All data are presented as mean±SEM. Data were analyzed by two-way ANOVA with Tukey’s multiple comparison test.

**SUPPLEMENTAL TABLES**

**Supplemental Table 1. Clinical characteristics of donors and heart failure patients.**

According to the regulations on organ donation in China, the information of donors is

confidential.

| **Patient** | **Diagnosis** | **Histopathological** | **Age** | **Gender** | **EF** |
| --- | --- | --- | --- | --- | --- |
|  |  | **examination** |  |  |  |
| Donor 1 | N/A | N/A | N/A | N/A | N/A |
| Donor 2 | N/A | N/A | N/A | N/A | N/A |
| Donor 3 | N/A | N/A | N/A | N/A | N/A |
| HF 1 | DCM, MTVI,  HF (IV) | hypertrophy of myocardial fibers with large nuclei | 32 | Male | 24% |
| HF 2 | DCM, MTVI, PAH, HF (IV) | hypertrophy of myocardial fibers with large nuclei | 70 | Male | 15% |
| HF 3 | DCM, PAH, HF (IV) | hypertrophy of myocardial fibers with large nuclei | 38 | Male | 27% |
| HF 4 | DCM, MTVI,  HF (IV) | N/A | 57 | Male | 38% |
| HF 5 | DCM, MTVI,  HF (IV) | hypertrophy of myocardial fibers with large nuclei | 32 | Male | 17% |
| HF 6 | DCM, MTVI, PAH, HF (IV) | hypertrophy of myocardial fibers with large nuclei | 47 | Male | 29% |
| HF7 | DCM, MTVI,  HF (IV) | N/A | 48 | Male | 30% |

DCM, dilated cardiomyopathy; EF, ejection fraction; HF (IV), NYHA class IV heart failure; MTVI, mitral and tricuspid valve insufficiency; N/A, not available; PAH, pulmonary arterial hypertension;

**Supplemental Table 2. Echocardiographic parameters of mice treated with isoproterenol injection and administered roflumilast.**

|  | Control | ISO | Rof | ISO+ Rof |
| --- | --- | --- | --- | --- |
| EF % | 58.87±2.327 | 42.71±1.76^***^ | 55.97±3.11 | 54.91±2.00^&&^ |
| FS % | 30.80±1.65 | 20.70±1.00^***^ | 28.96±2.10 | 28.04±1.33^&&^ |
| LVID; d(mm) | 3.74±0.05 | 3.86±0.07 | 3.73±0.06 | 3.73±0.08 |
| LVID; s(mm) | 2.64±0.08 | 3.06±0.06 | 2.70±0.09 | 2.71±0.09 |
| LVPW; d(mm) | 0.70±0.02 | 0.73±0.04 | 0.80±0.04 | 0.74±0.03 |
| LVPW; s(mm) | 1.02±0.05 | 0.88±0.04 | 1.06±0.06 | 1.00±0.03 |
| LV Mass (mg) | 98.94±2.62 | 101.6±4.17 | 102.4±5.75 | 99.86±7.30 |
| LVMass (corrected) | 79.15±2.097 | 81.26±3.337 | 81.88±4.60 | 79.89±5.84 |
| LV Vol; d(μL) | 60.13±2.14 | 64.80±2.79 | 59.81±2.33 | 59.93±3.09 |
| LV Vol; s(μL) | 26.00±1.92 | 37.08±1.92^**^ | 27.86±2.44 | 27.90±2.31^&^ |
| LVAW; d(mm) | 0.82±0.03 | 0.77±0.04 | 0.76±0.02 | 0.78±0.04 |
| LVAW; s(mm) | 1.25±0.03 | 1.08±0.03 | 1.15±0.05 | 1.18±0.04 |

***P*<0.01, ****P*<0.001, ISO vs Control group; ^&^*P*<0.05, ^&&^*P*<0.01, ISO+Rof vs ISO group. All values are expressed as mean±SEM. n=9 mice per group. ISO, isoproterenol; ROF, roflumilast.

EF, ejection fraction; FS, fractional shortening; LVID; d, Left ventricular internal diameter end diastole and end-diastole; LVID; s, Left ventricular internal diameter end diastole and end systole; LVPW; d, Left ventricular posterior wall end diastole; LVPW; s, Left ventricular posterior wall end systole; LV Mass, left ventricular mass; LV Mass (corrected), left ventricular corrected mass; LV Vol; d, left ventricular volume end diastole; LV Vol; s, left ventricular volume end systole; LVAW; d, left ventricular anterior end diastole; LVAW; s, left ventricular anterior end systole. Data were analyzed by one-way ANOVA with Tukey's multiple comparison test.

**Supplemental Table 3. Echocardiographic parameters of mice treated with TAC surgery and administered roflumilast.**

|  | Sham | TAC | TAC+Rof |
| --- | --- | --- | --- |
| EF % | 52.96±2.00 | 39.90±1.87^**^ | 53.09±3.14^&&^ |
| FS % | 27.13±1.29 | 19.37±1.05^**^ | 27.04±1.94^&&^ |
| LVID; d(mm) | 4.38±0.04 | 4.39±0.08 | 4.20±0.16 |
| LVID; s(mm) | 3.34±0.07 | 3.55±0.08 | 3.17±0.21 |
| LVPW; d(mm) | 0.79±0.02 | 0.99±0.04^*^ | 1.11±0.08 |
| LVPW; s(mm) | 1.02±0.03 | 1.30±0.06^**^ | 1.38±0.06 |
| LV Mass (mg) | 108.20±4.86 | 142.3±9.46^***^ | 120.80±7.19 |
| LVMass (corrected) | 86.56±3.89 | 111.10±7.49^*^ | 96.60±5.75 |
| LV Vol; d(μL) | 65.90±2.82 | 66.91±4.30 | 67.26±3.55 |
| LV Vol; s(μL) | 31.00±2.25 | 29.98±3.24 | 29.53±3.24 |
| LVAW; d(mm) | 0.67±0.03 | 1.03±0.05^***^ | 0.98±0.07 |
| LVAW; s(mm) | 1.08±0.04 | 1.41±0.04^***^ | 1.45±0.07 |

**P*<0.05, ***P*<0.01, ****P*<0.001 TAC vs Sham group; ^&&^*P*<0.01 TAC+Rof vs TAC group. All values are expressed as mean±SEM. Data were analyzed by one-way ANOVA with Tukey's multiple comparison test. n=9 mice per group.

**Supplemental Table 4. Echocardiographic parameters of PDE4D-WT and PDE4D^hCKO^ mice subjected to TAC surgery.**

|  | PDE4D-WT Sham | PDE4D-WT TAC | PDE4D^hCKO^Sham | PDE4D^hCKO^ TAC |
| --- | --- | --- | --- | --- |
| EF % | 66.21±2.32 | 44.60±1.97^***^ | 64.88±2.17 | 65.39±4.46^&&&^ |
| FS % | 36.05±1.83 | 21.85±1.10^***^ | 34.90±1.60 | 35.90±3.45^&&&^ |
| LVID; d(mm) | 3.67±0.06 | 4.01±0.13 | 3.54±0.10 | 3.43±0.07^&&&^ |
| LVID; s(mm) | 2.35±0.09 | 3.14±0.13^***^ | 2.28±0.12 | 2.20±0.14^&&&^ |
| LVPW; d(mm) | 0.84±0.04 | 0.81±0.05 | 0.89±0.05 | 0.82±0.07 |
| LVPW; s(mm) | 57.36±2.16 | 71.51±5.36 | 53.54±3.21 | 48.78±2.56 |
| LV Mass (mg) | 108.70±7.10 | 117.70±12.41 | 111.60±8.14 | 87.58±8.23 |
| LVMass (corrected) | 86.98±5.68 | 94.15±9.92 | 89.29±6.51 | 70.06±6.58 |
| LV Vol; d(μL) | 57.36±2.16 | 71.51±5.36^*^ | 53.54±3.21 | 48.78±2.56^&&&^ |
| LV Vol; s(μL) | 19.66±1.88 | 40.12±4.09^***^ | 18.96±1.81 | 17.26±2.54^&&&^ |
| LVAW; d(mm) | 0.81±0.04 | 0.74±0.03 | 0.85±0.04 | 0.70±0.03 |
| LVAW; s(mm) | 1.31±0.06 | 1.04±0.05 | 1.33±0.07 | 1.19±0.13 |

**P*<0.05, ****P*<0.001 PDE4D-WT TAC vs PDE4D-WT Sham group; ^&&&^*P*<0.001 PDE4D^hCKO^ TAC vs PDE4D-WT TAC group. All values are expressed as mean±SEM. Data were analyzed by one-way ANOVA with two-way ANOVA with Tukey’s multiple comparison test. n=8 mice per group.

**Supplemental Table 5. Echocardiographic parameters of WT and PDE4D^+/-^ mice subjected to TAC surgery and treated with AAV9-PDE4D5 or AAV9-NC.**

|  | WT  AAV9-NC | WT  AAV9-PDE4D5 | PDE4D^+/-^AAV9-NC | PDE4D^+/-^AAV9-PDE4D5 |
| --- | --- | --- | --- | --- |
| EF % | 46.29±0.41 | 39.38±1.42^***^ | 54.95±0.53^###^ | 49.15±0.53^&&& $$$^ |
| FS % | 22.72±0.26 | 18.82±0.78^***^ | 28.12±0.35^###^ | 24.36±0.34^&&& $$$^ |
| LVID; d(mm) | 3.90±0.07 | 3.92±0.04 | 4.00±0.10 | 3.79±0.10 |
| LVID; s(mm) | 3.02±0.05 | 3.19±0.04 | 2.88±0.08 | 2.86±0.07^&&^ |
| LVPW; d(mm) | 0.64±0.03 | 0.73±0.03 | 0.64±0.03 | 0.75±0.04 |
| LVPW; s(mm) | 0.78±0.04 | 0.91±0.04 | 0.95±0.04 | 0.91±0.04 |
| LVMass (mg) | 100.8±4.79 | 105.0±4.81 | 107.4±7.39 | 121.4±8.14 |
| LVMass (corrected) | 80.61±3.83 | 84.01±3.85 | 85.96±5.91 | 97.11±6.62 |
| LV Vol; d(μL) | 66.14±2.78 | 66.94±1.77 | 70.34±4.22 | 61.77±4.15 |
| LV Vol; s(μL) | 35.50±1.38 | 40.54±1.16 | 31.70±1.97 | 31.38±2.02^&&^ |
| LVAW; d(mm) | 0.83±0.06 | 0.77±0.07 | 0.85±0.06 | 0.99±0.08 |
| LVAW; s(mm) | 1.16±0.07 | 1.02±0.05 | 1.24±0.04 | 1.21±0.07 |

****P*<0.001 WT AAV9-PDE4D5 vs WT AAV9-NC group; ^$$$^*P*<0.001 PDE4D^+/-^AAV9-PDE4D5 vs PDE4D^+/-^AAV9-NC group; ^&&^*P*<0.01, ^&&&^*P*<0.001 PDE4D^+/-^AAV9-PDE4D5 vs WT AAV9-PDE4D5 group, ^###^*P*<0.001, PDE4D^+/-^AAV9-NC vs WT AAV9-NC group. All values are expressed as mean±SEM. Data were analyzed by one-way ANOVA with two-way ANOVA with Tukey’s multiple comparison test. n=6 mice per group.

**Supplemental Table 6. List of primers and siRNA target sequence.**

| **Gene** | **Species** | **Primer** | **Sequences (5'to3')** |
| --- | --- | --- | --- |
| PDE4A | Mouse | F | CTTCTGCGAGACCTGCTCCA |
|  |  | R | GAGTTCCCGGTTCAGCATCC |
| PDE4B | Mouse | F | AATGTGGCTGGGTACTCACA |
|  |  | R | AAGGTGTCAGATGAGATTTTAAACG |
| PDE4D | Mouse | F | ACCGCCAGTGGACGGACCGGA |
|  |  | R | CATGCCACGCTCCCGCTCTCGG |
| ANP | Mouse | F | TCGTCTTGGCCTTTTGGCT |
|  |  | R | TCCAGGTGGTCTAGCAGGTTCT |
| BNP | Mouse | F | CTCCTGAAGGTGCTGTCC |
|  |  | R | GCCATTTCCTCCGACTTT |
| 18s | Mouse | F | TTGACGGAAGGGCACCACCAG |
|  |  | R | GCACCACCACCCACGGAATCG |
| PDE4A | Rat | F | TTACTGTCACCTCGGGCCT |
|  |  | R | CTGCCTCCAAGCTGACACA |
| PDE4B | Rat | F | GTAATCCTCCAGCCTCGGTG |
|  |  | R | GACTGCAGACTAGACCTGGC |
| PDE4D | Rat | F | TACCTGTACTGCCGTGCCA |
|  |  | R | TGTCCACATCGAAACGTCTG |
| ANP | Rat | F | CAACACAGATCTGATGGATTTCA |
|  |  | R | CCTCATCTTCTACCGGCATC |
| 18s | Rat | F | TTGACGGAAGGGCACCACCAG |
|  |  | R | GCACCACCACCCACGGAATCG |
| siPINK1001 | Rat |  | GGAGAAGGCCAAACACCTT |
| siPINK1002 | Rat |  | GCCCAGATGTCGTCTCAAA |
| siPINK1003 | Rat |  | GCCTTGGGTTCAGCAAACA |
| siPDE4D | Rat |  | AGTTCGAACTAACCTTAGA |

**Supplemental Table 7. List of antibodies.**

| **Antibodies** | **Company** | **Catalog#** |
| --- | --- | --- |
| PDE4 | Abcam, Cambridge, UK | ab14628 |
| PDE4A | A gift from Marcoconti lab |  |
| PDE4B | Cloud clone, Wuhan, China | MAF642Ra24 |
| PDE4D3 | FabGennix, TX, USA | 431AP |
| PDE4D5 | FabGennix, TX, USA | 451AP |
| PDE4D9 | FabGennix, TX, USA | 491AP |
| PDE4D | Proteintech, Chicago, IL, USA | 12918-1-AP |
| PINK1 | Proteintech, Chicago, IL, USA | 23274-1-AP |
| Parkin | Proteintech, Chicago, IL, USA | 14060-1-AP |
| P62 | Bimake, Houston, TX, USA | A5180 |
| LC3B | Proteintech, Chicago, IL, USA | 18725-1-AP |
| PLB pSer16 | Millipore, Boston, USA | 07-052 |
| PLB | Millipore, Boston, USA | 05-205 |
| CREB pSer133 | Cell Signaling Technology, MA, USA | 9198S |
| CREB | Cell Signaling Technology, MA, USA | 9197S |
| CREB* | UpingBio, Hangzhou, China | YP-Ab-01142 |
| ubiquitin | Santa Cruz, CA, USA | sc-8017 |
| ANP | Santa Cruz, CA, USA | sc-515701 |
| Tomm20 | Santa Cruz, CA, USA | sc-17764 |
| VDAC1 | Bimake, Houston, TX, USA | A5224 |
| SIRT1 | Abclonal, Boston, MA, USA | A19667 |
| α-actinin | Sigma-Aldrich, MO, USA | A7811 |
| GAPDH | Proteintech, Chicago, IL, USA | 60004-1-Ig |
| Anti-Mouse IgG | Proteintech, Chicago, IL, USA | SA00001-1 |
| Anti-Rabbit IgG | Proteintech, Chicago, IL, USA | SA00001-2 |
| CoraLite488-conjugated Goat Anti-Mouse IgG | Proteintech, Chicago, IL, USA | SA00013-1 |
| CoraLite488-conjugated Goat Anti-Rabbit IgG | Proteintech, Chicago, IL, USA | SA00013-2 |
| CoraLite594-conjugated Goat Anti-Mouse IgG | Proteintech, Chicago, IL, USA | SA00013-3 |
| CoraLite594-conjugated Goat Anti-Rabbit IgG | Proteintech, Chicago, IL, USA | SA00013-4 |
| * CO-IF staining |  |  |

**Supplemental Table 8. List of Commercial Assays kit.**

| **Critical Assays kit** | **Company** | **Catalog#** |
| --- | --- | --- |
| cAMP-Glo™ Assay | Promega, WI, USA | V1502 |
| Lipofectamine™3000 Transfection Reagent | Invitrogen, CA, USA | L3000150 |
| Wheat Germ Agglutinin, Alexa Fluor™ 488 Conjugate | Invitrogen, CA, USA | W11261 |
| Wheat Germ Agglutinin, Alexa Fluor™ 594 Conjugate | Invitrogen, CA, USA | W11262 |
| MitoSOX™ Red mitochondrial superoxide indicator | Invitrogen, CA, USA | M36008 |
| One-Step Tunel Apoptosis Assay Kit | Beyotime, Nanjing, China | C1089 |
| Dihydroethidium | Beyotime, Nanjing, China | S0063 |
| ATP Assay Kit | Beyotime, Nanjing, China | S0026 |
| MDA Assay Kit | Beyotime, Nanjing, China | S0131 |
| Mitochondrial membrane potential assay kit with JC-1 | Beyotime, Nanjing, China | C2006 |
| ROS Assay Kit | Beyotime, Nanjing, China | S0033 |
| Mito-Tracker Red CMXRos | Beyotime, Nanjing, China | C1035 |
| Tissue Mitochondria Isolation Kit | Beyotime, Nanjing, China | C3606 |
| BCA Protein Assay Kit | Beyotime, Nanjing, China | P0012 |
| Mouse PDE4 Elisa Kit | Jianglai biotech, Shanghai, China |  |
