# Supplementary material for: PDE4D inhibition ameliorates cardiac hypertrophy and heart failure by activating mitophagy
Source: Redox Biol. 2025 Feb 22;81:103563. doi: 10.1016/j.redox.2025.103563 (PMC11909752; doi:10.1016/j.redox.2025.103563)

Full unedited blots for Figure 2B.

Red boxes indicates the lanes corresponding to those shown in the cropped images within the manuscript.

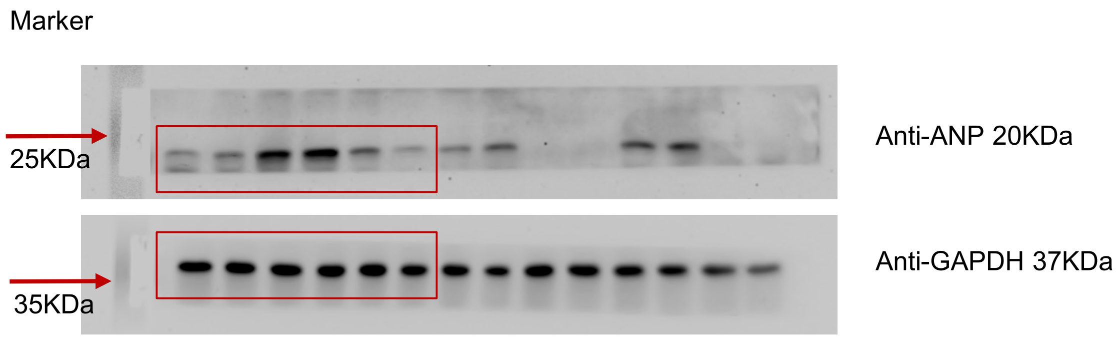

Full unedited blots for Figure 3A.

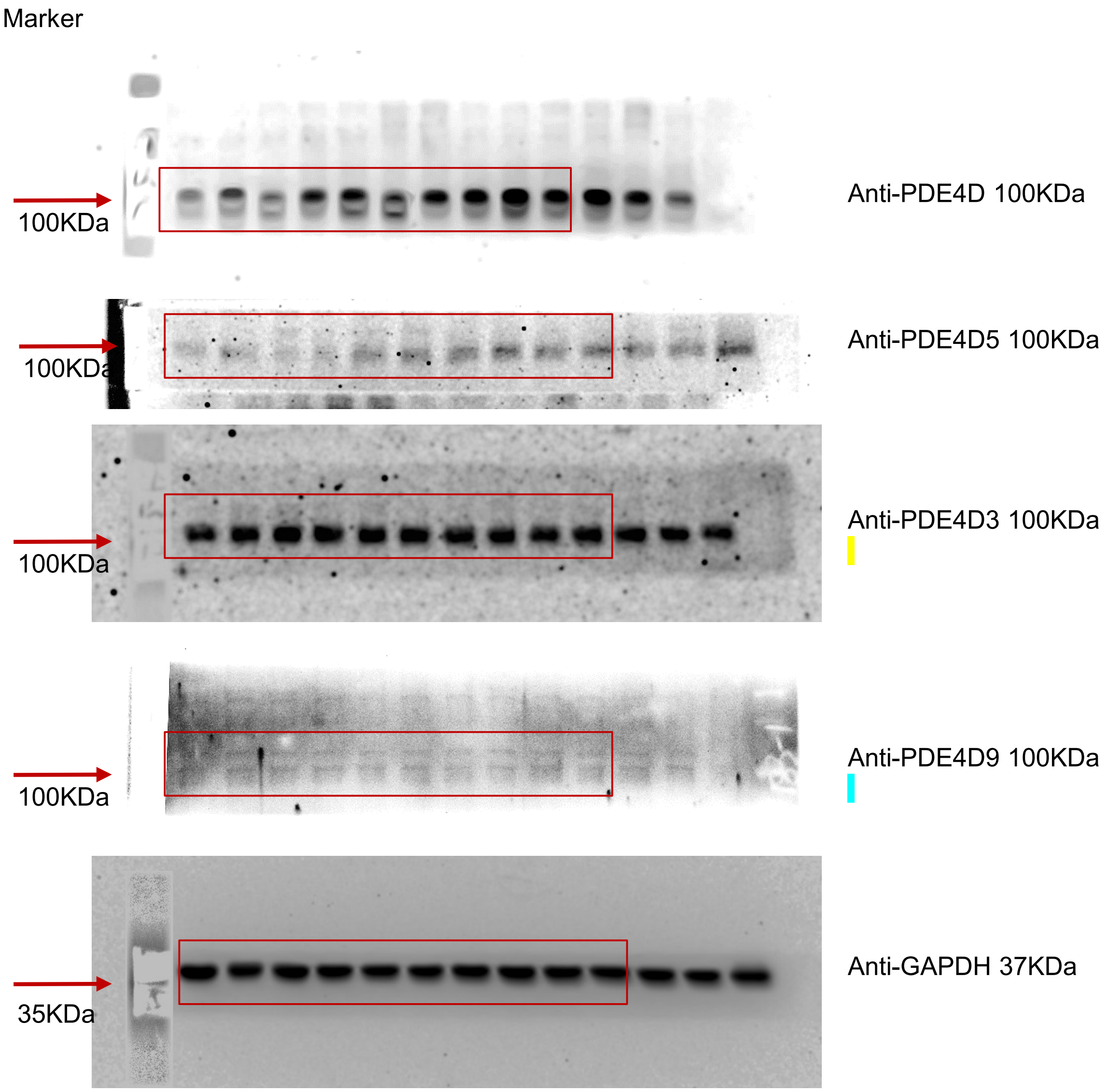

Full unedited blots for Figure 3B.

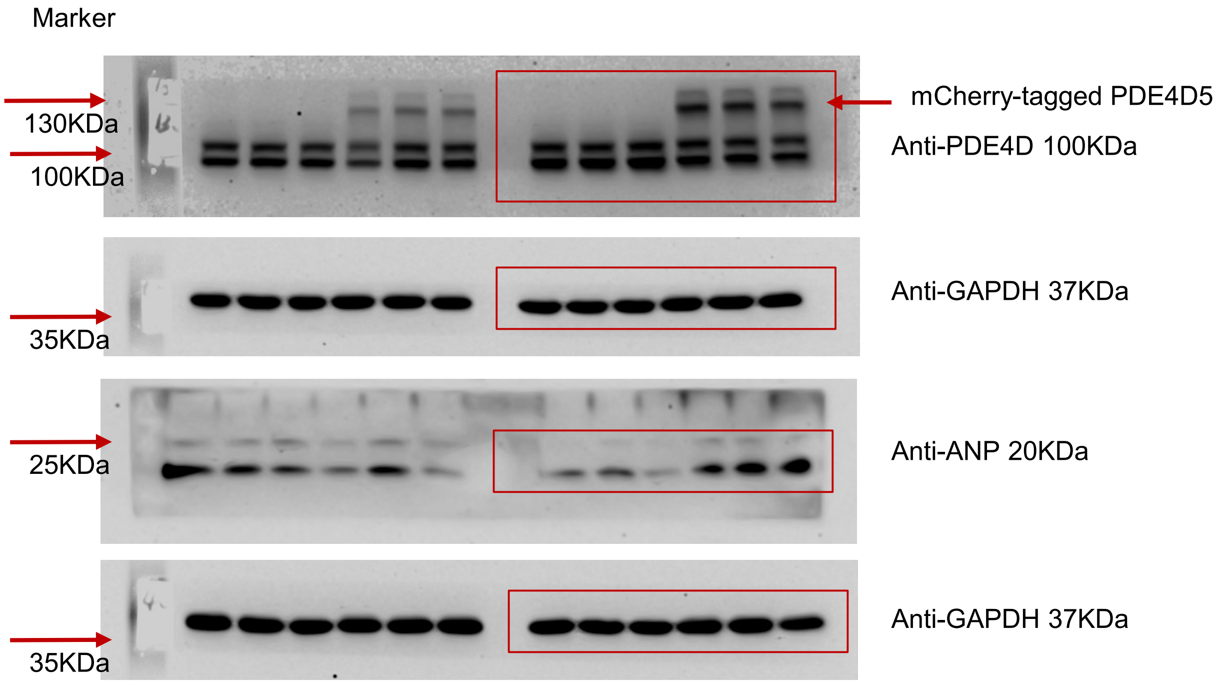

Full unedited blots for Figure 3H.

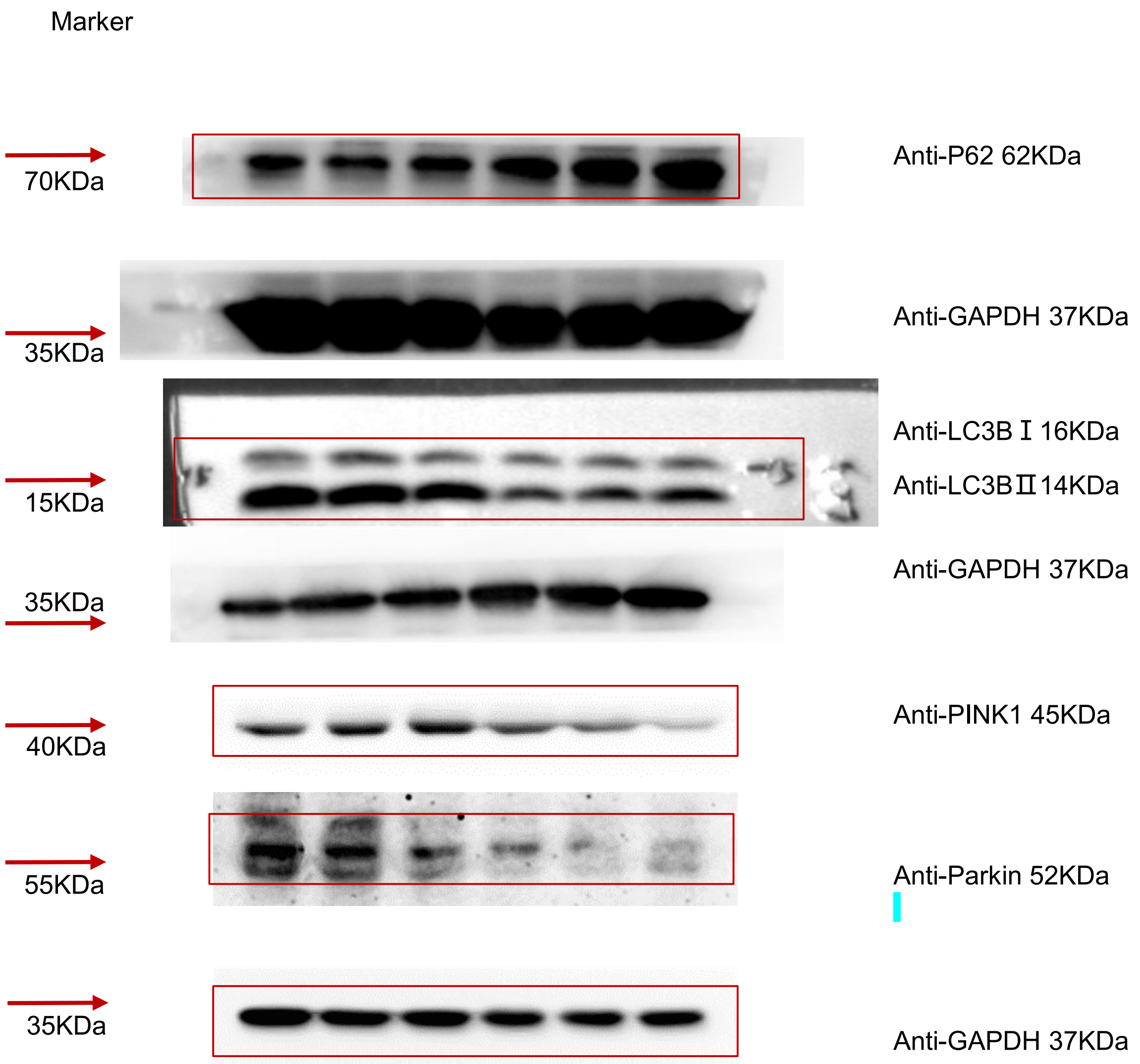

Full unedited blots for Figure 4A.

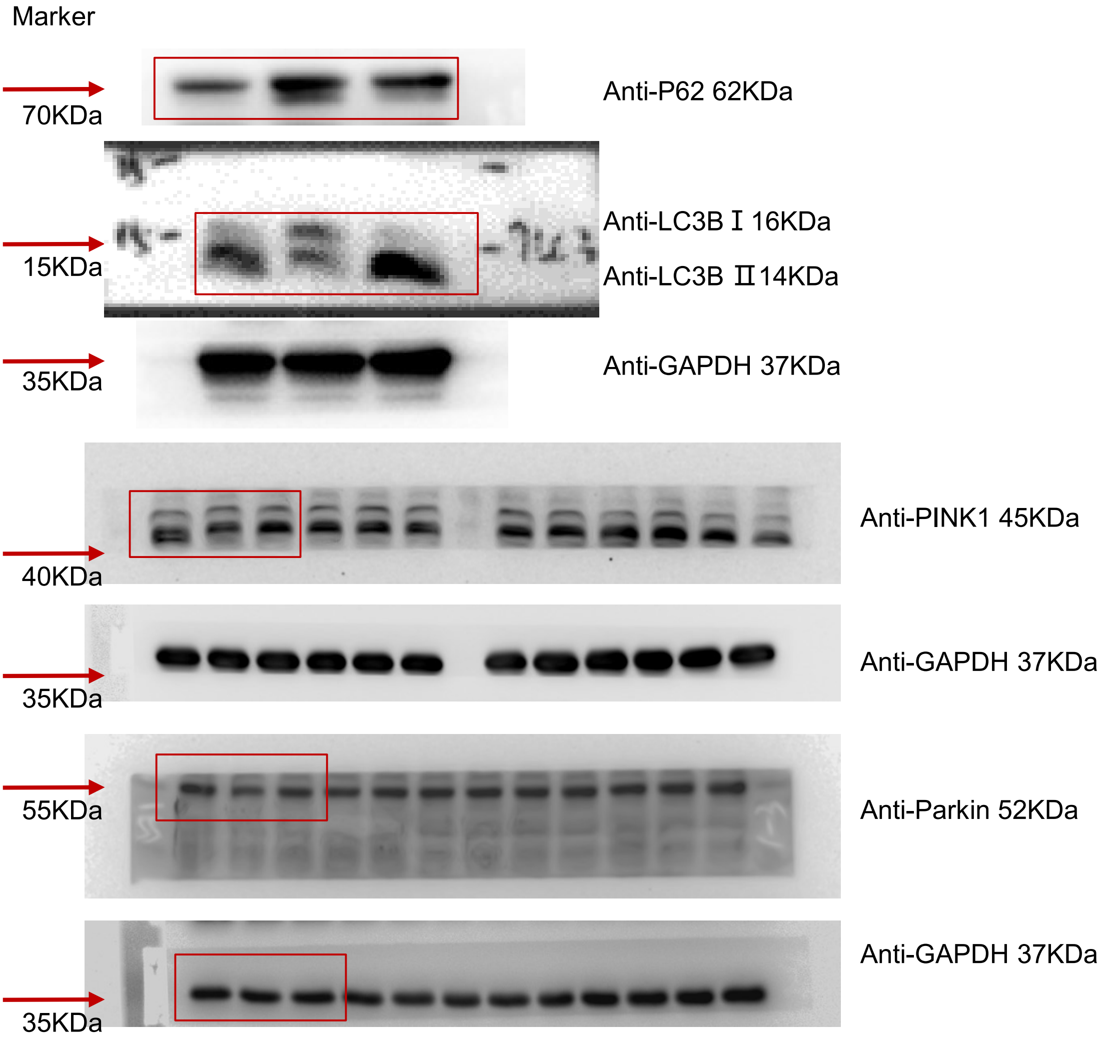

Full unedited blots for Figure 4D.

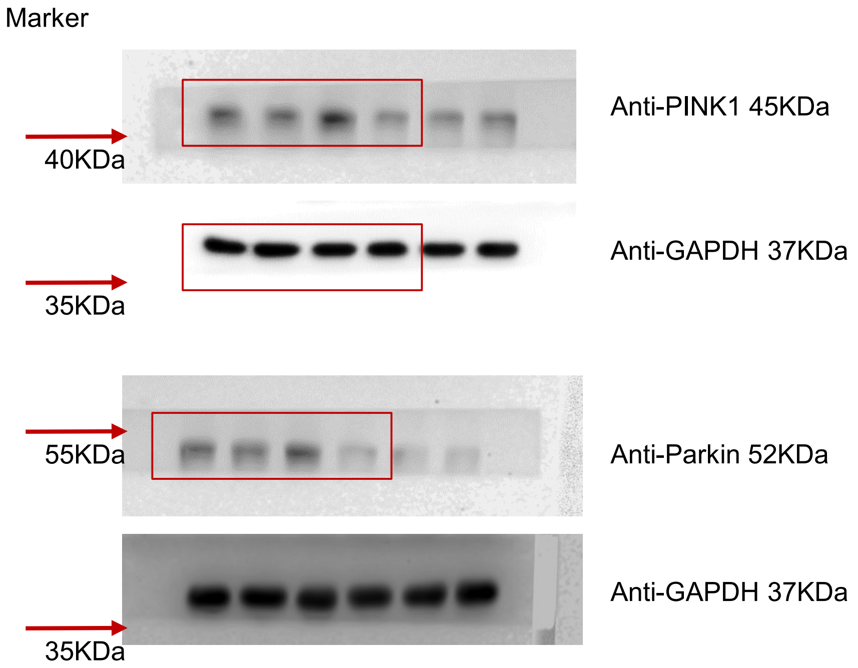

Full unedited blots for Figure 4I.

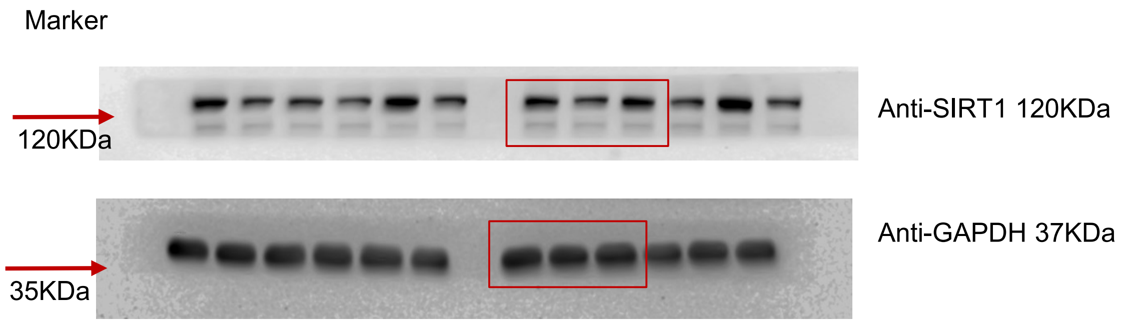

Full unedited blots for Figure 6K.

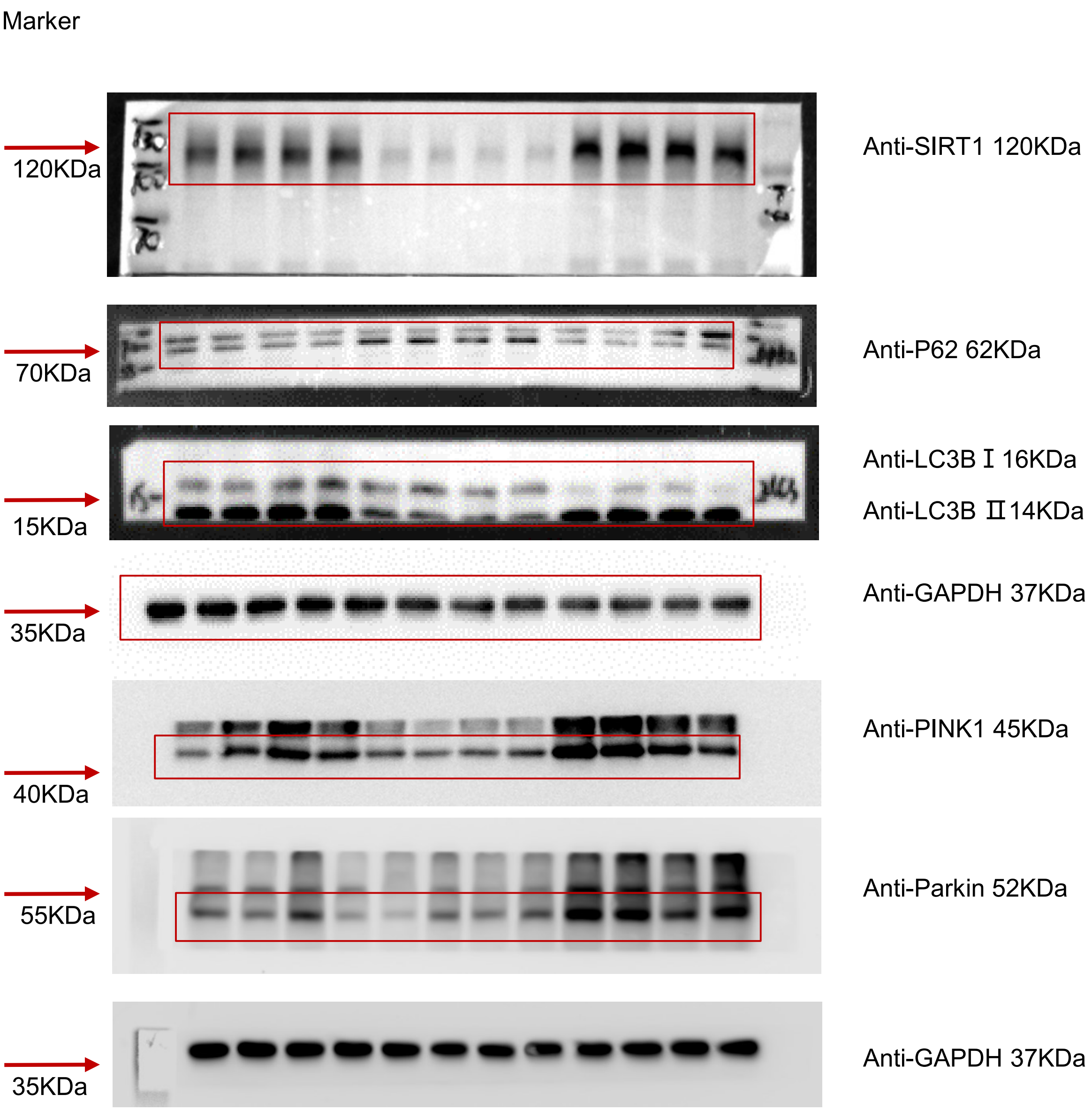

Full unedited blots for Figure 6L.

Marker

55KDa

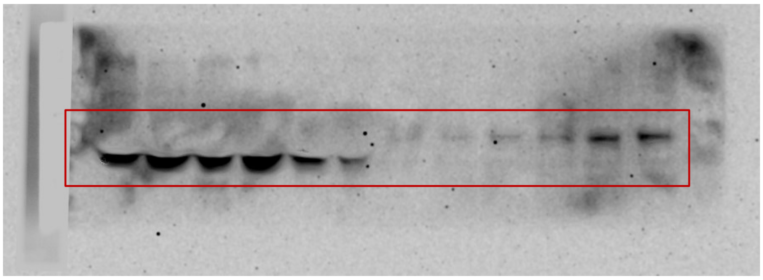

Anti-Parkin 52KDa

25KDa

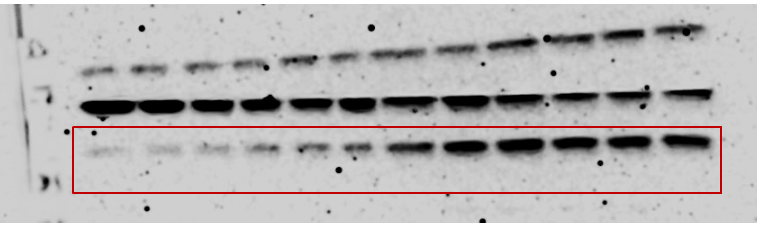

Anti-VDAC1 31KDa

35KDa

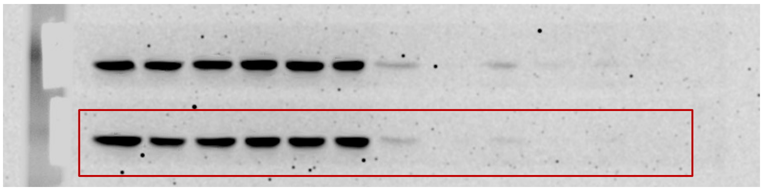

Anti-GAPDH 37KDa

Full unedited blots for Figure 7E.

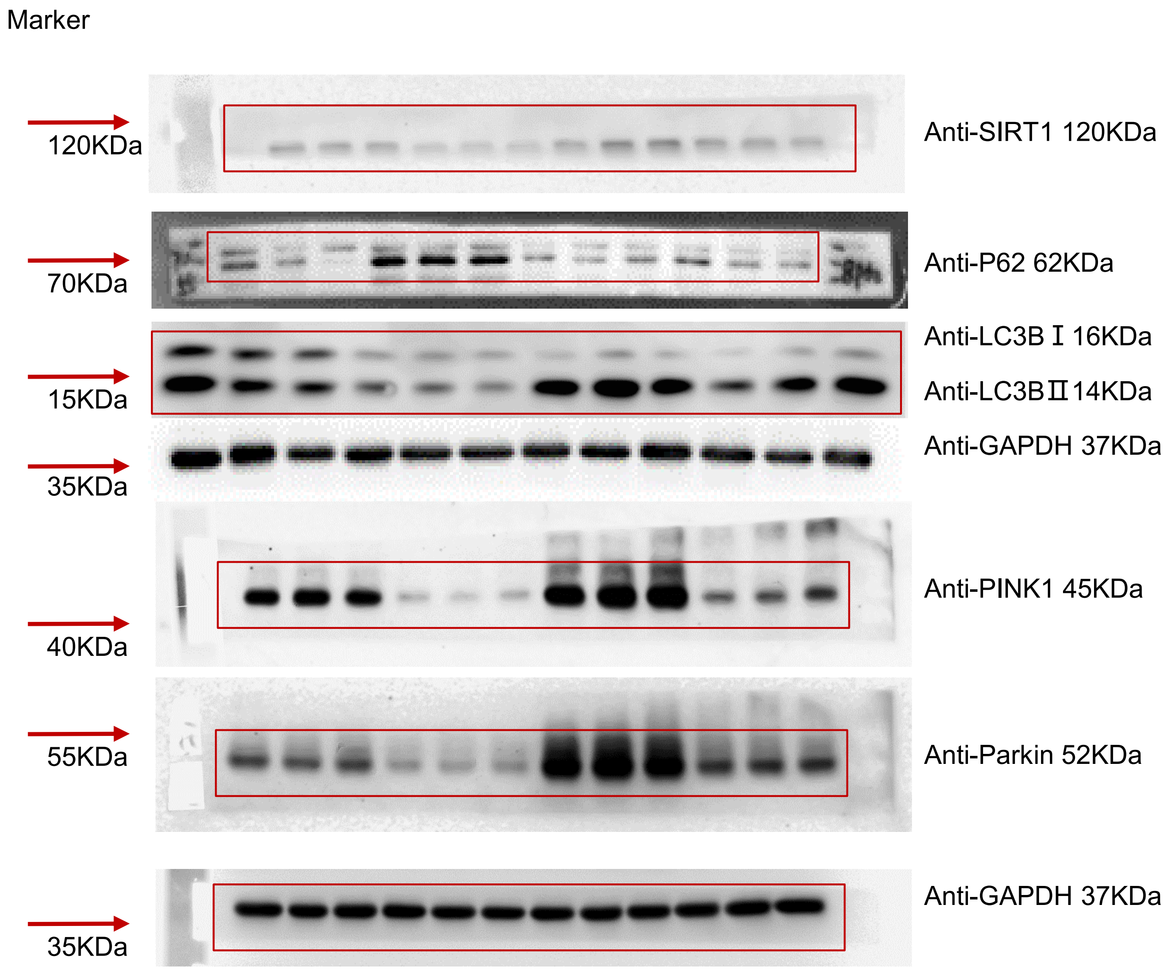

Full unedited blots for Supplymentary Figure 2B.

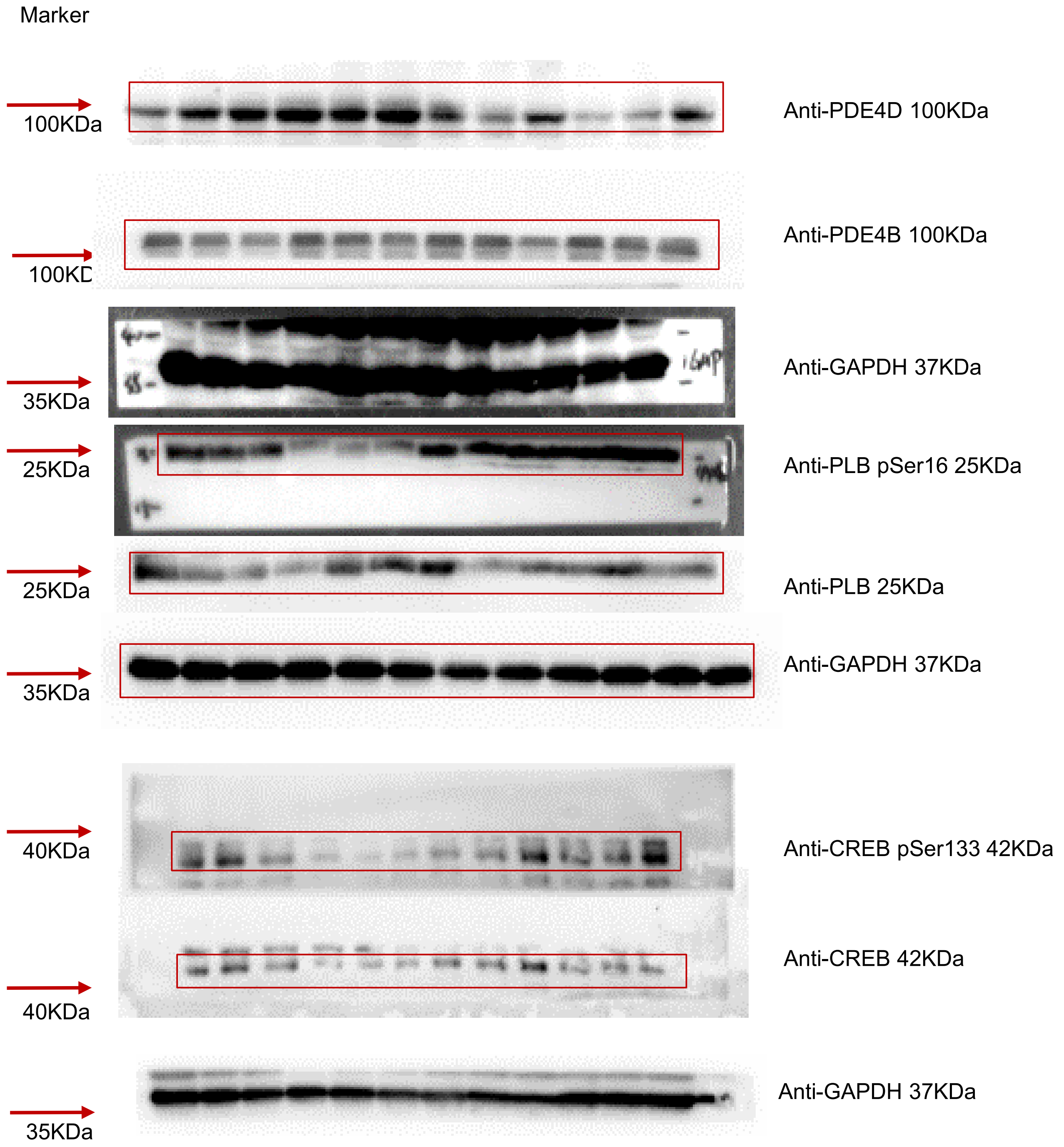

Full unedited blots for Figure 4A.

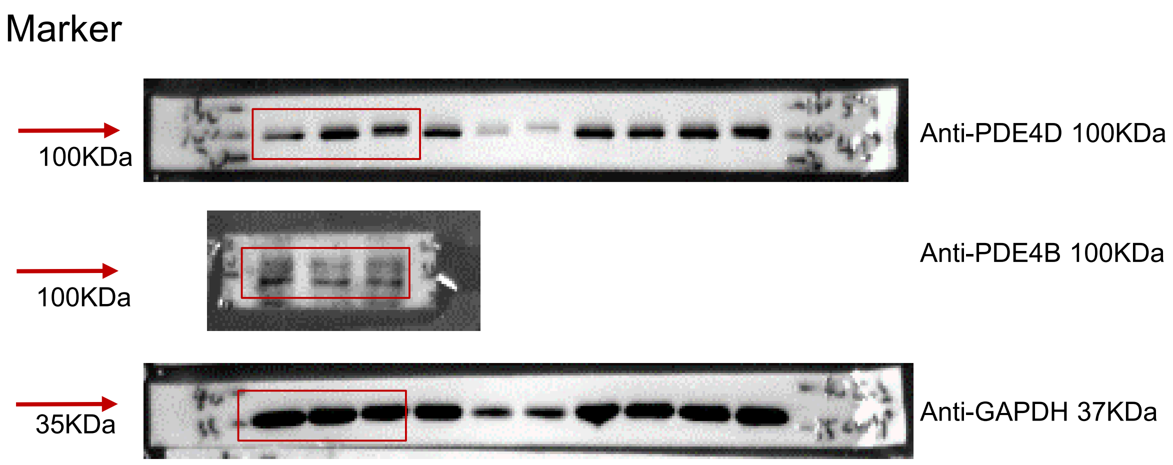

Full unedited blots for Supplymentary Figure 7A.

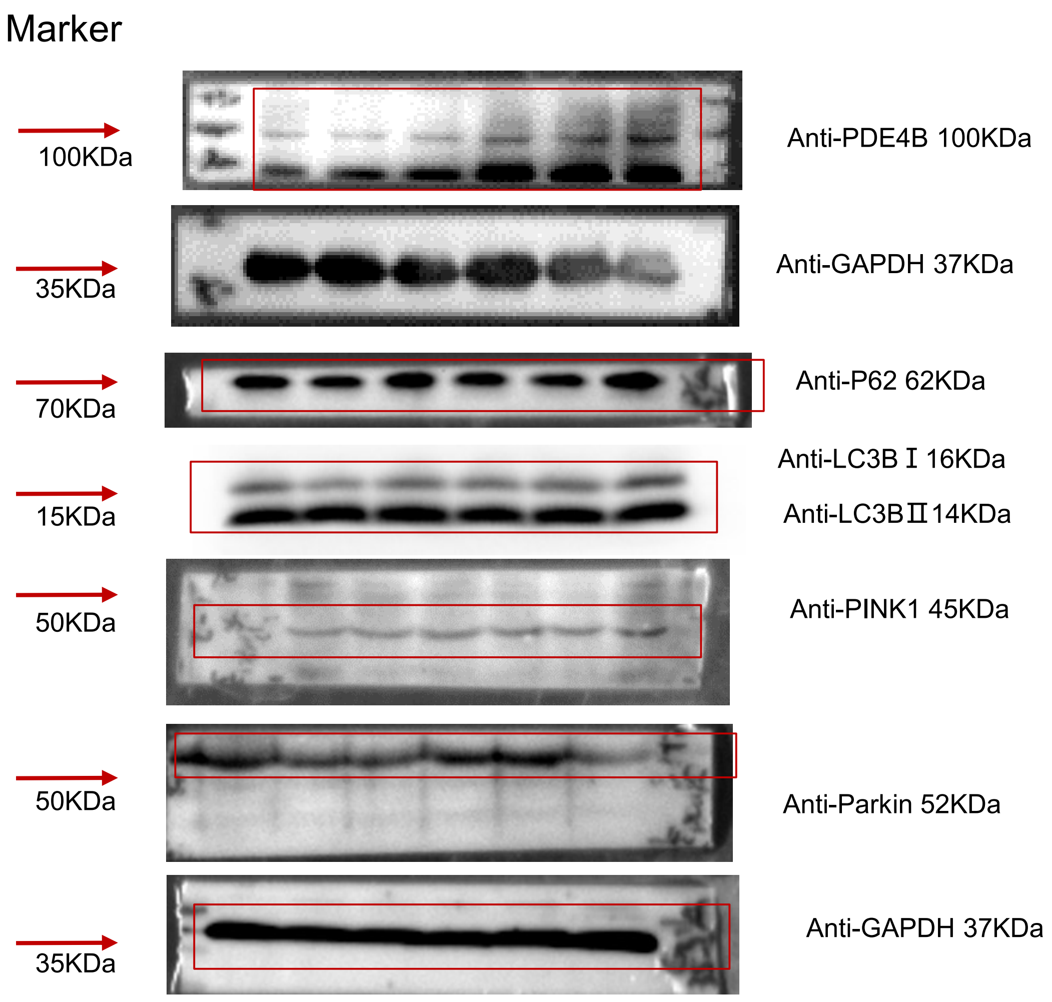

Full unedited blots for Supplymentary Figure 9A.

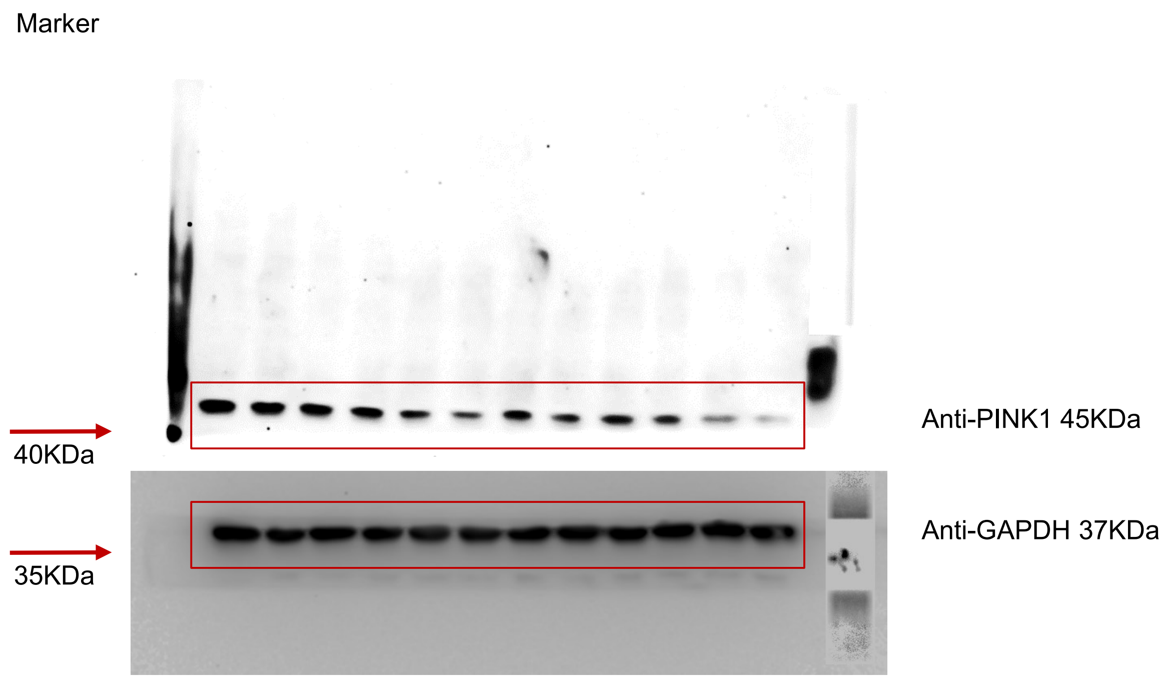

Full unedited blots for Supplymentary Figure 10A.

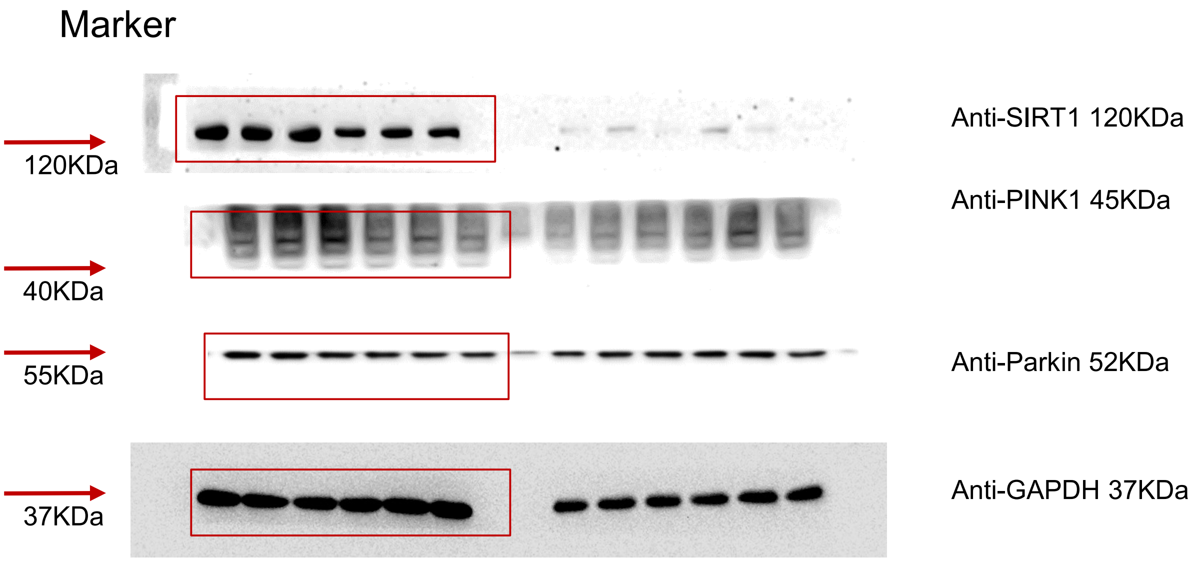

Full unedited blots for Supplymentary Figure 10B.

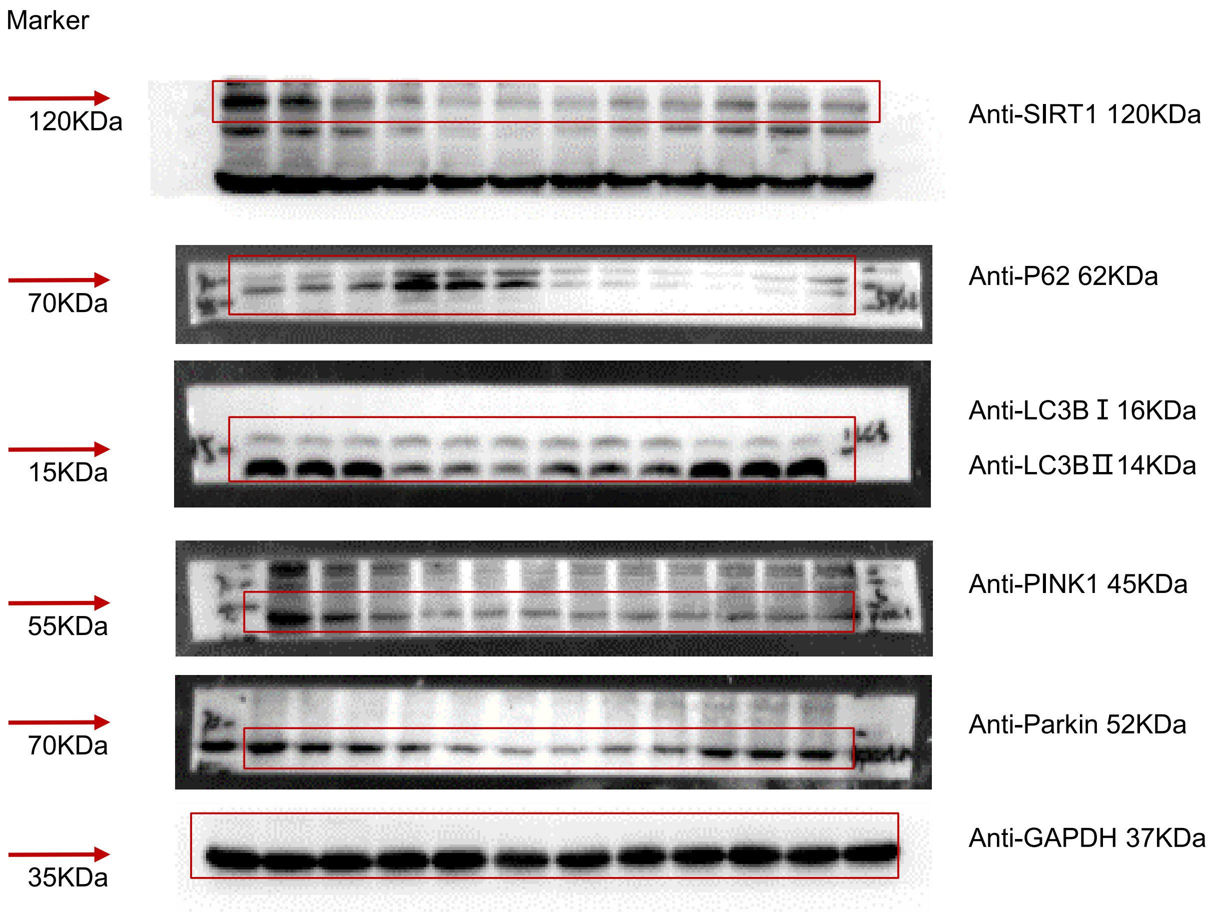

Full unedited blots for Supplymentary Figure 12C.

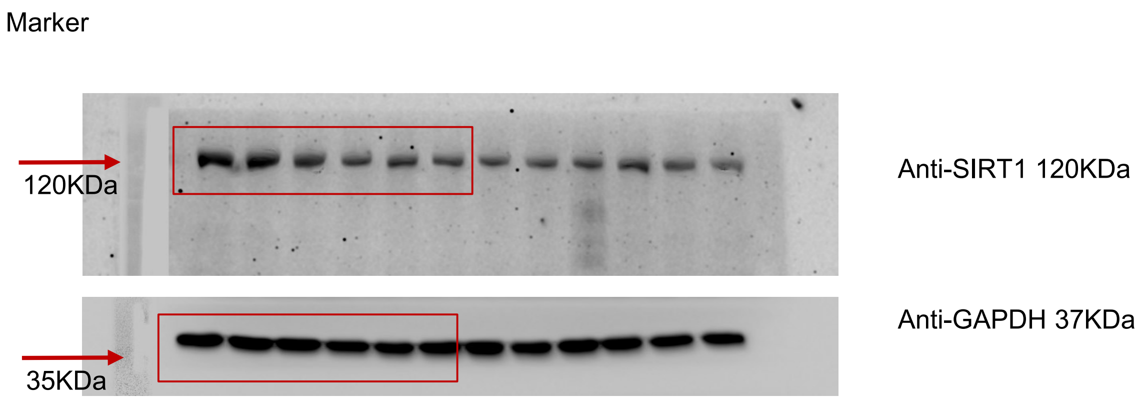

Full unedited blots for Supplymentary Figure 12D.

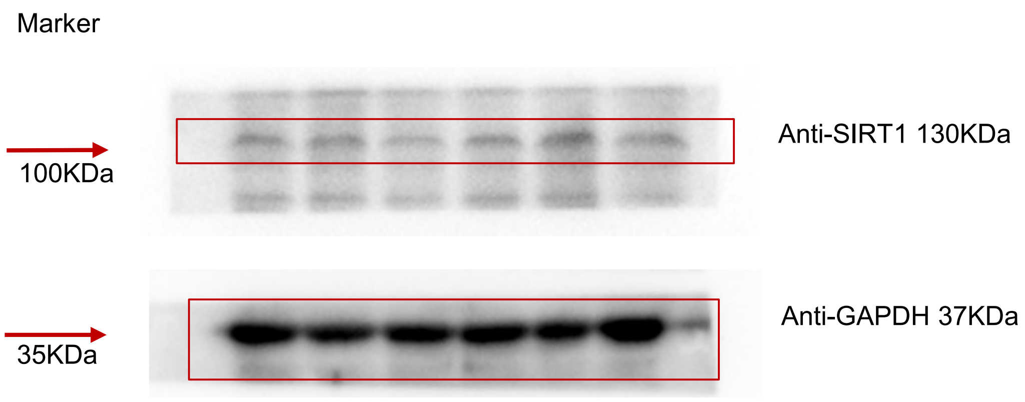

Full unedited blots for Supplymentary Figure 14I.

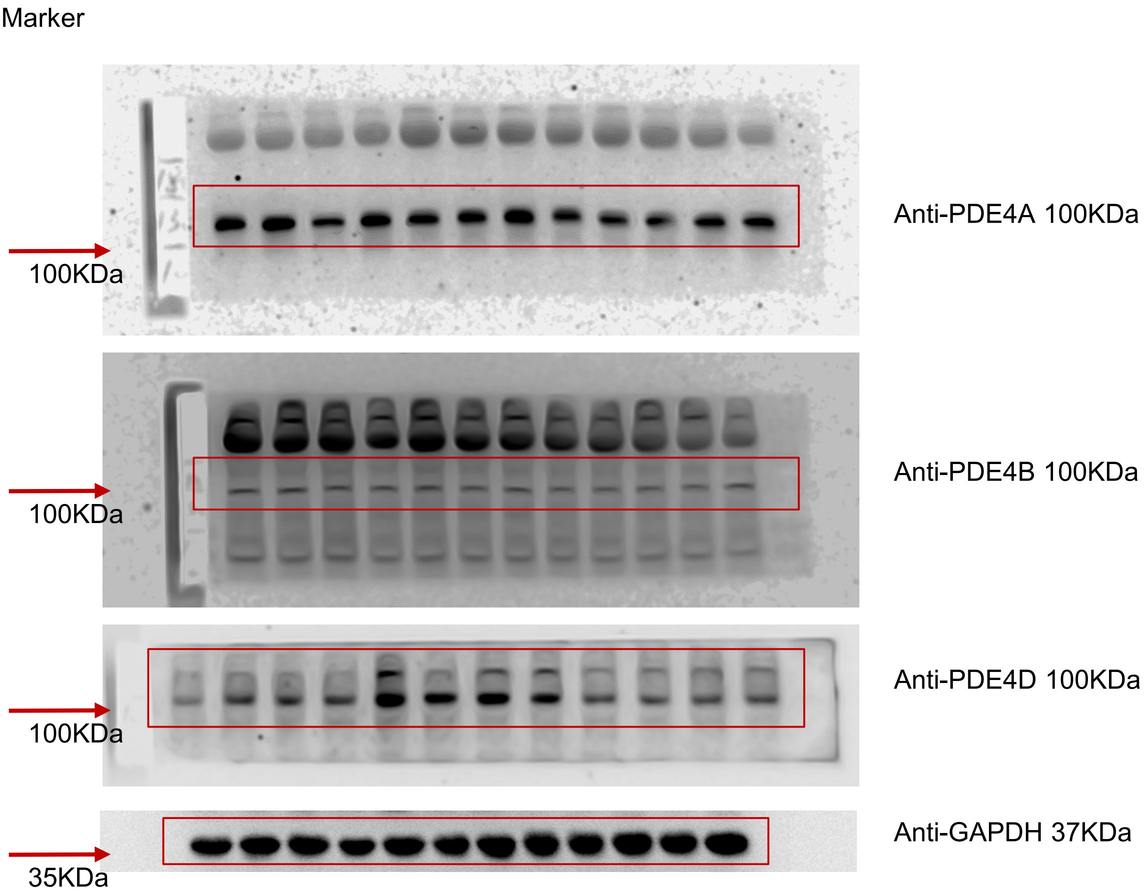

Full unedited blots for Supplymentary Figure 15B.

Marker

55KDa

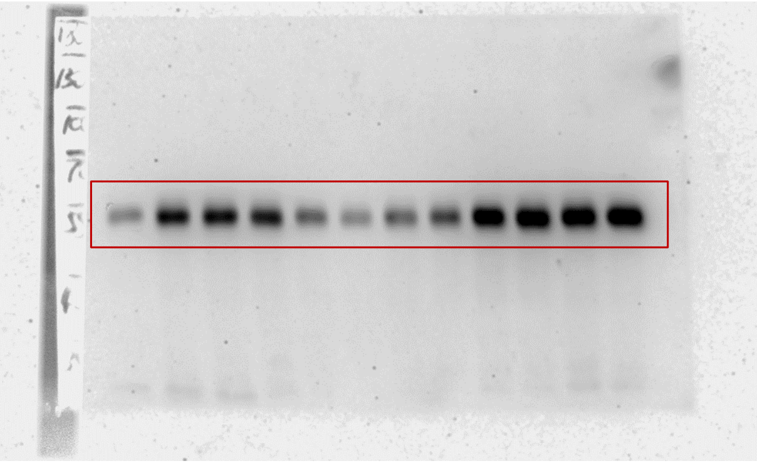

Anti-Parkin 52KDa

100KDa

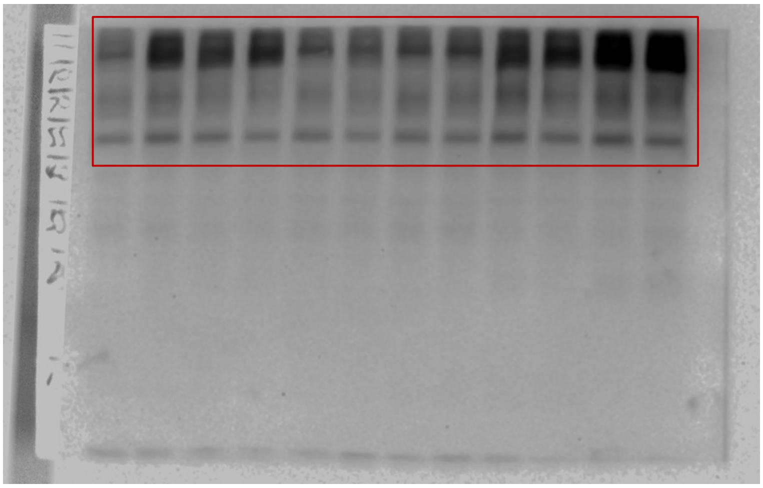

Anti-ubiquitin 6-180KDa

Full unedited blots for Supplymentary Figure 17A.

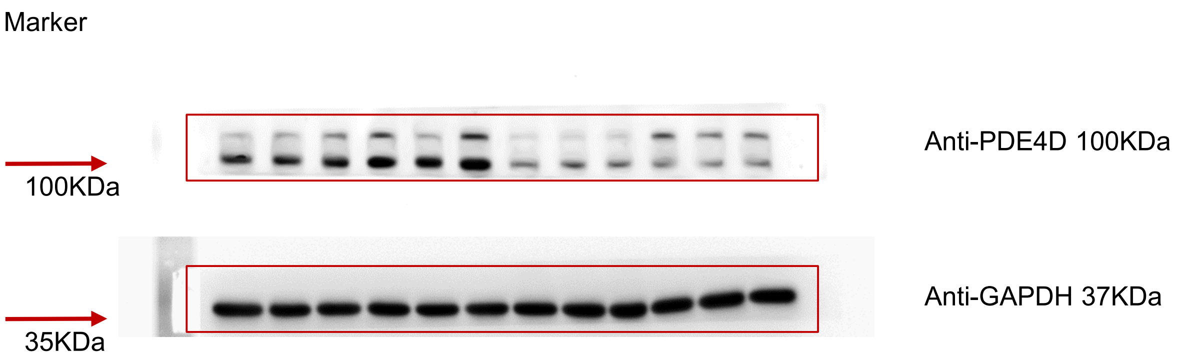

Full unedited blots for Figure 21A.

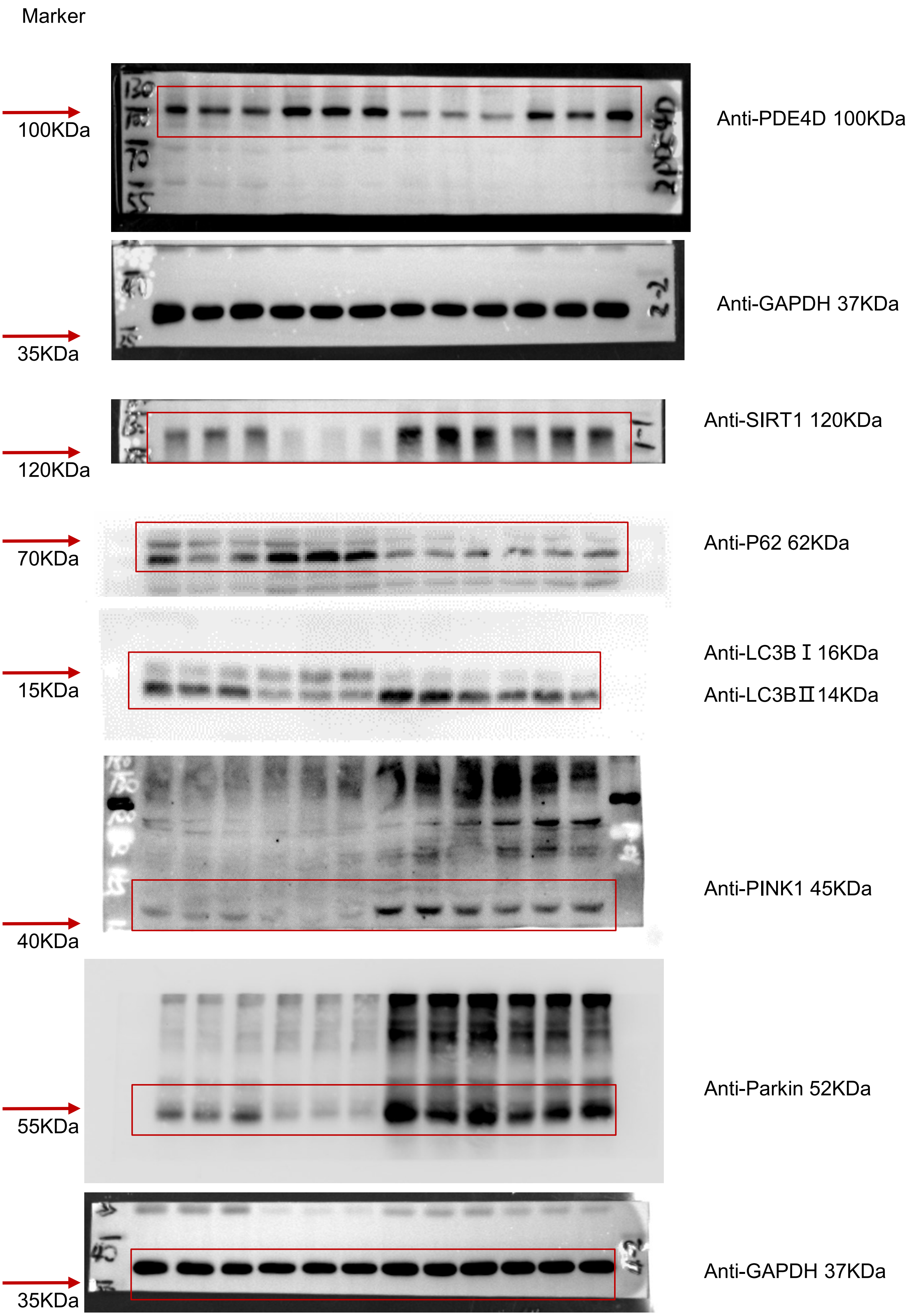

Supplement: Multimedia component 2 [file mmc2.pdf]
